# Nanomaterials in the Environment: Research Hotspots and Trends

**DOI:** 10.3390/ijerph16245138

**Published:** 2019-12-16

**Authors:** Chen Li, Guohe Huang, Guanhui Cheng, Maosheng Zheng, Nan Zhou

**Affiliations:** 1 Sino-Canada Resources and Environmental Research Academy, North China Electric Power University, Beijing 102206, China; lichen1368@outlook.com; 2 Center for Energy, Environment and Ecology Research, UR-BNU, Beijing Normal University, Beijing 100875, China; 3 Institute for Energy, Environment and Sustainable Communities, University of Regina, Regina, SK S4S 0A2, Canada; guanhuicheng@gmail.com; 4 School of Environmental Science and Engineering, North China Electric Power University, Beijing 102206, China; maoshengzheng@ncepu.edu.cn (M.Z.); znan1995@163.com (N.Z.)

**Keywords:** environment, nanomaterials, bibliometrics, citespace

## Abstract

Research on the field of nanomaterials in environment has continued to be a major area of interest in recent years. To present the up-to-date progress in this field, a bibliometric study is conducted to analyze 7087 related publications in the Science Citation Index (SCI) core collection of Web of Science based on the expanded SCI. These publications are identified through using representative keywords in the research directions environment of the Web of Science. This study finds that China and the United States dominate the field; one difference between them is that China issued more independent publications and the United States issued more cooperative publications. In addition, the number of the related publications in Asian countries has exceeded that of European and American ones. A Chinese institution, the Chinese Academy of Sciences, has an absolute dominance in this field. Traditional high-impact environmental journals have ruled this field. The number of publications in the Energy and Environmental Science field has gradually decreased. In addition, a co-citation analysis shows that previous studies in this field can be divided into four major branches, and that graphene oxide and nano-inorganic particles are increasingly becoming research hotspots.

## 1. Introduction

Gleiter et al. [1] developed the first method for producing nanomaterials. Afterwards, the development of technological advancements and engineering applications of nanomaterials was attempted in various fields (e.g., chemistry, physics, and medicine) [2]. For example, nanomaterials have become a hot topic in environmental studies due to their large specific surface areas, unique micro-interface characteristics, and appealing pollution remediation capabilities [3]. Meanwhile, nanoparticles can affect biological behaviors at the cellular, subcellular, and protein levels, while some readily travel throughout and deposit in the body, target organs, cells, and mitochondria and may trigger injurious responses. As a result of extensive applications and potential environmental risks [4], nanomaterials in the environment (NME) have gained increasing attention from of researchers all over the world over the past decades. 

Specifically, a series of nanomaterials-related studies have been conducted in environmental sciences and engineering. For instance, Gottschalk et al [5] simulated environmental concentrations based on a probabilistic analysis from a life-cycle perspective of NME-containing products. Mortimer et al [6] evaluated the toxic effects of nanoparticles of ZnO and CuO with a particle-ingesting model of tetrahymena thermophila [7]. Hu et al. [8] studied the batch removal of hexavalent chromium from aqueous solution by using oxidized multiwalled carbon nanotubes [9]. Nowadays, covalent organic frameworks and metal organic frameworks are a hotspot in environmental studies. Accordingly, the developmental process of NME-related technologies, theories, and methods has come to constitute a complex network [10]. An analysis of this network is helpful for guiding future development of NME studies, but this has not been conducted. 

To achieve such an analysis, bibliometrics, i.e., a statistical analysis of existing literature in a comprehensive knowledge system, is a potentially effective method. In this method, mathematics, statistics, and philology are integrated for quantifying literature characteristics [11]. Bibliometrics can provide researchers with a comprehensive developmental image and possible developmental trends [12]. It can also identify distributional features of research, e.g., spatial distributions of publications, researchers, countries, and institutions. For instance, Leung et al. [13] employed two bibliometric analysis methods to provide a systematic and holistic review of social-media-related academic literature. Liao et al. [14] explored the status of medical big data through a visualization analysis of the journal articles related to membrane bio-reactors (MBR). Zheng et al. [15] provided insights into publication performances and research trends of ammonia oxidation. 

Furthermore, it is necessary to analyze the correlation of word frequencies, especially for the subjects of multiple branches of studies. A few studies of coupled analyses of word frequencies have been reported. For example, Li et al. [16] evaluated MBR-related literature from 1982 to 2013 by using a new word cluster analysis method. Huai et al. [17] analyzed water security through an analysis of word frequencies in a bibliometric framework. Leung et al. [13] improved the bibliometric reliability of social media research with a co-citation and co-word analysis. However, previous NME-related bibliometric studies ignore the potential information that word frequencies can provide [18]. Lack of NME-related word-frequency analyses may reduce the comprehensiveness of discipline development analyses and increase the uncertainty of trend analyses [19]. 

Therefore, this study focuses on a bibliometric analysis of literature regarding NME (i.e., nanomaterials in the environment) [20]. Based on literature information collection, the analysis consists of representative bibliometric methods, a word-frequency correlation analysis, a clustering analysis, and a co-citation analysis. Research trends of literature publications, countries, institutions, research directions, and journals are quantified. This study will help reveal potential correlations and developments in NME studies and improve the reliability of original bibliometrics.

## 2. Data

NME-related publications are searched from the database of Web of Science Core Collection. The search-related parameters and strategies are set as follows. Specifically, the “Science Citation Index Expanded (SCI-EXPANDED) 1900-present” is selected as the Citation Indexes in More Settings [18]. Related publications were downloaded on 23 April 2018. A series of combinations of keywords were constructed for searching publications. Representative ones include “nanomaterial*” or “nano-metal oxide*” or “nano metal*” or “nanotube*” or “quantum dot*” or “quantumdot*” or “C60” or “C70” or “fullerene*” or “SWCNTs” or “MWCNTs” or “graphene” or “nanosheet” or “nano tube*” or “nano-zinc” or “nano-iron” or “nano-aluminium” or “nano-copper” or “nano-gold” or “nano-carbon” or “nano-Ni” or “nano- nickel” or “nano-Ag” or “nano-Au” or “nano-Cu” or “nano-Al” or “nano-Fe” or “n-Fe” or “nano-titanium” or “nano-Ti” or “n-Ti” or “nano-Zn” or “n-Zn” or “nano-CdSe” or “n-CdSe” or “nano-ZnS” or “n-ZnS” or “nano-CdTe” or “n-CdTe” or “nano-TiO2” or “n-TiO2” or “nano-Al2O3” or “n-Al2O3” or “nano-Fe2O3” or “n-Fe2O3” or “nano-Fe3O4” or “n-Fe3O4” or “nano-ZnO” or “n-ZnO” or “nano-CuO” or “n-CuO” or “nano-silver” or “nano ZnFe2O4”.

To limit the scope of types of publications, some empirical factors were selected in this study [21]. The Document Type is set as “Article” in the Result Refine, the Research Area as “Environmental Science and Ecology”, and the Publication Years as “2004 to 2017”. In order to obtain a usable document format, the Plain Text file of the Full Record and Cite References was selected as the transfer file. In the end, a total of 7087 publications were identified. The records of these publications contained authors, titles, keywords, keywords plus, abstracts, countries and regions, disciplines, journals, research institutions, publication time, references, and codes. Among this information, the country and region information was obtained from author affiliations, and the keywords plus are the words with the highest citation frequencies provided by Web of Science [22].

## 3. Method

### 3.1. Holistic Approach

In this research, Excel and R language (R) were the main tools, while other tools were used to assist, calibrate, and visualize. Excel is powerful in filtering information. Many simple statistical analyses are completed by Excel [23]. R is also excellent in data analyses, especially for efficiently analyzing big data. All sophisticated and cumbersome calculations in this study were accomplished by using R. In addition, calculation results were visualized by Excel and R. 

Citespace was used for the co-citation analysis in this research. Citespace is a visual analysis tool that focuses on the correlations between references cited by high-impact publications, and can present the relationships between different research branches [24]. In addition, a wealth of discipline trends can be obtained through the interpretation and analysis of the Citespace results. NME has multiple research branches whose development processes are unclear. Therefore, the research results of the co-citation analysis in the correlation and disciplinary trends of each branch were of great significance to NME. 

Some software such as Pajek [25] and VOSviewer [26] can also visualize results to supplement research. Various visualization results can show the developmental momentum of nanomaterials and the influence of authors, institutions, and countries in the field of environmental research. In addition, some other tools help to calculate a few indicators (such as Histcite), and they are noted when they were applied. 

**Figure 1:**
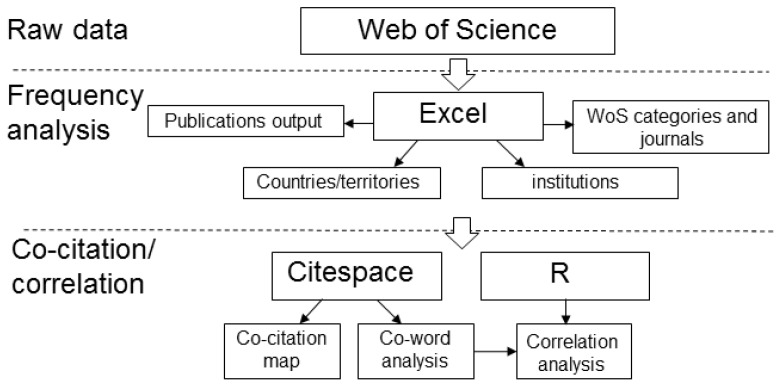
Flowchart of this research.

The detailed steps of a holistic approach are illustrated in Figure 1.

- Step 1: Raw files in plain texts were automatically merged into available Excel files with abbreviated titles by Bibexcel [27] (a simple analytical tool). In the file, different items were separated by tab-delimited for facilitating importing into Excel.
- Step 2: The numbers of publications by various countries, institutions, journals and authors were filtered by Excel.
- Step 3: Relationship of co-citation (i.e., two articles were cited by another article at the same time) was analyzed with Citespace for the Excel files produced in Step 1.
- Step 4: High-frequency words in all publication titles were counted in R. A co-word analysis provides the words frequency of co-occurrence to analysis the value of words. Afterwards, a correlation analysis was delivered by R for revealing the relationship of the high-frequency words.

### 3.2. A Frequency Analysis of Words in Titles

Many potential relationships exist for the variations of high-frequency words with different years (e.g., positive correlation, negative correlation, or no correlation). Therefore, it was necessary to analyze the correlation of high-frequency words comprehensively. 

In this study, the correlation between any two selected high-frequency words was analyzed by R. The map of all correlation includes the following information: Pearson Correlation Coefficients in the upper triangle (i.e., the digital matrix on the map of the upper right of the main diagonal), frequency distributions of variables (e.g., the words) in the main diagonal, and scatter diagrams (i.e., the diagrams on the map of the lower left of the main diagonal). Among them, the Pearson Correlation Coefficient between variables X (e.g., the word X) and Y (e.g., the word Y) was calculated by [28]:

(1) $$\mathrm{PCC}(x,y)=\frac{n\sum_{i=1}^{n}x_{i}y_{i}-\sum_{i=1}^{n}x_{i}\sum_{i=1}^{n}y_{i}}{\sqrt{n\sum_{i=1}^{n}x_{i}^{2}-{\left(\sum_{i=1}^{n}x_{i}\right)}^{2}}\sqrt{n\sum_{i=1}^{n}y_{i}^{2}-{\left(\sum_{i=1}^{n}y_{i}\right)}^{2}}}$$

where x_i_ is the word frequency at the ith year and y_i_ is the word frequency at the ith year. A scatter diagram can describe whether the correlation between two variables (i.e., words) is linear correlation or discrete relation (i.e., irregular distribution). A frequency distribution shows the frequency of the variable at each level (i.e., the abscissa interval of each bar was a level). In this study, several high-frequency words with research value (i.e., selected words through co-word analysis) were selected for a correlation analysis. We choose nine words with research value from all initially obtained words. 

## 4. Frequency Analysis of Publication Outputs

### 4.1. Publication Outputs

To understand the research progress of nanomaterials in the environmental field, the characteristics of extracted publications from 2004 to 2017 are shown in Table 1. The number of annual publications has gradually increased from 27 (2004) to 1276 (2017). The number of annual authors increased from 188 (2004) to 7035 (2017). The growth of this field has been rapid. The prominent growth, i.e., an increase of 187 compared with the previous year, happened in 2011. The growth rates in 2012 and 2013 decelerated. After 2013, the growth rate stably accelerated until 2017. It can be discovered from the AU/TP that the fastest growth of the discovery of this field occurred in this period. 

There are 499 high-cited publications (i.e., cited for more than 35 times) in this field, 99 of which were published in the journal of Environmental Science and Technology. An increase in the total global citation score (i.e., TGCs) indicates the increasing influence of NME [29]. Among the 7087 publications, 13, 3, 2, and 1 were published in the languages of German, Spanish, Polish, and French, respectively, while the remaining 7068 (99.73%) in English. All non-English publications contain an English headline, abstract, and author keywords. TGCs and TLCs (Table 1) peaked in 2011 and 2009, respectively. The time to collect citations was considered. From 2009 to 2011, local citations of NME decreased. This may indicate that the development of studies in this field slowed down. Meanwhile, the proportion of high-influence discoveries declined during this period.

### 4.2. Countries/Territories and Institutions

The 7087 publications in this research were from 76 countries and territories, and 5,201 (73.4%) publications were without international cooperation from 65 (85.5%) countries and territories. Table 2 lists some of the statistics on the number of publications in the top fifteen countries. Six of the top fifteen countries were Asian countries and territories, and the top five included four Asian countries. Six of them were from Europe, while two were from North America countries and only Australia was from Oceania. The total number of publications in the G20 countries were 2490 (35.13%) in China, 1998 (28.19%) in the US, 17 (0.24%) in Argentina, 186 (2.62%) in Australia, 118 (1.67%) in Brazil, 210 (2.10%) in Canada, 220 (3.10%) in France, 247 (3.49%) in Germany, 298 (4.20%) in India, 201 (2.84%) in Italy, 170 (2.40%) in Japan, 359 (5.07%) in South Korea, 36 (0.51%) in Mexico, 41 (0.58%) in Russia, 121 (1.71%) in Saudi Arabia, 52 (0.73%) in South Africa, 127 (1.79%) in Turkey, 287 (4.05%) in the UK, and 4 (0.06%) in Indonesia. It can be seen that the influence of developing countries in this field was no less than that of developed countries in Europe and America [30].

China and the United States play a leading role in the research of nanomaterials in the environmental field. The total number of publications published by the two countries were 4,488 (63.28%), including 1762 publications issued by China alone, 728 publications involving international cooperation with China, 1155 publications by the United States alone, and 843 publications involving international cooperation with the US. China has experienced a rapid development in this field from seven publications written by China alone in 2004 to 303 publications in 2017. The United States also grew from seven publications published by the US alone in 2004 to 74 publications in 2017. This data cannot completely confirm that China was ahead of the United States in terms of research in these areas. The United States has a large number of collaborative research publications with other countries. However, the data shows that China is indeed developing rapidly in this field due to the support and investment of the Chinese government.

**Figure 2:**
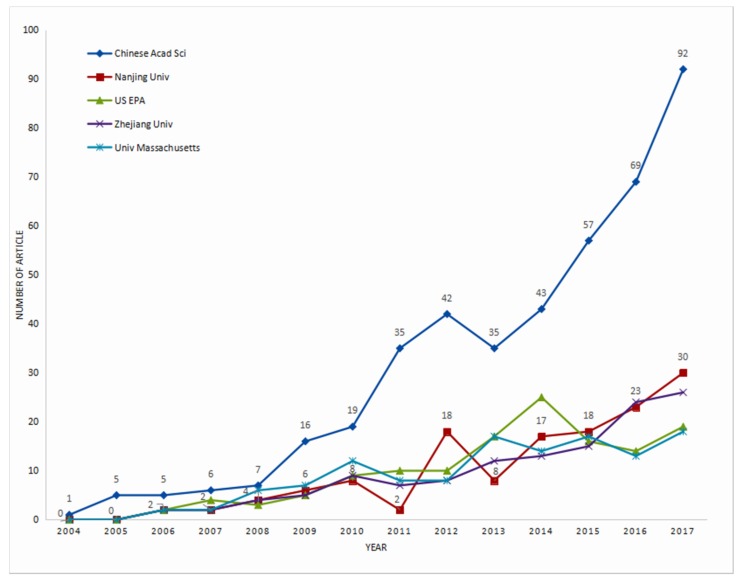
Growth trends of annual publications from the top five institutions. The number of institutions of the top eight productive countries respectively are 1343 for China, 1476 for USA, 160 for South Korea, 173 for Iran, 132 for India, 98 for England, 89 for Germany, and 94 for France.

According to Figure 2, 3907 institutions in total participated in the research of the NME. Among them, 1808 publications were issued by independent institutions. China ranked first with 627 publications (accounting for 8.85% of total and 34.7% of total IIPs), and US ranked second with 341 publications (accounting for 4.81% of total and 18.86% of total IIPs). In addition, the top five institutions of independent publications in this research respectively were Chinese Acad Sci (432), Nanjing Univ (138), US EPA (134), Zhejiang Univ (127), and Univ Massachusetts (124). Among them, independent publications by Chinese Acad Sci accounted for 6.10% of the total publications and 23,89% of the total independent publications, which was much higher than the second place of Nanjing Univ (which respectively accounts for 1.95% and 7.63%). Chinese Acad Sci, Nanjing Univ, and Zhejiang Univ had a major influence on NME in China, while the US EPA and Univ Massachusetts showed a major representation in the United States, respectively accounting for 6.7% and 6.2% of the total US publications. In this statistic, more than 100 offices across the country were not included in Chinese Acad Sci, so its actual influence should be greater. 

**Figure 3:**
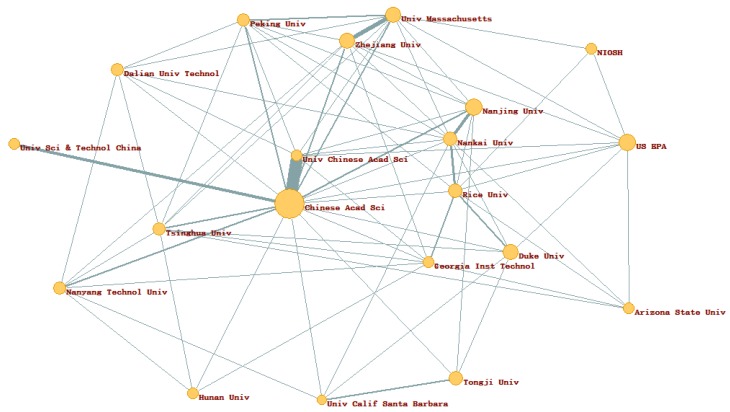
Partnership map of top 20 institutions (plot by Pajek). The size of circle was the number of an institution; The width of the line was the number of cooperation achievements. Moreover, visualizations of institution are shown in Figures S2 and S3.

As shown in Figure 3, if the number of publications involving cooperation by two institutions was significantly larger than that of publications where they cooperate with other institutions, it may represent a traditional friendship between these two institutions [31]. For example, 68 publications were published through cooperation between Chinese Acad Sci and Univ Chinese Acad Sci in NME, which was much higher than them with other institutions. The number of cooperation publications between Univ Massachusetts and Zhejiang Univ was 22, which was also higher than with others. Meanwhile, there were multiple examples of collaboration in most institutions. Most institutions have at least five partners, as shown in Figure 3. 

### 4.3. Web of Science Categories and Journals

**Figure 4:**
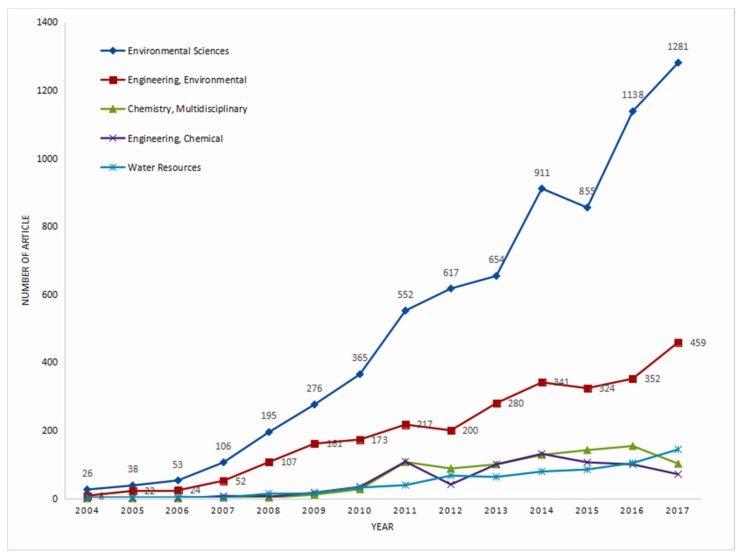
Growth trends of annual publications from the top five Web of Science categories.

To understand the trend and variation of the research areas of NME, 7087 publications from Web of Science categories (i.e., the categories of Research Areas of Web of Science) and journals were analyzed in this study. These publications were from 43 Web of Science categories of which the two most important categories (Figure 3) were “Environmental Sciences” (7067, 99.7%) and “Engineering, Environmental” (2720, 38.4%). Other categories containing many publications (≥ 500) included “Chemistry, Multidisciplinary” (863, 12.2%), “Engineering, Chemical” (721, 10.2%), “Water Resources” (656, 9.3%), “Toxicology” (644, 9.1%) and “Energy and Fuels” (588, 8.3%). Due to the limitations of the retrieval method, almost all publications were from “Environmental Sciences”. Therefore, “Environmental Sciences” had an absence of analysis values in this section. The following analysis does not consider “Environmental Sciences”. The main categories in this field can be divided into engineering, chemistry, chemical engineering, and water resources (i.e., exclusive of “Environmental Sciences”). Among them, engineering ranked first, because most of the findings of NME were significant for engineering applications. The other three categories were in an interlaced rise. From the trend in the Figure 4 and the current emphasis on water resources, it can be judged that the growth of water resources may continue. Whether water resources will tremendously increase depends on the breakthrough in the research of this category in the future.

**Figure 5:**
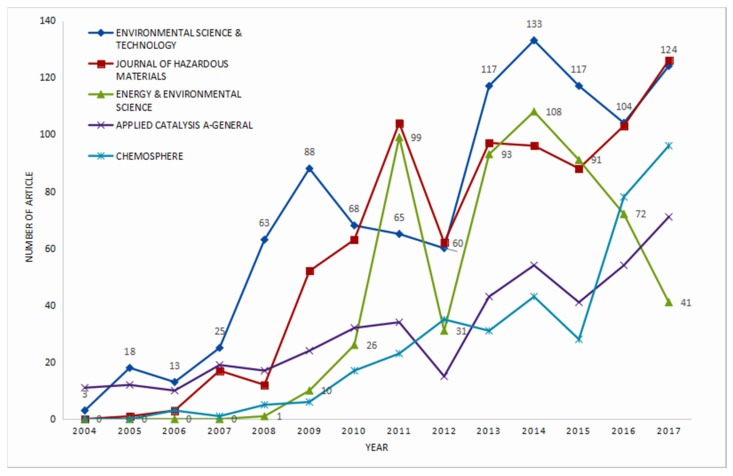
Growth trends of annual publications from the top five journals. Other visualizations of categories are shown in Figures S4 and S5.

The 7087 publications analyzed by this research were published in 166 journals. There are 22 journals in which more than 50 publications have been published, with the total of 5554 publications (78.4%). There are five journals with more than 300 publications published in the field, namely, ENVIRONMENTAL SCIENCE and TECHNOLOGY (i.e., EST) (998, 14.1%), JOURNAL OF HAZARDOUS MATERIALS (i.e., JHM) (824, 11.6%), ENERGY and ENVIRONMENTAL SCIENCE (i.e., EES) (572, 8.1%), APPLIED CATALYSIS A-GENERAL (i.e., ACA)(437, 6.2%), and CHEMOSPHERE (i.e., CHP) (366, 5.2%). Among them, only EES showed a significant downward trend in the past three years, which may be related to its variation of research orientation. It may be more difficult for NME publications to be submitted to EES. The other four journals still show an upward trend, although their fluctuations are erratic. There are 95 (57.2%) unpopular journals in which the number of publications is not greater than 10; 278 (3.9%) publications were published in these journals. This section demonstrates the variation of attention for these journals in NME [32]. The attention of JHM, ACA, and CHP may continue to increase in NME based on the trend of Figure 5 alone. But there was major uncertainty for the trend of EES and EST.

## 5. Co-Citation and Word-Correlation Analyses

### 5.1. A Co-Citation Analysis

**Figure 6:**
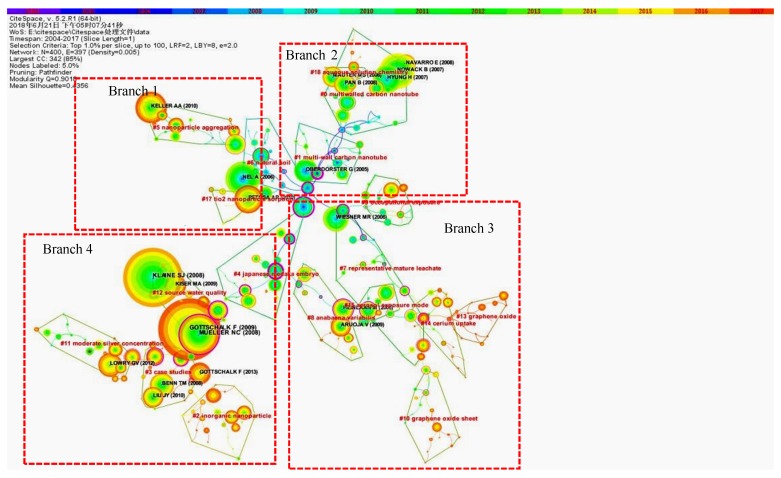
Cluster view of the co-citation analyzed by CiteSpace. In the cluster view, the size of the concentric ring represents the co-citation frequency; the thickness of the concentric ring represents the co-citation frequency (i.e., when the number of co-citation is increased once, the ring is increased slightly); the color of the concentric ring represents the cited year (i.e., the color of year in figure legend); the color of connected line means the time when the two publications are both cited by a same publication for the first time; and node spacing represents the relevance of publications.

A co-citation analysis [33] proved to be a more effective and comprehensive tool than an author keywords analysis to reveal the research trends and to discover hotspots in a given research field. As shown in Figure 6, a co-citation analysis was performed on the publication citation network from 2004 to 2017. The time slice was found to be 1. Top 400 levels of mostly cited items from each slice were selected. Each level may include multiple qualified nodes. Top 1% of most cited item from each slice were selected. A pruning method was Pathfinder. These publications are considered to have major influences in this field (Figure 6). Publications with the meaning of turning points (i.e., the starting point of a new cluster and a publication of high cited times) are marked as a purple-red outer ring. 

7087 publications are divided into 18 clusters. Each cluster has a clear meaning with the Contour coefficient of 0.92 to 0.99. The map as a whole was represented by a center-to-around divergent pattern. This was consistent with the research progress gradually developed from the preliminary research to multiple branches. Moreover, cluster and network summary of co-citation analysis are shown in Tables S1 and S2. 

As shown in Figure 6, branch 1 contains #5 nanoparticle aggregation, #6 natural soil, and #17 TiO2 nanoparticle sorption. The publications in branch 1 focused on the physical and physicochemical properties of nanoparticles. Branch 2 includes #1 multi-wall carbon nanotube, #0 multiwall carbon nanotube, and #18 aqueous solution chemistry. The publications in branch 2 are the studies regarding multi-walled carbon nanotubes. The branch 3 includes #9 occupation exposure, #7 representative mature leachate, #8 anabaena variables, #15 aerosol exposure mode, #14 cerlum uptake, #13 graphene oxide, and #10 graphene oxide sheet. The publications in branch 3 are applications of nano-risk and nanomaterials for decontamination. Branch 4 includes #4 Japanese medaka embryo, #12 source water quality, #11 moderate silver concentration, #3 case study, and #2 inorganic nanoparticle. The publications in branch 4 are mainly the research on the influences of inorganic nanomaterials on water quality and aquatic organisms. In Figure 6, there was a correlation between publication dates and the distance from the center to the point of publication. (i.e., the farther away from the center, the later; otherwise, the earlier). Most of publications in cluster #1, #4, #6, and #17 are published in 2004 to 2009. The cluster #2, #3, #5, #10, and #13 appear in the recent years. Publications in these clusters mostly appear from 2014 to 2017.

As shown in Figure 6, three main critical nodes are in the central area. The brain effect of C60 on perch is published by Oberdörster et al in Environment Health Perspective in 2004 [34]. A comparative research cytotoxicity of three nanocarbons is published by Jia et al. [35] in Environmental Science and Technology. Lecoanet et al. [36] focused on the mobility of nanomaterials in porous media which were published in Environmental Science and Technology in 2004. Different branches of nanomaterial research in the environmental field are linked based on these four early studies of great impacts.

The key finding in Branch 1 is the physiological effect of C60 on the brain of perch that was published by Fortner et al. [37]. An important achievement is that the rate of aggregation of C60 particles is greatly reduced in the presence of humic-acid and electrolytes in cluster nanoparticle aggregation [38]. The cluster TiO2 nanoparticle sorption is separated from cluster nanoparticle aggregation due to trimming secondary connections in this branch. However, the physicochemical interaction of nanomaterials in the aquatic environment is mainly researched in cluster TiO2 nanoparticle sorption [39]. 

In Branch 2, two clusters of multi-wall carbon nanotubes are the main component of this part. Among them, the most influential findings are the behaviors of nanoparticles throughout the cycle [40], which is a significant research. 

Branch 3 developed progressively. On the center of Figure 6, cluster representative mature leachate mainly researches the percolation and migration of nanomaterials in quartz sand and other materials. Cluster cerium uptake is based on the cluster of representative mature leachate. Two clusters are on the leaf of Branch 3, while graphene oxide and graphene oxide sheet have many new hot spots. The research in the cluster #13 graphene oxide is initiated by the research of the colloidal properties and stability of graphene oxide in the aquatic environment. Other clusters in this branch are related to nanotoxicity and risk analyses, representing that research is the effects of nanometal oxides on aquatic organisms [41]. 

Branch 4 is the most influential branch of nanomaterials in the environmental field. This branch contains a number of high-impact researches, and some of the results have long-term influence. The two most cited publications in this research are from this branch. For example, the multi-nanomaterials multi-environment concentration model developed by Gottschalk et al. always has high influence in the field [5]. Japanese medaka embryo is an independent cluster, because it is mostly used for the research of the effects on aquatic organisms. Actually, all publications of this cluster study the nanotoxicity and risk of nanoparticles for laboratory animals. On the basis of this, specific research on the quality of water environment was developed, and continues to be a hotspot because of extensive public attention. In cluster #11, Savage et al [42] published a toxicity and stability study of nano-silver in the environment, which is the key research of this cluster. The simulated results and actual situation of nanomaterials in different water cases are analyzed in the cluster #3 case study [43]. The periodic and probabilistic model studies are hotspots in cluster #2 (i.e., inorganic nanoparticles), such as the research of emission probability models of nanomaterials [44].

**Figure 7:**
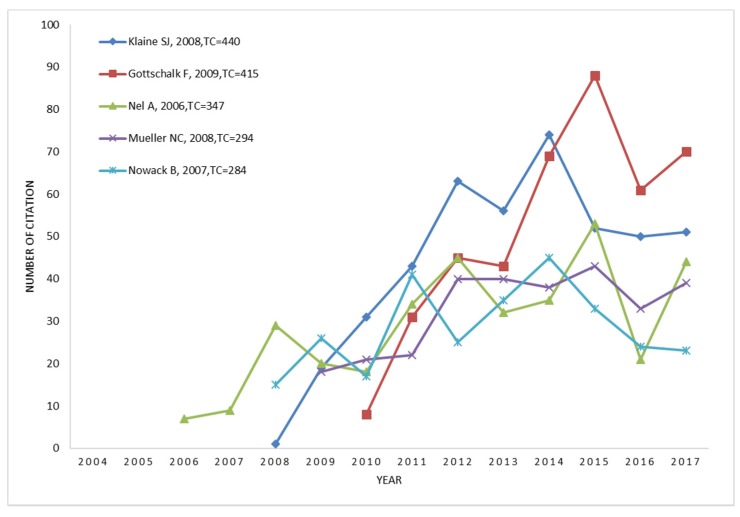
Growth trends of annual co-citation frequencies of the top five landmark works (Provide content that cannot be clearly displayed by the concentric circles).

In addition, a co-citation analysis is shown by a line chart (Figure 7) to provide a clear growth of the top five landmark works. Two citation growth patterns can be indicated from the line chart of five high co-citation publications, frequencies growth with high speed in a short time [40,45], and long-term stable citation [5,46,47]. 

**Figure 8:**
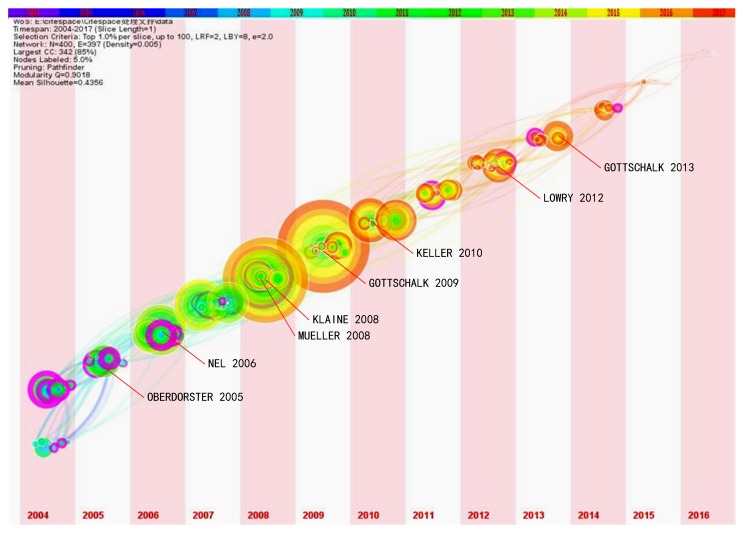
TimeZone view of co-citation analysis by CiteSpace. In the TimeZone view, the vertical grid of a publication indicates the published time of the publication; the size of the concentric ring represent the co-citation frequency; the thickness of the concentric ring represents the co-citation frequency (i.e., when the number of co-citation is increased once, the ring is increased slightly); the color of the concentric ring represents the cited date (i.e., the color of year in figure legend); the color of connected line means the time when the two publications are both cited by a same publication for the first time.

To further emerge the intellectual evolution of the NME research, the co-citation result was further described in a Timezone view (i.e., Figure 8), in which the publications were arranged chronologically according to the first year they appeared [15]. There are two flourishing stages in the NME research throughout the entire timespan, i.e., the period before and the period after 2009. These articles concentrated in the period before 2009 have less influence in the later NME research, reflected by their nods without the bright red outer ring. Findings with high impact mainly were published around 2009. These publications are a turning point in this period. Previous findings are rarely cited together with subsequent ones. The results of these publications have improved the existing research foundation at the previous stage. It is notable that four out of the five landmark works [5,40,45,46,47] focus on behaviors, fates, bioavailabilities, and effects of nanomaterials with a simple structure around 2009. The other [45] is the toxicity mechanism of nanomaterials on electrochemistry. The latest highly co-cited publications [48] investigate complex nanomaterials formatted by a metal-organic framework. In a word, research complexity of NME gradually increases in structure, impact, and cases. These results clearly demonstrate that more complex structure and details represent the trends in the NME [49]. 

### 5.2. Correlation of High-Frequency Words in Titles

**Figure 9:**
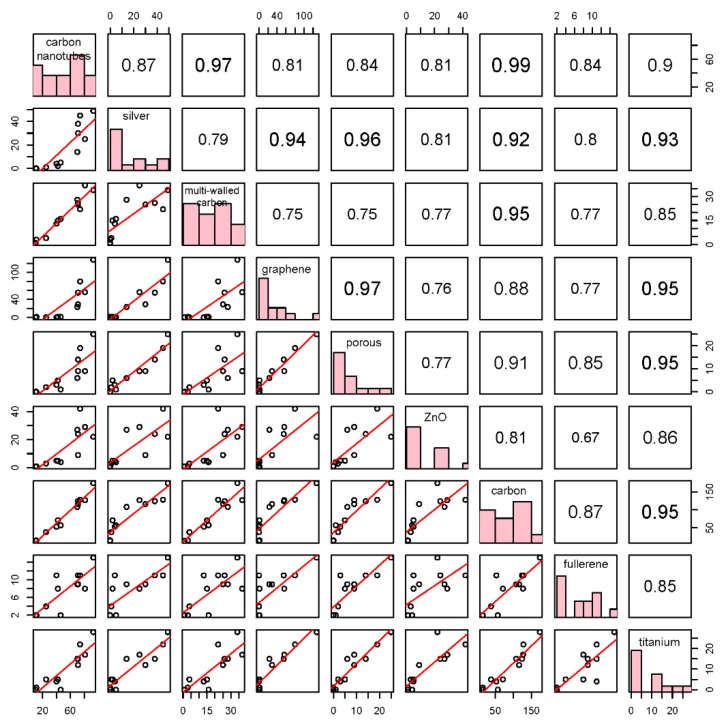
Distributions of and correlations between nine words in the title. PCC: result interval is from −1 to 1. When PCC equals ±1, the correlation is the highest (positive and negative relations). When PCC equals 0, two variables are uncorrelation. Frequency distribution: the frequency of the variable at each level of X axis. Scatter diagram: when points form a straight line, the two variables have a linear correlation. If the slope k is greater than 0, it has a positive correlation, otherwise, it has a negative correlation.

Many words in titles can express research contributions. An analysis of the variation correlation between high-frequency words can reveal the potential impacts of different contents. Firstly, based on occurrence frequencies, co-occurrence frequencies, and research significances, nine words (i.e., carbon nanotubes, silver, multi-walled carbon, graphene, porous, ZnO, carbon, fullerene, and titanium) were selected to analyze their correlations. To ensure the validity of the correlation analysis, a co-word analysis was applied to pledge that a publication only represents one research direction. As shown in Table 3, all words (except carbon nanotubes and multi walled carbon) are independent. The correlation graph of nine words is shown in Figure 9. The absolute value of correlation in the upper triangle (i.e., the digital matrix on the upper right of the diagonal line) matrix is the standard for classifying correlations. 

**Figure 10:**
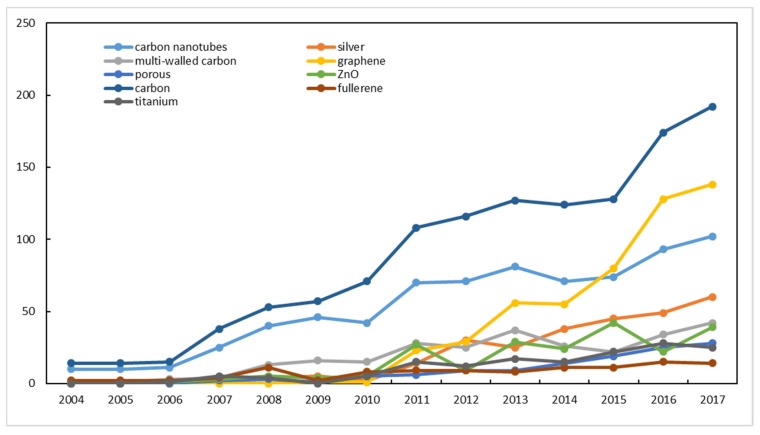
Numbers of high-frequency words from 2004 to 2017.

As shown in Figure 9 and Figure 10, most of words have significant positive correlations. It is intuitive that three carbon-related words have high correlations (0.97, 0.99, and 0.95). Carbon contains carbon nanotube and multi-walled carbon. In recent three years, graphene is increased rapidly, which becomes a main research object for carbon nanoparticles. Meanwhile, ZnO has the lowest correlation with other words and a lower annual frequency than another words. Therefore, ZnO may be a common research substance that is unsusceptible to hotspots. Silver, graphene, and titanium exhibit the highest correlations with the word porous. It shows that some of the three studies (Silver, graphene, and titanium) may adopt the same technical route and research method as with porous materials. Fullerenes develop steadily, and are not affected by graphene and other studies. In addition, there was a turning point around 2009. All words maintained a steady and slow growth in 2004–2009, but the frequency of words was in a state of rapid rise, unstable rise, or fluctuation in 2010–2017. 

NME has many research directions. Therefore, the co-word analysis cannot play an effective role in characterizing the relationships of words. The correlation analysis can prove to be an efficient tool in this area. 

## 6. Conclusions

In this bibliometric research, data of 7087 publications were analyzed which are recorded in the SCI core collection of web of science, and the citation index is selected as Science Citation Index Expanded (SCI-EXPANDED). The publications were searched by using restricted search terms and the research area of Web of Science. Analysis methods included frequency analysis, co-citation analysis, and correlation analysis of high frequency words. As summarized below, a series of findings regarding the bibliometric of nanomaterials in the environmental field were found. 

In particular, China and the United States dominate NME studies; China issued more publications independently, and the United States issued more publications cooperatively. The number of publications in Asian institutions (e.g., Chinese Academy of Science) has exceeded that of European and American countries, showing their absolute dominance in NME studies. Traditional high-impact environmental journals have ruled NME, and the number of publications in Energy and Environmental Science is gradually decreasing. A co-citation analysis suggests that the development history of NME studies can be divided into four major branches. Among them, more complex structure and details may be the research hotspots in NME. Organic or inorganic carbon nanoparticles may still have the strongest influence in NME. Moreover, correlation analysis is an efficient tool for word frequency analysis in bibliometrics.

In the future, we will continue to focus on this area. Meanwhile, we will explore the feasibility and practicability of more methods in bibliometrics, such as factor analysis and cluster analysis. 

## Figures and Tables

**Table 1:** Characteristics of the extracted publications from 2004 to 2017.

Year	TP	TLCS	TGCS	AU	AU/TP
2004	27	497	3121	188	6.96
2005	40	859	6672	191	5.03
2006	55	1487	8147	255	4.64
2007	110	1795	11,503	493	4.61
2008	203	4763	22,751	861	4.39
2009	278	4378	24,158	1264	4.53
2010	366	3334	24,546	1782	4.84
2011	553	3451	34,728	2672	4.82
2012	610	3799	34,475	3450	5.59
2013	707	2575	25,076	3439	5.25
2014	794	2623	22,928	4343	5.34
2015	941	1871	17,501	5212	5.46
2016	1127	891	11,656	6190	5.43
2017	1276	203	3866	7122	5.55

Note: TP means total number of publications, AU refers author numbers, AU/TP average author numbers per publication, TLCS total local citation score (i.e., TLCS: the number of cited times in the downloaded publication set) (TLCS and TGCS are calculated with Histcite), TGCS total global citation score (i.e., TGCS: the number of cited times in the whole Web of Science).

**Table 2:** Publication performances of the top 15 most productive countries.

Countries/Territory	TP	TP R		FP	FP R		IIP	IIP R		NII
China	2490	1	35.13%	2282	1	32.20%	627	1	8.85%	133
USA	1998	2	28.19%	1450	2	20.46%	341	2	4.81%	89
South Korea	359	3	5.07%	275	4	3.88%	70	5	0.99%	22
Iran	302	4	4.26%	288	3	4.06%	96	4	1.35%	34
India	298	5	4.20%	256	5	3.61%	100	3	1.41%	38
UK	287	6	4.05%	3	15	0.04%	0	14	0	0
Germany	247	7	3.49%	144	10	2.03%	30	9	0.42%	12
France	220	8	3.10%	127	12	1.79%	9	13	0.13%	4
Spain	211	9	2.98%	138	8	1.95%	30	9	0.42%	17
Canada	210	10	2.96%	157	6	2.22%	47	7	0.66%	23
Italy	201	11	2.84%	133	9	1.88%	20	11	0.28%	11
Australia	186	12	2.62%	94	14	1.33%	19	12	0.27%	9
Switzerland	184	13	2.60%	129	11	1.82%	26	10	0.37%	11
Taiwan, China	181	14	2.55%	156	7	2.20%	50	6	0.71%	23
Japan	170	15	2.40%	109	13	1.54%	36	8	0.51%	19

Note: TP is the total number of publications, TP R is the rank and the percentage of total publications, FP is the number of first authored publications, FP R is the rank and the percentage of first authored publications, IIP is the number of independent institution publications (i.e., the sums of publications absence of collaboration between research institutions in a country), IIP R is the rank and the percentage of independent institution publications, NII is the number of institutions with IIP. Moreover, visualizations of countries/territories are shown in Figure S1.

**Table 3:** Co-words analysis in Title, Abstract, Key words, and Key words plus from 2004 to 2017. There is no co- occurrence between silver, graphene, porous, ZnO, fullerene, and titanium. There is a high correlation between carbon nanotube and multi-walled carbon. Carbon contains carbon nanotube and multi-walled carbon.

	Carbon Nanotube	Silver	Multi-Walled Carbon	Graphene	Porous	ZnO	Carbon	Fullerene	Titanium
carbon nanotubes		0	129	0	0	0	602	0	0
silver			0	0	0	0	0	0	0
multi-walled carbon				0	0	0	0	0	0
graphene					0	0	0	0	0
porous						0	0	0	0
ZnO							0	0	0
carbon								0	0
fullerene									0
titanium									

## Supplementary Materials

The following are available online at https://www.mdpi.com/1660-4601/16/24/5138/s1, Figure S1 Countries/territories co-occurrence clustering network by pajek, Figure S2 Key words co-occurrence clustering network by pajek, Figure S3 Authors co-occurrence clustering network by pajek (1), Figure S4 Authors co-occurrence clustering network by pajek (2), Figure S5 Web of science categories co-occurrence clustering network by pajek, Figure S6 institutions co-occurrence clustering network by pajek, Figure S7 High-impact institutions of density view by VOSviewer, Figure S8 Hot Web of science categories of density view by VOSviewer, Table S1 Cluster summary of co-citation analysis by Citespace, Table S2 Network summary of co-citation analysis by Citespace.
