# Supplementary material for: Nanomaterials in the Environment: Research Hotspots and Trends
Source: Int J Environ Res Public Health. 2019 Dec 16;16(24):5138. doi: 10.3390/ijerph16245138 (PMC6950608; doi:10.3390/ijerph16245138)
Supplement: Supplementary file 1 [file ijerph-16-05138-s001.pdf]

Supporting Information

Nanomaterials in the environment: research hotspots and trends

Contents

Note： 2
Fig. S1 Countries/territories co-occurrence clustering network by pajek... 2
Fig. S2 institutions co-occurrence clustering network by pajek 2
Fig. S3 High-impact institutions of density view by VOSviewer... 3
Fig. S4 Web of science categories co-occurrence clustering network by pajek 3
Fig. S5 Hot Web of science categories of density view by VOSviewer.. 4
Table.S1 Cluster summary of co-citation analysis by Citespace... 4
Table.S2 Network summary of co-citation analysis by Citespace..51

Note:

In results of Pajek, the size of the point is the number of occurrences, and the width of the line is the number of simultaneous occurrences of the two connected points. In the density view, each point is filled the color according to the density of the them. High density is close to red, otherwise, blue. Density depends on the weight of surrounding term and itself.

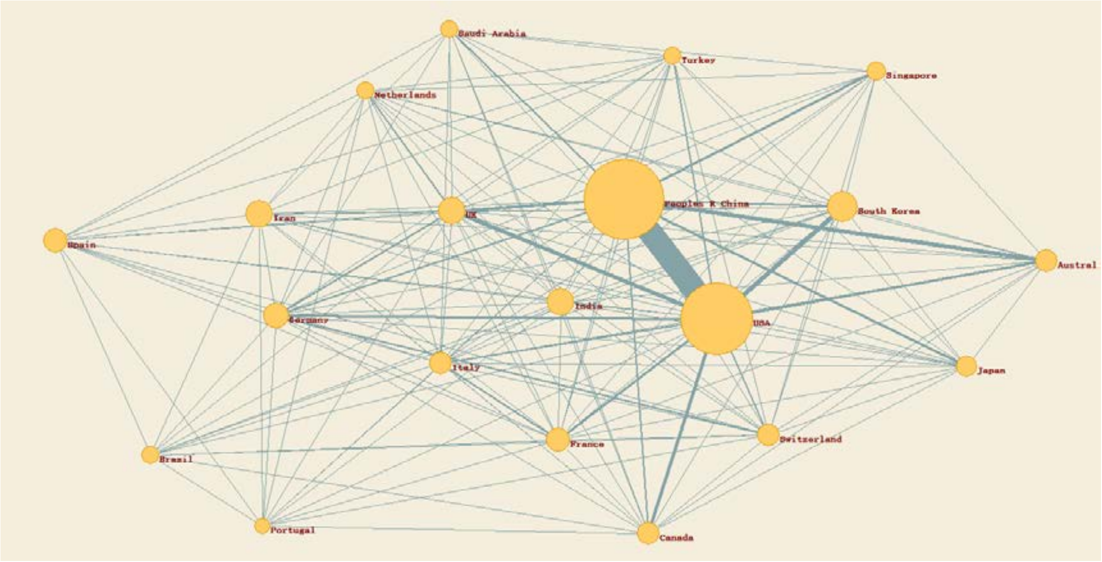

Fig. S1 Countries/territories co-occurrence clustering network by pajek

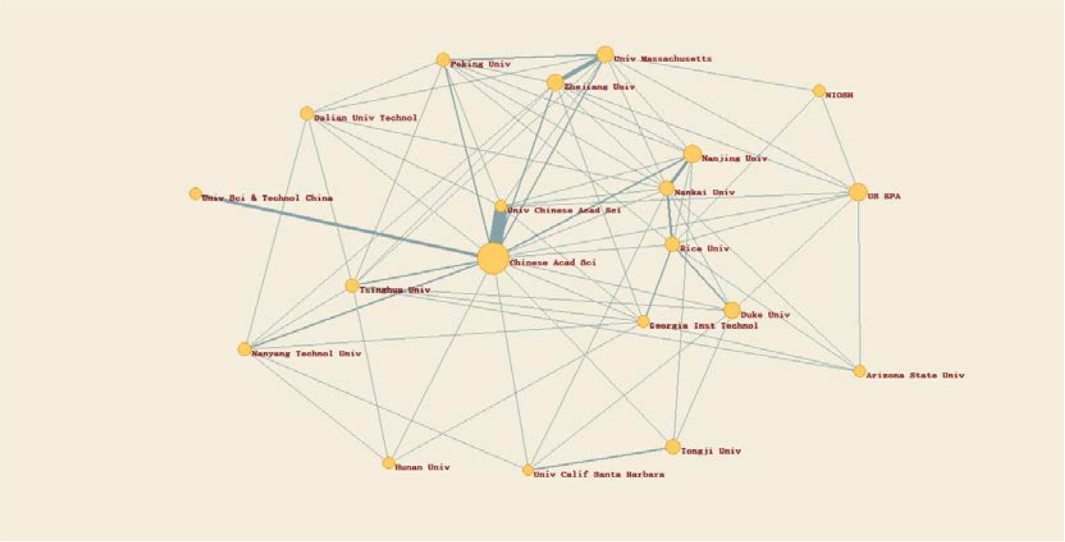

Fig. S2 institutions co-occurrence clustering network by pajek

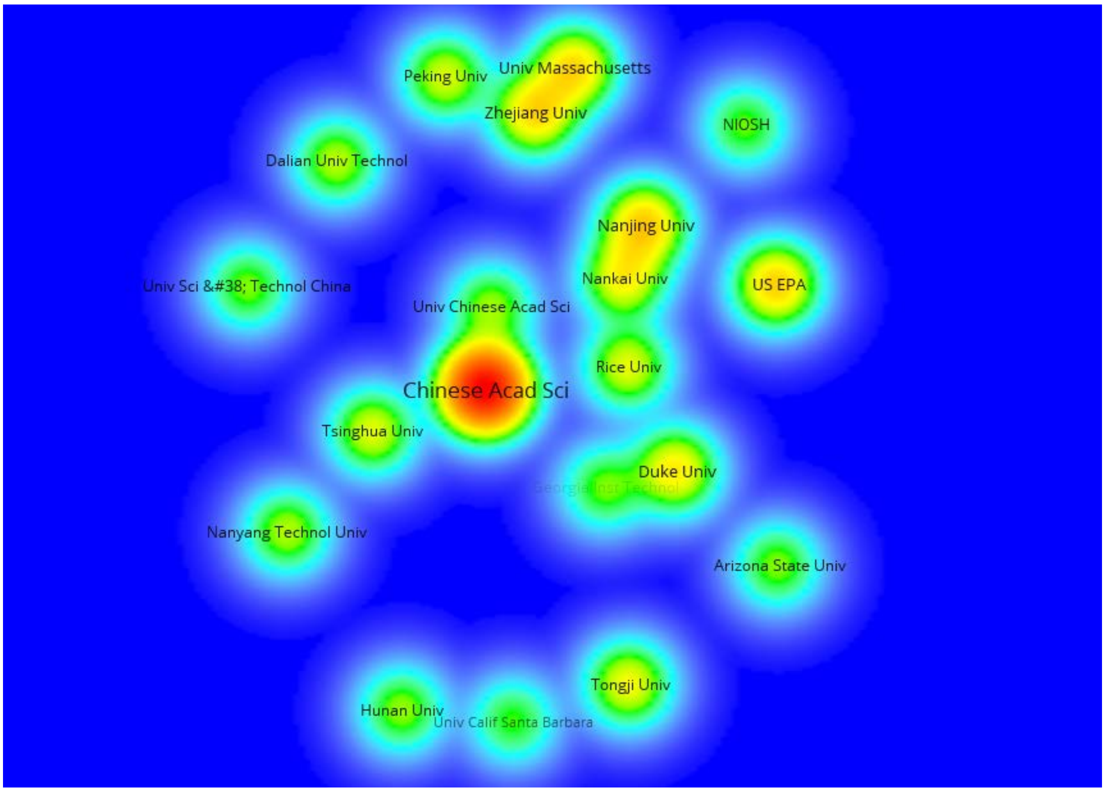

Fig. S3 High-impact institutions of density view by VOSviewer

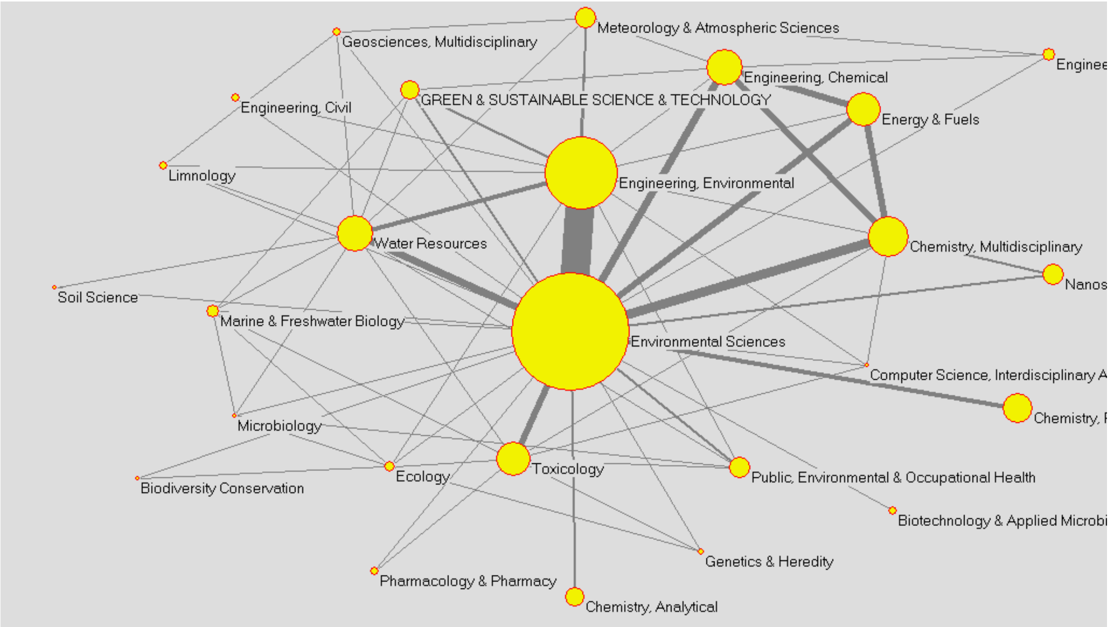

Fig. S4 Web of science categories co-occurrence clustering network by pajek

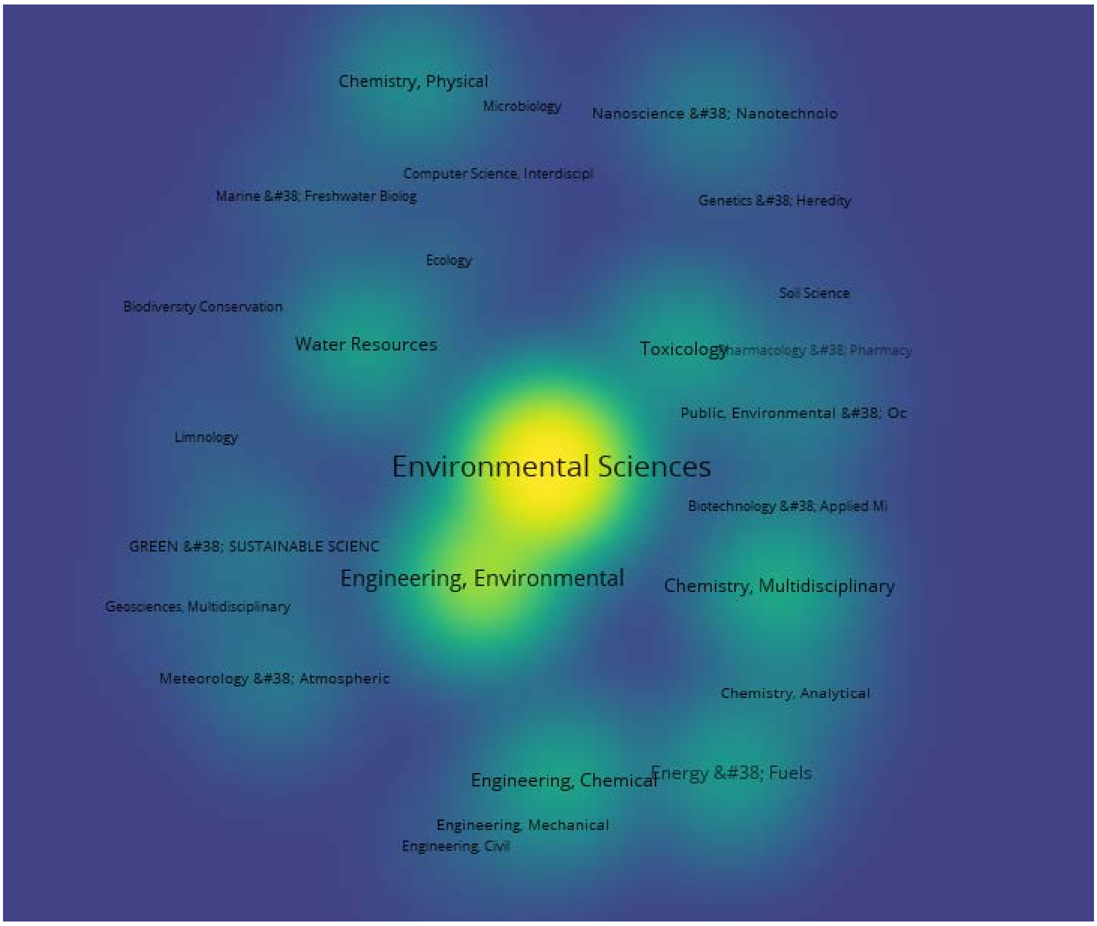

Fig. S5 Hot Web of science categories of density view by VOSviewer

Table.S1 Cluster summary of co-citation analysis by Citespace

ClusterID	Size	Silhouette	mean(Year)	Label (LSI)	Label (LLR)	Label (MI)
0	28	0.96	2006	adsorption; influence; single-walled carbon nanotubes; naphthalene sorption; cationic surfactant; conformation; multiwalled carbon nanotubes; presence; absence; organic matter | carbon nanotubes; natural organic matter; atrazine; modeling; thermodynamics; conformation; coagulation; surface-modified carbon nanotubes; determining environmental impacts; nanoparticles	carbon nanotube (285.98, 1.0E-4); multi-walled carbon nanotube (218.83, 1.0E-4); using carbon (150.1, 1.0E-4); nanoparticle-conjugated polymer nanocomposite (150.1, 1.0E-4); naphthalene sorption (137.56, 1.0E-4); cationic surfactant (137.56, 1.0E-4); carbon fiber (131.3, 1.0E-4); carbonaceous adsorbent (131.3, 1.0E-4); aromatic compound (131.3, 1.0E-4); surface-modified nanoscale carbon (118.77, 1.0E-4); electrolysis method (112.5, 1.0E-4); adsorption properties (112.5, 1.0E-4); trihalomethanes removal (106.24, 1.0E-4); drinking water source (106.24, 1.0E-4); or h2o2 (99.98, 1.0E-4); environmental fulvic acid (99.98, 1.0E-4); dynamic filtration (93.72, 1.0E-4); personal care product (93.72, 1.0E-4); reactive oxygen species production (87.46, 1.0E-4); photochemical behavior (87.46, 1.0E-4); aqueous solution (81.72, 1.0E-4); sorption behaviour (81.2, 1.0E-4); single wall carbon nanotube (74.95, 1.0E-4); nicotine adsorption (74.95, 1.0E-4); metal ion (68.98, 1.0E-4); natural organic matter removal (68.7, 1.0E-4); adsorption equilibrium (62.44, 1.0E-4); sulfur hexafluoride (62.44, 1.0E-4); carbon nanomaterial (61.28, 1.0E-4); silver nanoparticle (61.26, 1.0E-4); water purification (61.24, 1.0E-4); comparative study (59.48, 1.0E-4); pharmaceutical antibiotics (56.98, 1.0E-4); adsorption mechanism (56.19, 1.0E-4); different organic chemical (56.19, 1.0E-4); organic matter (51.42, 1.0E-4); effective removal (49.94, 1.0E-4); amino-functionalized magnetic nanoparticle (49.94, 1.0E-4); polycyclic aromatic hydrocarbon (46.33, 1.0E-4); physicochemical property (43.7, 1.0E-4); fulvic acid (43.03,	triazine-based pollutant (0.28); semi-analytical model (0.28); low-temperature wgs reaction (0.28); ni-ceo2 catalyst (0.28); bulk counterpart (0.28); plasma mass spectrometry (0.28); fullerene df-1 (0.28); rgo core-shell nanocomposite (0.28); ion release kinetics (0.28); promoter effect (0.28); respiratory deposition (0.28); biological tissue (0.28); graphene-supported ni (0.28); radiosensitive mammalian cell (0.28); nonmonotonic retention (0.28); phytoremediation system (0.28); combined effect (0.28); using single particle (0.28); polyvinylpyrrolidone-coated silver nanoparticle (0.28); one-step approach (0.28); nano-scale tio2 (0.28); organic carbon (0.25); silver sulfide nanomaterial (0.25); bacterial tactic response (0.25); amino modification (0.25); magna straus (0.25); physiochemical properties (0.25); electrochemical filter (0.25); siberian sturgeon (0.25); test media (0.25); nitrogen-fixing bacteria (0.25); potential toxicity (0.25); oxide-based nanomaterial (0.25); common aqueous antibiotic tetracycline (0.25); juvenile carp (0.25); cnt size (0.25); aquatic chemistry (0.25); methyl violet (0.23); bone marrow

					1.0E-4); graphene oxide (36.51, 1.0E-4); competitive adsorption (33.58, 1.0E-4); aqueous solution chemistry (32.78, 1.0E-4); nanosized alumina (31.2, 1.0E-4); carbonaceous nanoparticle (30.57, 1.0E-4); monoaromatic compound (30.17, 1.0E-4); multiwalled carbon nanotube (28.83, 1.0E-4); suwannee river (27.3, 1.0E-4); adsorbed phenanthrene (26.79, 1.0E-4); black carbon (26.79, 1.0E-4); microwave-induced carbon nanotube (24.96, 1.0E-4); surface-modified carbon nanotube (23.44, 1.0E-4); tio2 nanoparticle (22.88, 1.0E-4); single-walled carbon nanotube (21.1, 1.0E-4); available carbon nanotube (20.13, 1.0E-4); solution chemistry (20.08, 1.0E-4); methyl violet (18.72, 1.0E-4); synthetic organic chemical (18.07, 1.0E-4); background solution chemistry (18.07, 1.0E-4); engineered nanoparticle (16.51, 1.0E-4); wastewater treatment (14.87, 0.001); alpha-ethinyl estradiol (14.6, 0.001); adsorption kinetics (14.6, 0.001); zinc oxide nanoparticle (14.06, 0.001); inorganic nanoparticle (13.42, 0.001); titanium dioxide (12.69, 0.001); organic carbon (12.48, 0.001); titanium dioxide nanoparticle (10.16, 0.005); graphene oxide nanomaterial (9.33, 0.005); zno nanoparticle (8.88, 0.005); gold nanoparticle (8.88, 0.005); metal oxide nanoparticle (8.5, 0.005); natural water (8.24, 0.005); indoor corona device (7.81, 0.01); c-60 fullerene (7.43, 0.01); porous media (7.19, 0.01); ceo2 nanoparticle (6.88, 0.01); silver ion (6.7, 0.01); triazine-based pollutant (6.23, 0.05); coated silver nanoparticle (5.88, 0.05); earthworm eisenia fetida (5.88, 0.05); anaerobic digestion (5.7, 0.05); mesoporous carbon (5.62, 0.05); plant species (5.43, 0.05); theoretical studies (5.34, 0.05);	cell (0.23); colloidal sio2 (0.23); double-layer compression (0.23); illumination mode (0.23); sludge digestion (0.23); environmental effect (0.23); swiss-webster mice (0.23); size characterization (0.23); containing waste incineration residue (0.23); histopathological effect (0.23); bulk zno (0.23); titanium nitride nanotube (0.23); free-living nematode caenorhabditis elegan (0.23); polymer-stabilised nanoparticle (0.23); soil mixture (0.23); pilot wastewater treatment plant (0.23); charge neutralization (0.23); electrochemical capacitive energy storage (0.23); coaxial array (0.23); multi-angle light scattering (0.23); field-flow fractionation (0.23); asymmetrical flow (0.23); comparative phototoxicity (0.23); electrolyte species (0.23); commercial tio2 nanoparticle (0.23); explicit fate modelling (0.23); microwave-induced carbon nanotube (0.22); sorption behavior (0.21); natural soil system (0.21); polyaromatic hydrocarbon (0.21); characterization factor (0.21); seventeen subcontinental freshwater (0.21); field study (0.21); green algae (0.21); agcl nanoparticle (0.21); ceriodaphnia dubia (0.21); urban soil (0.21); using different

					cuo nanoparticle (5.25, 0.05); environmental risk assessment (5.07, 0.05); graphene oxide nanoparticle (4.89, 0.05); algal toxicity (4.89, 0.05); modeling technique (4.89, 0.05); chemical transformation (4.79, 0.05); danio rerio (4.71, 0.05); daphnia magna (4.64, 0.05); toxicity effect (4.62, 0.05); cucurbita pepo (4.62, 0.05); ionizable aromatic compound (4.55, 0.05); oxidized multiwalled carbon nanotube (4.55, 0.05); microbial communities (4.53, 0.05); aerosol exposure mode (4.43, 0.05)	reducing agent (0.21); dunaliella tertiolecta (0.21); mediating c-60 phototransformation (0.21); chlorella vulgaris (0.21); surface complexation modeling (0.21); screening evaluation (0.21); methodological consideration (0.21); sewer system (0.21); inhibitory effect (0.21); vitro test (0.21); tetrahymena thermophila (0.21); nanoscale metal oxide (0.21); synergistic toxic effect (0.21); reduced graphene oxide material (0.21); direct feeding (0.21); bacterial community structure (0.21); different release scenario (0.21); nanosized alumina (0.21); other natural adsorbent (0.2); wavelength dependency (0.2); sativus l (0.2); diesel soot (0.2); part 2-toxicity (0.2); product characterization (0.2); anatase nanoparticle (0.2)
1	25	0.974	2006	carbon nanotubes; effect; porous media; retention; bacteria; transport; nanoparticles; impacts; evidence; wastewater | single-walled carbon nanotubes; toxic effects; development; coli; rats; pulmonary toxicity; instilled multiwall carbon nanotubes; male fischer; bone marrow cells; determining environmental impacts	multi-wall carbon nanotube (149.21, 1.0E-4); new health risk (149.21, 1.0E-4); carbon nanotube (144.18, 1.0E-4); oxidation reaction (142.43, 1.0E-4); normal human embryonic lung cell (128.89, 1.0E-4); single-walled carbon nanotube (124.33, 1.0E-4); pulmonary toxicity (122.13, 1.0E-4); male fischer (122.13, 1.0E-4); instilled multiwall carbon nanotube (122.13, 1.0E-4); murine lung (115.38, 1.0E-4); human lung fibroblast (108.63, 1.0E-4); direct fibrogenic effect (108.63, 1.0E-4); sludge process (101.89, 1.0E-4); subchronic exposure (97.54, 1.0E-4); world trade center (95.16, 1.0E-4); lung disease (95.16, 1.0E-4); case	semi-analytical model (0.2); respiratory deposition (0.2); low-temperature wgs reaction (0.2); ni-ceo2 catalyst (0.2); bulk counterpart (0.2); plasma mass spectrometry (0.2); fullerene df-1 (0.2); rgo core-shell nanocomposite (0.2); ion release kinetics (0.2); promoter effect (0.2); biological tissue (0.2); graphene-supported ni (0.2); radiosensitive mammalian cell (0.2); nonmonotonic retention (0.2); phytoremediation system (0.2); triazine-

						report (95.16, 1.0E-4); dust sample (95.16, 1.0E-4); world trade center patient (95.16, 1.0E-4); human cell line (88.44, 1.0E-4); engineered carbon nanotube (88.44, 1.0E-4); vitro evaluation (88.44, 1.0E-4); intratracheal instillation study (81.74, 1.0E-4); epithelial barrier (75.04, 1.0E-4); air-pollution particle (75.04, 1.0E-4); lung alveolar (75.04, 1.0E-4); nanomaterial interaction (75.04, 1.0E-4); dialkyl phthalate ester (68.36, 1.0E-4); toxic effect (68.33, 1.0E-4); multiwall carbon nanotube (67.48, 1.0E-4); viral removal (61.49, 1.0E-4); single-walled carbon nanotube filter (61.49, 1.0E-4); removing bioaerosol (47.81, 1.0E-4); carbon nanotube filter (47.81, 1.0E-4); health effect (44.15, 1.0E-4); silver nanoparticle (43.65, 1.0E-4); new ingredient (40.98, 1.0E-4); aqueous solution (35.77, 1.0E-4); ovary cell (34.14, 1.0E-4); anatase titanium dioxide (34.14, 1.0E-4); graphene oxide (27.07, 1.0E-4); chronic exposure (25.9, 1.0E-4); multiwalled carbon nanotube (25.45, 1.0E-4); multi-walled carbon nanotube (22.43, 1.0E-4); environmental effect (20.48, 1.0E-4); consumer product (16.85, 1.0E-4); tio2 nanoparticle (15.64, 1.0E-4); cnt size (13.65, 0.001); engineered nanoparticle (12.24, 0.001); wastewater treatment (11.02, 0.001); zinc oxide nanoparticle (10.42, 0.005); c-60 derivative (9.97, 0.005); titanium dioxide (9.41, 0.005); engineered nanomaterial (8.62, 0.005); aquatic environment (7.38, 0.01); acute pulmonary toxicity (7.1, 0.01); fullerene nanoparticle (6.92, 0.01); graphene oxide nanomaterial (6.92, 0.01); semi-analytical model (6.82, 0.01); respiratory deposition (6.82, 0.01); comparative photoactivity (6.67, 0.01); antibacterial properties	based pollutant (0.2); combined effect (0.2); using single particle (0.2); polyvinylpyrrolidone-coated silver nanoparticle (0.2); one-step approach (0.2); nano-scale tio2 (0.2); cnt size (0.17); silver sulfide nanomaterial (0.17); bacterial tactic response (0.17); amino modification (0.17); magna straus (0.17); physiochemical properties (0.17); electrochemical filter (0.17); siberian sturgeon (0.17); test media (0.17); nitrogen-fixing bacteria (0.17); potential toxicity (0.17); oxide-based nanomaterial (0.17); common aqueous antibiotic tetracycline (0.17); juvenile carp (0.17); organic carbon (0.17); aquatic chemistry (0.17); environmental effect (0.16); bone marrow cell (0.16); swiss-webster mice (0.16); colloidal sio2 (0.16); double-layer compression (0.16); illumination mode (0.16); sludge digestion (0.16); size characterization (0.16); containing waste incineration residue (0.16); histopathological effect (0.16); bulk zno (0.16); titanium nitride nanotube (0.16); free-living nematode caenorhabditis elegan (0.16); polymer-stabilised nanoparticle (0.16); soil mixture (0.16); pilot wastewater treatment plant (0.16); charge neutralization

				5	(6.67, 0.01); potential new waste management paradigm (6.67, 0.01); zno nanoparticle (6.58, 0.05); gold nanoparticle (6.58, 0.05); non-covalent functionalization (6.28, 0.05); nanotube suspension (6.28, 0.05); epigenetic toxicity (6.28, 0.05); reactive oxygen production (6.27, 0.05); porous media (6.26, 0.05); aggregation kinetics (6.24, 0.05); cosmetic product (5.91, 0.05); solvent extraction technique (5.91, 0.05); quantitative determination (5.91, 0.05); fullerene detection (5.91, 0.05); delineating oxidative processes (5.61, 0.05); thf peroxide (5.61, 0.05); aqueous c-60 preparation (5.61, 0.05); high performance liquid chromatography (5.57, 0.05); metal oxide nanoparticle (5.36, 0.05); escherichia coli inactivation (5.26, 0.05); water purification (5.23, 0.05); ceo2 nanoparticle (5.1, 0.05); aqueous suspension (5.09, 0.05); organochlorine compound (5.05, 0.05); negligible depletion solid-phase microextraction (5.05, 0.05); silver ion (4.97, 0.05); water-stable c-60 cluster (4.71, 0.05); radical oxidation (4.46, 0.05); organic matter (4.36, 0.05); coated silver nanoparticle (4.36, 0.05); earthworm eisenia fetida (4.36, 0.05); natural organic matter (4.35, 0.05); anaerobic digestion (4.23, 0.05); electron reduction (4.23, 0.05); conventional drinking water treatment processes (4.18, 0.05); adverse effect (4.16, 0.05); metal ion (4.09, 0.05); plant species (4.03, 0.05); cell death (4.03, 0.05)	(0.16); electrochemical capacitive energy storage (0.16); coaxial array (0.16); multi-angle light scattering (0.16); methyl violet (0.16); field-flow fractionation (0.16); asymmetrical flow (0.16); comparative phototoxicity (0.16); electrolyte species (0.16); commercial tio2 nanoparticle (0.16); explicit fate modelling (0.16); mediating c-60 phototransformation (0.15); characterization factor (0.15); seventeen subcontinental freshwater (0.15); sorption behavior (0.15); field study (0.15); natural soil system (0.15); green algae (0.15); microwave-induced carbon nanotube (0.15); agcl nanoparticle (0.15); ceriodaphnia dubia (0.15); urban soil (0.15); using different reducing agent (0.15); dunaliella tertiolecta (0.15); chlorella vulgaris (0.15); surface complexation modeling (0.15); screening evaluation (0.15); methodological consideration (0.15); sewer system (0.15); inhibitory effect (0.15); vitro test (0.15); tetrahymena thermophila (0.15); nanoscale metal oxide (0.15); synergistic toxic effect (0.15); reduced graphene oxide material (0.15); polyaromatic hydrocarbon (0.15); direct feeding (0.15); bacterial community structure (0.15); different release scenario (0.15); ovary

						cell (0.14); anatase titanium dioxide (0.14); wavelength dependency (0.14); sativus l (0.14); diesel soot (0.14); part 2-toxicity (0.14); product characterization (0.14); anatase nanoparticle (0.14)
2	24	0.984	2013	engineered nanomaterials; coordinating modeling; life cycle assessment studies; experimental research; transformations; form; reactive porous media; nanomaterial environmental fate; environmental fate modeling; gold nanoparticles | nanoparticles; fate; case study; seventeen subcontinental freshwaters; characterization factors; copper nanoparticles; reactive porous media; nanomaterial environmental fate; environmental fate modeling; gold nanoparticles	subtle effect (167.39, 1.0E-4); alpha-ethynylestradiol adsorption (162.45, 1.0E-4); risk quantification (157.52, 1.0E-4); effect modeling (157.52, 1.0E-4); probabilistic exposure (157.52, 1.0E-4); surface water chemical parameter (152.59, 1.0E-4); influencing fate (152.59, 1.0E-4); colloid facilitated contaminant transport (152.59, 1.0E-4); using dynamic probabilistic modeling (142.72, 1.0E-4); changing world (142.72, 1.0E-4); future environmental emission (142.72, 1.0E-4); envisioning nano release dynamics (142.72, 1.0E-4); environmental exposure assessment (137.79, 1.0E-4); lake retention (132.86, 1.0E-4); natural clay colloid (127.93, 1.0E-4); modeling aggregation (123, 1.0E-4); regulatory oversight (118.07, 1.0E-4); nanomaterial life cycle (118.07, 1.0E-4); cerium oxide (114.26, 1.0E-4); wastewater-derived organic matter (113.14, 1.0E-4); hematite colloid (108.22, 1.0E-4); earthworm eisenia fetida (104.42, 1.0E-4); engineered nanomaterial (104.11, 1.0E-4); marine scallop chlamy (103.29, 1.0E-4); integrated biomarker approach (103.29, 1.0E-4); environmental relevant concentration (103.29, 1.0E-4); nanoparticulate hematite (93.44, 1.0E-4); nanosilver size (88.51, 1.0E-4); coating variation (88.51, 1.0E-4); using lumbriculus variegatus (88.51, 1.0E-4); waste management (78.67, 1.0E-4); use activities (78.67, 1.0E-4); environmental releases (78.67, 1.0E-4); manufactured	semi-analytical model (0.59); low-temperature wgs reaction (0.59); ni-ceo2 catalyst (0.59); bulk counterpart (0.59); plasma mass spectrometry (0.59); fullerene df-1 (0.59); rgo core-shell nanocomposite (0.59); ion release kinetics (0.59); promoter effect (0.59); respiratory deposition (0.59); biological tissue (0.59); graphene-supported ni (0.59); radiosensitive mammalian cell (0.59); nonmonotonic retention (0.59); phytoremediation system (0.59); triazine-based pollutant (0.59); combined effect (0.59); using single particle (0.59); polyvinylpyrrolidone-coated silver nanoparticle (0.59); one-step approach (0.59); nano-scale tio2 (0.59); silver sulfide nanomaterial (0.53); bacterial tactic response (0.53); amino modification (0.53); magna straus (0.53); physiochemical properties (0.53); electrochemical filter (0.53); siberian sturgeon (0.53); test media (0.53); nitrogen-fixing bacteria (0.53); potential toxicity (0.53); oxide-based nanomaterial (0.53); common aqueous

					nanoparticle (77.18, 1.0E-4); functional tio2 nanoparticle (73.75, 1.0E-4); aqueous environment (73.75, 1.0E-4); graphene oxide (72.45, 1.0E-4); citrate-stabilized gold nanoparticle (68.17, 1.0E-4); cerium translocation (63.9, 1.0E-4); oxide nanoparticle (63.9, 1.0E-4); kidney bean plant (63.9, 1.0E-4); physiological processes (63.9, 1.0E-4); porous media (60.28, 1.0E-4); reactive porous media (58.99, 1.0E-4); standardized abrasion condition (54.07, 1.0E-4); environmental release (54.07, 1.0E-4); commercial tile (54.07, 1.0E-4); molecular response (49.15, 1.0E-4); binary combination (49.15, 1.0E-4); perfluorooctane sulfonate (44.23, 1.0E-4); engineered nanoparticle (43.73, 1.0E-4); algal toxicity (42.32, 1.0E-4); inorganic nanoparticle (42.09, 1.0E-4); cerium oxide nanoparticle (39.31, 1.0E-4); pyrolyzed biomass (39.31, 1.0E-4); case study (35.64, 1.0E-4); caenorhabditis elegan (34.4, 1.0E-4); ceria nanoparticle (34.4, 1.0E-4); solution chemistry (32.04, 1.0E-4); freshwater ecotoxicity characterisation factor (29.48, 1.0E-4); zinc oxide nanoparticle (27.05, 1.0E-4); single walled carbon nanotube (26.73, 1.0E-4); aqueous solution (25.19, 1.0E-4); building material (24.56, 1.0E-4); single-walled carbon nanotube (23.12, 1.0E-4); carbon nanotube (22.43, 1.0E-4); characterization factor (19.65, 1.0E-4); seventeen subcontinental freshwater (19.65, 1.0E-4); daphnia magna (19.06, 1.0E-4); titanium dioxide nanoparticle (18.63, 1.0E-4); fullerene nanoparticle (18.52, 1.0E-4); graphene oxide nanomaterial (18.52, 1.0E-4); humic acid (17.75, 1.0E-4); titanium dioxide (14.95, 0.001); containing waste incineration residue (14.73, 0.001); cucurbita	antibiotic tetracycline (0.53); juvenile carp (0.53); cnt size (0.53); organic carbon (0.53); aquatic chemistry (0.53); containing waste incineration residue (0.5); bone marrow cell (0.5); colloidal sio2 (0.5); double-layer compression (0.5); illumination mode (0.5); sludge digestion (0.5); environmental effect (0.5); swiss-webster mice (0.5); size characterization (0.5); histopathological effect (0.5); bulk zno (0.5); titanium nitride nanotube (0.5); free-living nematode caenorhabditis elegan (0.5); polymer-stabilised nanoparticle (0.5); soil mixture (0.5); pilot wastewater treatment plant (0.5); charge neutralization (0.5); electrochemical capacitive energy storage (0.5); coaxial array (0.5); multi-angle light scattering (0.5); methyl violet (0.5); field-flow fractionation (0.5); asymmetrical flow (0.5); comparative phototoxicity (0.5); electrolyte species (0.5); commercial tio2 nanoparticle (0.5); explicit fate modelling (0.5); characterization factor (0.48); seventeen subcontinental freshwater (0.48); sorption behavior (0.48); field study (0.48); natural soil system (0.48); green algae (0.48); microwave-induced carbon nanotube (0.48); agcl nanoparticle (0.48); ceriodaphnia dubia (0.48); urban soil

					pepo (14.17, 0.001); water purification (14.02, 0.001); arabidopsis thaliana (13.73, 0.001); nanoparticle transport (12.69, 0.001); multiwalled carbon nanotube (12.24, 0.001); aquatic environment (11.95, 0.001); adverse effect (11.15, 0.001); metal ion (10.97, 0.001); theoretical studies (10.61, 0.005); carbon nanomaterial (10.43, 0.005); facile synthesis (10.07, 0.005); suwannee river (9.89, 0.005); graphene oxide nanoparticle (9.71, 0.005); comparative study (9.71, 0.005); modeling technique (9.71, 0.005); silica nanoparticle (9.53, 0.005); multi-walled carbon nanotube (9.15, 0.005); aerosol exposure mode (8.81, 0.005); nano-zno particle (8.63, 0.005); comparative eco-toxicities (8.63, 0.005); bacillus subtilis (8.45, 0.005); ionic strength (8.45, 0.005); relevant nanomaterial (8.27, 0.005); human health risk (8.27, 0.005); escherichia coli (8.27, 0.005)	(0.48); using different reducing agent (0.48); dunaliella tertiolecta (0.48); mediating c-60 phototransformation (0.48); chlorella vulgaris (0.48); surface complexation modeling (0.48); screening evaluation (0.48); methodological consideration (0.48); sewer system (0.48); inhibitory effect (0.48); vitro test (0.48); tetrahymena thermophila (0.48); nanoscale metal oxide (0.48); synergistic toxic effect (0.48); reduced graphene oxide material (0.48); polyaromatic hydrocarbon (0.48); direct feeding (0.48); bacterial community structure (0.48); different release scenario (0.48); building material (0.46); wavelength dependency (0.46); sativus l (0.46); diesel soot (0.46); part 2-toxicity (0.46); product characterization (0.46); anatase nanoparticle (0.46); tio2 nanomaterial (0.46)
3	23	0.964	2011	fate; silver; nanoparticles; sewerage networks; zinc; danish environment; modeling flows; concentrations; nanotextiles; health effects | silver nanoparticles; nanomaterials; toxicity; soils; full toxicity potential; earthworm eisenia fetida; bioavailability; short-term soil	environmental fate (149.31, 1.0E-4); stream dynamics (149.31, 1.0E-4); watershed-scale model (149.31, 1.0E-4); non-labile silver species (148.76, 1.0E-4); spatio-temporal approach (140.16, 1.0E-4); probabilistic modelling (140.16, 1.0E-4); engineered nanomaterial emission (140.16, 1.0E-4); land application (135.87, 1.0E-4); chemical transformation (132.28, 1.0E-4); diffusive gradient (131.58, 1.0E-4); thin film (131.58, 1.0E-4); x-ray absorption spectroscopy (131.58, 1.0E-4); wastewater treatment (129.72, 1.0E-4); silver nanoparticle sulfidation	semi-analytical model (0.72); low-temperature wgs reaction (0.72); ni-ceo2 catalyst (0.72); bulk counterpart (0.72); plasma mass spectrometry (0.72); fullerene df-1 (0.72); rgo core-shell nanocomposite (0.72); ion release kinetics (0.72); promoter effect (0.72); respiratory deposition (0.72); biological tissue (0.72); graphene-supported ni (0.72); radiosensitive mammalian cell (0.72); nonmonotonic retention

				bioassays; silver ions; program	(127.31, 1.0E-4); using various analytical technique (123.04, 1.0E-4); littoral lake mesocosm (123.04, 1.0E-4); life cycle assessment (119.6, 1.0E-4); environmental risk assessment (118.94, 1.0E-4); environmental life cycle assessment (118.79, 1.0E-4); nanosilver-enabled bandage (118.79, 1.0E-4); antibacterial product (114.55, 1.0E-4); rapid chromatographic separation (114.55, 1.0E-4); environmental water (114.55, 1.0E-4); silver-containing nanoparticle (114.55, 1.0E-4); dissoluble ag (114.55, 1.0E-4); nanomaterial risk forecasting (106.08, 1.0E-4); functional assay-based strategy (106.08, 1.0E-4); comparative toxicity potential (101.87, 1.0E-4); swiss waste incineration plant (101.08, 1.0E-4); nanometer range (101.08, 1.0E-4); chemical characterization (101.08, 1.0E-4); ash fraction (101.08, 1.0E-4); fly ashe (101.08, 1.0E-4); microplastic exposure assessment (96.83, 1.0E-4); short-term soil bioassay (92.59, 1.0E-4); full toxicity potential (92.59, 1.0E-4); engineered nanoparticle (89.93, 1.0E-4); comprehensive probabilistic modelling (88.37, 1.0E-4); environmental emission (88.37, 1.0E-4); nanoparticle environmental risk modeling (84.16, 1.0E-4); much ado (84.16, 1.0E-4); appropriate fate descriptor (84.16, 1.0E-4); mesophilic anaerobic digestion (83.38, 1.0E-4); short-term exposure (83.38, 1.0E-4); photoinduced transformation (79.13, 1.0E-4); silver sulfide nanoparticle (79.13, 1.0E-4); rethinking stability (79.13, 1.0E-4); silver standardized nanomaterial (74.89, 1.0E-4); estuarine bivalve scrobicularia plana (74.89, 1.0E-4); surface water (73.06, 1.0E-4); silver speciation (70.67, 1.0E-4); carbon nanotube (67.2, 1.0E-4); recreational lake (66.46, 1.0E-	(0.72); phytoremediation system (0.72); triazine-based pollutant (0.72); combined effect (0.72); using single particle (0.72); polyvinylpyrrolidone-coated silver nanoparticle (0.72); one-step approach (0.72); nano-scale tio2 (0.72); silver sulfide nanomaterial (0.65); nitrogen-fixing bacteria (0.65); bacterial tactic response (0.65); amino modification (0.65); magna straus (0.65); physiochemical properties (0.65); electrochemical filter (0.65); siberian sturgeon (0.65); test media (0.65); potential toxicity (0.65); oxide-based nanomaterial (0.65); common aqueous antibiotic tetracycline (0.65); juvenile carp (0.65); cnt size (0.65); organic carbon (0.65); aquatic chemistry (0.65); explicit fate modelling (0.61); bone marrow cell (0.61); colloidal sio2 (0.61); double-layer compression (0.61); illumination mode (0.61); sludge digestion (0.61); environmental effect (0.61); swiss-webster mice (0.61); size characterization (0.61); containing waste incineration residue (0.61); histopathological effect (0.61); bulk zno (0.61); titanium nitride nanotube (0.61); free-living nematode caenorhabditis elegan (0.61); polymer-stabilised nanoparticle

					4); old danube (66.46, 1.0E-4); one-year survey (66.46, 1.0E-4); silver nanoparticle synthesis route (62.28, 1.0E-4); comparative life cycle assessment (62.28, 1.0E-4); sequential studies (58.12, 1.0E-4); laundry detergent solution (58.12, 1.0E-4); silver nanoparticle (57.89, 1.0E-4); zno nanoparticle (56.98, 1.0E-4); environmental fate modeling (53.97, 1.0E-4); surface water system (52.98, 1.0E-4); natural organic matter (52.29, 1.0E-4); water chemistry (50.23, 1.0E-4); chemical composition (48.78, 1.0E-4); ecotoxicological test media (48.78, 1.0E-4); aggregation status (48.78, 1.0E-4); multi-walled carbon nanotube (47.74, 1.0E-4); ceo2 nanoparticle (46.24, 1.0E-4); methane production (45.17, 1.0E-4); physical characterization (44.61, 1.0E-4); porous media (42.52, 1.0E-4); synthetic seawater (41.12, 1.0E-4); natural freshwater (41.12, 1.0E-4); graphene oxide (40.15, 1.0E-4); engineered nanomaterial (38.71, 1.0E-4); solution chemistry (38.42, 1.0E-4); toxicity effect (38.13, 1.0E-4); earthworm eisenia fetida (37.42, 1.0E-4); phosphate buffer (36.55, 1.0E-4); species sensitivity distribution (36.36, 1.0E-4); single-walled carbon nanotube (34.78, 1.0E-4); silver ion (34.41, 1.0E-4); zinc oxide nanoparticle (33.99, 1.0E-4); nanofate model (32.29, 1.0E-4); life cycle assessment studies (31.98, 1.0E-4); aqueous solution (30.21, 1.0E-4); aquatic environment (29.8, 1.0E-4); aqueous media (29.17, 1.0E-4); sewerage network (27.41, 1.0E-4); divalent electrolyte (26.97, 1.0E-4); multiwalled carbon nanotube (24.57, 1.0E-4); citrate-coated silver nanoparticle (24.3, 1.0E-4); coordinating modeling (22.87, 1.0E-4); daphnia magna (22.86, 1.0E-4); indoor nanomaterial emission	(0.61); soil mixture (0.61); pilot wastewater treatment plant (0.61); charge neutralization (0.61); electrochemical capacitive energy storage (0.61); coaxial array (0.61); multi-angle light scattering (0.61); methyl violet (0.61); field-flow fractionation (0.61); asymmetrical flow (0.61); comparative phototoxicity (0.61); electrolyte species (0.61); commercial tio2 nanoparticle (0.61); field study (0.58); agcl nanoparticle (0.58); sewer system (0.58); characterization factor (0.58); seventeen subcontinental freshwater (0.58); sorption behavior (0.58); natural soil system (0.58); green algae (0.58); microwave-induced carbon nanotube (0.58); ceriodaphnia dubia (0.58); urban soil (0.58); using different reducing agent (0.58); dunaliella tertiolecta (0.58); mediating c-60 phototransformation (0.58); chlorella vulgaris (0.58); surface complexation modeling (0.58); screening evaluation (0.58); methodological consideration (0.58); inhibitory effect (0.58); vitro test (0.58); tetrahymena thermophila (0.58); nanoscale metal oxide (0.58); synergistic toxic effect (0.58); reduced graphene oxide material (0.58); polyaromatic hydrocarbon (0.58); direct feeding (0.58); bacterial community

					(22.84, 1.0E-4); human health characterization factor (22.84, 1.0E-4); fullerene nanoparticle (22.21, 1.0E-4); inorganic nanoparticle (19.98, 1.0E-4)	structure (0.58); different release scenario (0.58); indoor nanomaterial emission (0.56); human health characterization factor (0.56); wavelength dependency (0.56); sativus l (0.56); diesel soot (0.56); part 2-toxicity (0.56); product characterization (0.56); anatase nanoparticle (0.56)
4	23	0.951	2007	nanoparticles; impacts; wastewater sludge; evidence; wastewater; gills; toxicological response; mytilus galloprovincialis; cadmium; tio2 nanoparticles | effects; oxidative damage; bulk counterparts; oxidative stress; zebrafish; marine bivalve m; bulk zno; depuration; gills; human health	interactive effect (144.89, 1.0E-4); case studies (133.82, 1.0E-4); larval zebrafish (125.49, 1.0E-4); behavioral effect (125.49, 1.0E-4); c-60 nanoparticle exposure (119.04, 1.0E-4); lumbricus rubellus (119.04, 1.0E-4); population dynamics (119.04, 1.0E-4); other contaminant (112.61, 1.0E-4); physiological effect (106.18, 1.0E-4); rainbow trout hepatocyte (99.77, 1.0E-4); zns nanocrystal (93.37, 1.0E-4); photosynthetic pigment content (86.99, 1.0E-4); distilled water (80.63, 1.0E-4); particle dissolution (80.63, 1.0E-4); comparative toxicity (80.63, 1.0E-4); bulk zno suspension (80.63, 1.0E-4); danio rerio (79.79, 1.0E-4); aquatic organism (79.66, 1.0E-4); titanium dioxide (76.42, 1.0E-4); scenedesmus obliquus (75.82, 1.0E-4); acute toxicity (75.7, 1.0E-4); capping agent (74.28, 1.0E-4); human health (72.68, 1.0E-4); worsts case condition (67.96, 1.0E-4); conceptual modeling (67.96, 1.0E-4); using nzvi (67.96, 1.0E-4); mytilus galloprovincialis (66.06, 1.0E-4); toxicological response (66.06, 1.0E-4); available data (65.87, 1.0E-4); carbon nanomaterial fullerene (59.45, 1.0E-4); antioxidant responses (59.45, 1.0E-4); titanium dioxide nanoparticle (57.59, 1.0E-4); zebrafish embryo (52.83, 1.0E-4); metal ion (46.4,	bulk counterpart (0.22); nano-scale tio2 (0.22); semi-analytical model (0.22); low-temperature wgs reaction (0.22); ni-ceo2 catalyst (0.22); plasma mass spectrometry (0.22); fullerene df-1 (0.22); rgo core-shell nanocomposite (0.22); ion release kinetics (0.22); promoter effect (0.22); respiratory deposition (0.22); biological tissue (0.22); graphene-supported ni (0.22); radiosensitive mammalian cell (0.22); nonmonotonic retention (0.22); phytoremediation system (0.22); triazine-based pollutant (0.22); combined effect (0.22); using single particle (0.22); polyvinylpyrrolidone-coated silver nanoparticle (0.22); one-step approach (0.22); potential toxicity (0.2); juvenile carp (0.2); silver sulfide nanomaterial (0.2); bacterial tactic response (0.2); amino modification (0.2); magna straus (0.2); physiochemical properties (0.2); electrochemical filter (0.2); siberian sturgeon

					1.0E-4); nanoparticle characterisation (46.22, 1.0E-4); phagocytic activity (46.22, 1.0E-4); vitro exposure (46.22, 1.0E-4); silica nanoparticle (41.75, 1.0E-4); first exposure (39.62, 1.0E-4); embryonal development (39.62, 1.0E-4); mediterranean sea urchin (39.62, 1.0E-4); biomarker responses (33.01, 1.0E-4); marine bivalve m (33.01, 1.0E-4); graphene oxide (30.38, 1.0E-4); gene expression (28.81, 1.0E-4); ceriodaphnia dubia (26.4, 1.0E-4); synergistic toxic effect (26.4, 1.0E-4); environmental risk assessment (23.2, 1.0E-4); illumination mode (19.8, 1.0E-4); bulk zno (19.8, 1.0E-4); free-living nematode caenorhabditis elegan (19.8, 1.0E-4); comparative phototoxicity (19.8, 1.0E-4); multi-walled carbon nanotube (16.69, 1.0E-4); carbon nanotube (15.1, 0.001); potential toxicity (13.2, 0.001); juvenile carp (13.2, 0.001); wastewater treatment (12.37, 0.001); zinc oxide nanoparticle (11.7, 0.001); aqueous solution (10.56, 0.005); natural water (10.03, 0.005); engineered nanoparticle (8.04, 0.005); graphene oxide nanomaterial (7.76, 0.01); gold nanoparticle (7.38, 0.01); single-walled carbon nanotube (7.25, 0.01); bulk counterpart (6.6, 0.05); nano-scale tio2 (6.6, 0.05); water purification (5.87, 0.05); silver ion (5.57, 0.05); organic matter (4.89, 0.05); coated silver nanoparticle (4.89, 0.05); earthworm eisenia fetida (4.89, 0.05); solution chemistry (4.68, 0.05); plant species (4.52, 0.05); theoretical studies (4.44, 0.05); preparation method (4.39, 0.05); japanese medaka embryo (4.39, 0.05); fullerene water suspension (4.39, 0.05); multiwalled carbon nanotube (4.37, 0.05); carbon nanomaterial (4.37, 0.05); porous media (4.3, 0.05); suwannee river (4.14, 0.05);	(0.2); test media (0.2); nitrogen-fixing bacteria (0.2); oxide-based nanomaterial (0.2); common aqueous antibiotic tetracycline (0.2); cnt size (0.2); organic carbon (0.2); aquatic chemistry (0.2); illumination mode (0.18); bulk zno (0.18); free-living nematode caenorhabditis elegan (0.18); comparative phototoxicity (0.18); histopathological effect (0.18); bone marrow cell (0.18); colloidal sio2 (0.18); double-layer compression (0.18); sludge digestion (0.18); environmental effect (0.18); swiss-webster mice (0.18); size characterization (0.18); containing waste incineration residue (0.18); titanium nitride nanotube (0.18); polymer-stabilised nanoparticle (0.18); soil mixture (0.18); pilot wastewater treatment plant (0.18); charge neutralization (0.18); electrochemical capacitive energy storage (0.18); coaxial array (0.18); multi-angle light scattering (0.18); methyl violet (0.18); field-flow fractionation (0.18); asymmetrical flow (0.18); electrolyte species (0.18); commercial tio2 nanoparticle (0.18); explicit fate modelling (0.18); ceriodaphnia dubia (0.17); synergistic toxic effect (0.17); nanoscale metal oxide (0.17); characterization factor (0.17); seventeen subcontinental freshwater (0.17);

					graphene oxide nanoparticle (4.06, 0.05); comparative study (4.06, 0.05); modeling technique (4.06, 0.05); chemical transformation (3.99, 0.05); toxicity effect (3.84, 0.1); cucurbita pepo (3.84, 0.1); bacillus subtilis (3.54, 0.1); cerium uptake (3.46, 0.1); surface properties (3.46, 0.1); raphanus sativus (3.46, 0.1); escherichia coli (3.46, 0.1); citric acid (3.46, 0.1); commercial ceo2 nanoparticle (3.46, 0.1); developmental responses (3.39, 0.1); single walled carbon nanotube (3.39, 0.1); environmental fate (3.39, 0.1); stream dynamics (3.39, 0.1); watershed-scale model (3.39, 0.1); crop plant (3.39, 0.1)	sorption behavior (0.17); field study (0.17); natural soil system (0.17); green algae (0.17); microwave-induced carbon nanotube (0.17); agcl nanoparticle (0.17); urban soil (0.17); using different reducing agent (0.17); dunaliella tertiolecta (0.17); mediating c-60 phototransformation (0.17); chlorella vulgaris (0.17); surface complexation modeling (0.17); screening evaluation (0.17); methodological consideration (0.17); sewer system (0.17); inhibitory effect (0.17); vitro test (0.17); tetrahymena thermophila (0.17); reduced graphene oxide material (0.17); polyaromatic hydrocarbon (0.17); direct feeding (0.17); bacterial community structure (0.17); different release scenario (0.17); biomarker responses (0.16); marine bivalve m (0.16); marine invertebrate (0.16); bivalve mollusc scrobicularia plana (0.16); metal-based nanoparticle (0.16); annelid polychaete hediste diversicolor (0.16); wavelength dependency (0.16); sativus l (0.16)
	5	22	0.987	2008	aggregation; competing similarly-charged inorganic ions; charge behavior; nonmetallic nanoparticles; exposure modeling;	nanoparticle aggregation (189.78, 1.0E-4); understanding transport (114.14, 1.0E-4); natural aqueous matrice (107.63, 1.0E-4); silver nanoparticles suspension (101.14, 1.0E-4); electrolyte type (97.09, 1.0E-4); microstructural transformation	combined effect (0.21); semi-analytical model (0.21); low-temperature wgs reaction (0.21); ni-ceo2 catalyst (0.21); bulk counterpart (0.21); plasma mass spectrometry (0.21); fullerene df-1 (0.21);

				modeling; lagrangian approach; determining environmental impacts; nanoparticles; presence | role; zno nanoparticles; aggregation kinetics; morphology; stability; natural aqueous matrices; metal oxide nanoparticles; combined effects; heterotrophic wastewater biomass; biosorption	(93.67, 1.0E-4); haliotis diversicolor supertexta (92.97, 1.0E-4); lagrangian approach (86.96, 1.0E-4); early life stage (73.57, 1.0E-4); japanese medaka (73.57, 1.0E-4); titanium dioxide nanopowder (73.57, 1.0E-4); environmental condition (69.21, 1.0E-4); silicon nanoparticle (66.87, 1.0E-4); surface charge (66.58, 1.0E-4); ionic strength (57.43, 1.0E-4); aquatic organism (55.92, 1.0E-4); binary system (53.75, 1.0E-4); nanoparticle agglomeration (53.48, 1.0E-4); manufactured nanoparticle (51.39, 1.0E-4); common groundwater (46.79, 1.0E-4); zerovalent iron nanoparticle (46.79, 1.0E-4); marine environment (46.75, 1.0E-4); oryzias latipe (37.26, 1.0E-4); tio2 nanomaterial (33.41, 1.0E-4); full scale wastewater treatment plant (33.41, 1.0E-4); metal oxide nanoparticle (32.03, 1.0E-4); graphene oxide (29.15, 1.0E-4); zno nanoparticle (24.86, 1.0E-4); cuo nanoparticle (23.28, 1.0E-4); aqueous media (22.59, 1.0E-4); tio2 nanoparticle (20.81, 1.0E-4); suwannee river (20.59, 1.0E-4); double-layer compression (20.04, 1.0E-4); charge neutralization (20.04, 1.0E-4); commercial tio2 nanoparticle (20.04, 1.0E-4); fulvic acid (19.74, 1.0E-4); carbon nanotube (18.56, 1.0E-4); humic acid (17.33, 1.0E-4); comparative study (17.3, 1.0E-4); aqueous suspension (13.4, 0.001); engineered nanoparticle (13.19, 0.001); aqueous solution (10.13, 0.005); exposure modeling (9.82, 0.005); aggregation kinetics (8.88, 0.005); chemical transformation (8.11, 0.005); graphene oxide nanomaterial (7.45, 0.01); porous media (7.16, 0.01); multiwalled carbon nanotube (6.95, 0.01); combined effect (6.68, 0.01); multi-walled carbon nanotube (6.57, 0.05); c-	rgo core-shell nanocomposite (0.21); ion release kinetics (0.21); promoter effect (0.21); respiratory deposition (0.21); biological tissue (0.21); graphene-supported ni (0.21); radiosensitive mammalian cell (0.21); nonmonotonic retention (0.21); phytoremediation system (0.21); triazine-based pollutant (0.21); using single particle (0.21); polyvinylpyrrolidone-coated silver nanoparticle (0.21); one-step approach (0.21); nano-scale tio2 (0.21); silver sulfide nanomaterial (0.19); bacterial tactic response (0.19); amino modification (0.19); magna straus (0.19); physiochemical properties (0.19); electrochemical filter (0.19); siberian sturgeon (0.19); test media (0.19); nitrogen-fixing bacteria (0.19); potential toxicity (0.19); oxide-based nanomaterial (0.19); common aqueous antibiotic tetracycline (0.19); juvenile carp (0.19); cnt size (0.19); organic carbon (0.19); aquatic chemistry (0.19); double-layer compression (0.17); charge neutralization (0.17); commercial tio2 nanoparticle (0.17); bone marrow cell (0.17); colloidal sio2 (0.17); illumination mode (0.17); sludge digestion (0.17); environmental effect (0.17); swiss-webster mice (0.17); size

					60 fullerene (5.93, 0.05); water purification (5.64, 0.05); silver ion (5.35, 0.05); zinc oxide (4.89, 0.05); silver nanoparticle (4.72, 0.05); metal ion (4.41, 0.05); plant species (4.34, 0.05); theoretical studies (4.26, 0.05); carbon nanomaterial (4.19, 0.05); environmental risk assessment (4.05, 0.05); graphene oxide nanoparticle (3.9, 0.05); algal toxicity (3.9, 0.05); modeling technique (3.9, 0.05); toxicity effect (3.68, 0.1); cucurbita pepo (3.68, 0.1); wastewater treatment (3.61, 0.1); physico-chemical behavior (3.47, 0.1); water chemistry (3.47, 0.1); cerium uptake (3.32, 0.1); surface properties (3.32, 0.1); raphanus sativus (3.32, 0.1); citric acid (3.32, 0.1); commercial ceo2 nanoparticle (3.32, 0.1); vitro cytotoxicity (3.28, 0.1); using catfish (3.28, 0.1); developmental responses (3.25, 0.1); environmental fate (3.25, 0.1); stream dynamics (3.25, 0.1); watershed-scale model (3.25, 0.1); crop plant (3.25, 0.1); zinc oxide nanoparticle (3.18, 0.1); cerium oxide (3.18, 0.1); arabidopsis thaliana seedling (3.18, 0.1); methane production (3.18, 0.1); arabidopsis thaliana gene expression (3.11, 0.1); carbon dot (3.11, 0.1); non-labile silver species (3.11, 0.1); nutrient status (3.11, 0.1); human hepg2 cell (3.1, 0.1); using allium cepa (3.1, 0.1); ag nanoparticle exposure (3.03, 0.1); mesoporous carbon (3.03, 0.1); spatio-temporal approach (2.96, 0.1); dde bioaccumulation (2.96, 0.1); catalytic degradation (2.96, 0.1); probabilistic modelling (2.96, 0.1); glycine max (2.96, 0.1); zn ion exposure (2.96, 0.1); soil column (2.96, 0.1); engineered nanomaterial emission (2.96, 0.1)	characterization (0.17); containing waste incineration residue (0.17); histopathological effect (0.17); bulk zno (0.17); titanium nitride nanotube (0.17); free-living nematode caenorhabditis elegan (0.17); polymer-stabilised nanoparticle (0.17); soil mixture (0.17); pilot wastewater treatment plant (0.17); electrochemical capacitive energy storage (0.17); coaxial array (0.17); multi-angle light scattering (0.17); methyl violet (0.17); field-flow fractionation (0.17); asymmetrical flow (0.17); comparative phototoxicity (0.17); electrolyte species (0.17); explicit fate modelling (0.17); bacterial community structure (0.16); characterization factor (0.16); seventeen subcontinental freshwater (0.16); sorption behavior (0.16); field study (0.16); natural soil system (0.16); green algae (0.16); microwave-induced carbon nanotube (0.16); agcl nanoparticle (0.16); ceriodaphnia dubia (0.16); urban soil (0.16); using different reducing agent (0.16); dunaliella tertiolecta (0.16); mediating c-60 phototransformation (0.16); chlorella vulgaris (0.16); surface complexation modeling (0.16); screening evaluation (0.16); methodological consideration (0.16);

						sewer system (0.16); inhibitory effect (0.16); vitro test (0.16); tetrahymena thermophila (0.16); nanoscale metal oxide (0.16); synergistic toxic effect (0.16); reduced graphene oxide material (0.16); polyaromatic hydrocarbon (0.16); direct feeding (0.16); different release scenario (0.16); tio2 nanomaterial (0.16); full scale wastewater treatment plant (0.16); geochemical reactivity (0.16); single extraction method (0.16); human bioaccessibility (0.16); wavelength dependency (0.16); sativus l (0.16); diesel soot (0.16)
6	21	0.928	2006	nanoparticles; evidence; wastewater; impacts; wastewater sludge; product characterization; wavelength dependency; spectrometric detection; high-performance liquid chromatography; dispersion | transport; porous media; biofilm; influence; heterotrophic wastewater biomass; biosorption; single-walled carbon nanotubes; non-covalent functionalization; nanotube suspensions; dispersant	c-60 derivative (160.55, 1.0E-4); preparation method (106.82, 1.0E-4); japanese medaka embryo (106.82, 1.0E-4); fullerene water suspension (106.82, 1.0E-4); uv irradiation (103.2, 1.0E-4); aqueous fullerene (103.2, 1.0E-4); c-60 nanoparticle (102.15, 1.0E-4); aqueous nc (96.13, 1.0E-4); characterizing photochemical transformation (96.13, 1.0E-4); water-stable c-60 cluster (95.93, 1.0E-4); humic acid (94.31, 1.0E-4); kinetic measurement (92.71, 1.0E-4); radical oxidation (92.47, 1.0E-4); aggregation state (91.09, 1.0E-4); electron reduction (89.4, 1.0E-4); aqueous suspension (76.47, 1.0E-4); escherichia coli inactivation (73.93, 1.0E-4); solvent extraction technique (70.86, 1.0E-4); quantitative determination (70.86, 1.0E-4); relevant condition (69.75, 1.0E-4); high performance liquid chromatography (67.82, 1.0E-4); reactive oxygen production (64.65, 1.0E-4); cosmetic product (61.75, 1.0E-4); fullerene detection (61.75, 1.0E-4); aqueous system (59.12,	fullerene df-1 (0.22); radiosensitive mammalian cell (0.22); semi-analytical model (0.22); low-temperature wgs reaction (0.22); ni-ceo2 catalyst (0.22); bulk counterpart (0.22); plasma mass spectrometry (0.22); rgo core-shell nanocomposite (0.22); ion release kinetics (0.22); promoter effect (0.22); respiratory deposition (0.22); biological tissue (0.22); graphene-supported ni (0.22); nonmonotonic retention (0.22); phytoremediation system (0.22); triazine-based pollutant (0.22); combined effect (0.22); using single particle (0.22); polyvinylpyrrolidone-coated silver nanoparticle (0.22); one-step approach (0.22); nano-scale tio2 (0.22); silver sulfide nanomaterial (0.2);

				5	1.0E-4); potential new waste management paradigm (58.49, 1.0E-4); comparative photoactivity (49.73, 1.0E-4); antibacterial properties (49.73, 1.0E-4); acute pulmonary toxicity (43.81, 1.0E-4); coated silica surface (40.27, 1.0E-4); conventional drinking water treatment processes (34.32, 1.0E-4); silver nanoparticle (34.14, 1.0E-4); daphnia magna (31.67, 1.0E-4); organochlorine compound (30.84, 1.0E-4); negligible depletion solid-phase microextraction (30.84, 1.0E-4); delineating oxidative processes (24.85, 1.0E-4); thf peroxide (24.85, 1.0E-4); aqueous c-60 preparation (24.85, 1.0E-4); multiwall carbon nanotube (23.84, 1.0E-4); carbon nanotube (19.4, 1.0E-4); non-covalent functionalization (18.96, 1.0E-4); nanotube suspension (18.96, 1.0E-4); epigenetic toxicity (18.96, 1.0E-4); graphene oxide (18.21, 1.0E-4); mediating c-60 phototransformation (15.42, 1.0E-4); aqueous c-60 cluster (14.46, 0.001); engineered nanoparticle (13.65, 0.001); wastewater treatment (12.3, 0.001); oryzias latipe (12.23, 0.001); titanium dioxide (10.49, 0.005); aqueous solution (10.49, 0.005); tio2 nanoparticle (10.41, 0.005); c-60 fullerene (10.18, 0.005); bone marrow cell (9.48, 0.005); swiss-webster mice (9.48, 0.005); natural organic matter (8.22, 0.005); graphene oxide nanomaterial (7.71, 0.01); gold nanoparticle (7.34, 0.01); multi-walled carbon nanotube (7, 0.01); fullerene df-1 (6.61, 0.05); radiosensitive mammalian cell (6.61, 0.05); zinc oxide nanoparticle (6.25, 0.05); silver ion (5.54, 0.05); fullerene nanoparticle (5.16, 0.05); titanium dioxide nanoparticle (5.01, 0.05); transport behavior (4.99, 0.05); tube length (4.99, 0.05); water-saturated quartz sand (4.99,	bacterial tactic response (0.2); amino modification (0.2); magna straus (0.2); physiochemical properties (0.2); electrochemical filter (0.2); siberian sturgeon (0.2); test media (0.2); nitrogen-fixing bacteria (0.2); potential toxicity (0.2); oxide-based nanomaterial (0.2); common aqueous antibiotic tetracycline (0.2); juvenile carp (0.2); cnt size (0.2); organic carbon (0.2); aquatic chemistry (0.2); bone marrow cell (0.18); swiss-webster mice (0.18); colloidal sio2 (0.18); double-layer compression (0.18); illumination mode (0.18); sludge digestion (0.18); environmental effect (0.18); size characterization (0.18); containing waste incineration residue (0.18); histopathological effect (0.18); bulk zno (0.18); titanium nitride nanotube (0.18); free-living nematode caenorhabditis elegan (0.18); polymer-stabilised nanoparticle (0.18); soil mixture (0.18); pilot wastewater treatment plant (0.18); charge neutralization (0.18); electrochemical capacitive energy storage (0.18); coaxial array (0.18); multi-angle light scattering (0.18); methyl violet (0.18); field-flow fractionation (0.18); asymmetrical flow (0.18); comparative phototoxicity (0.18); electrolyte species

					0.05); organic matter (4.86, 0.05); coated silver nanoparticle (4.86, 0.05); functionalized multi-wall carbon nanotube (4.82, 0.05); anaerobic digestion (4.71, 0.05); vadose zone (4.66, 0.05); metal ion (4.56, 0.05); plant species (4.49, 0.05); theoretical studies (4.41, 0.05); cuo nanoparticle (4.34, 0.05); carbon nanomaterial (4.34, 0.05); porous media (4.2, 0.05); environmental risk assessment (4.19, 0.05); suwannee river (4.12, 0.05); graphene oxide nanoparticle (4.04, 0.05); algal toxicity (4.04, 0.05); modeling technique (4.04, 0.05); chemical transformation (3.97, 0.05); toxicity effect (3.81, 0.1); cucurbita pepo (3.81, 0.1); microbial communities (3.74, 0.1); bacillus subtilis (3.51, 0.1); ionic strength (3.51, 0.1); cerium uptake (3.44, 0.1); surface properties (3.44, 0.1); raphanus sativus (3.44, 0.1); escherichia coli (3.44, 0.1); citric acid (3.44, 0.1); commercial ceo2 nanoparticle (3.44, 0.1); developmental responses (3.37, 0.1); environmental fate (3.37, 0.1); stream dynamics (3.37, 0.1); watershed-scale model (3.37, 0.1)	(0.18); commercial tio2 nanoparticle (0.18); explicit fate modelling (0.18); mediating c-60 phototransformation (0.17); urban soil (0.17); screening evaluation (0.17); vitro test (0.17); nanoscale metal oxide (0.17); bacterial community structure (0.17); characterization factor (0.17); seventeen subcontinental freshwater (0.17); sorption behavior (0.17); field study (0.17); natural soil system (0.17); green algae (0.17); microwave-induced carbon nanotube (0.17); agcl nanoparticle (0.17); ceriodaphnia dubia (0.17); using different reducing agent (0.17); dunaliella tertiolecta (0.17); chlorella vulgaris (0.17); surface complexation modeling (0.17); methodological consideration (0.17); sewer system (0.17); inhibitory effect (0.17); tetrahymena thermophila (0.17); synergistic toxic effect (0.17); reduced graphene oxide material (0.17); polyaromatic hydrocarbon (0.17); direct feeding (0.17); different release scenario (0.17); wavelength dependency (0.16); sativus l (0.16); product characterization (0.16); geochemical reactivity (0.16); single extraction method (0.16); human bioaccessibility (0.16); diesel soot (0.16); part 2-toxicity (0.16)
7	20	0.932	2008	transport; single-	porous media (527.16, 1.0E-4);	low-temperature wgs

				walled carbon nanotubes; aggregation kinetics; low surfactant concentrations; low-temperature wgs reaction; fate; environment; evaluation; surface; ni-ceo2 catalyst | porous media; pore-scale investigation; scanning cytometry; using laser; nanoparticle transport; nanoparticles; retention; fullerene nanoparticles; role; carbonaceous nanoparticles	fullerene nanoparticle (369.1, 1.0E-4); vadose zone (151.73, 1.0E-4); transport behavior (149.87, 1.0E-4); tube length (149.87, 1.0E-4); water-saturated quartz sand (149.87, 1.0E-4); functionalized multi-wall carbon nanotube (146.74, 1.0E-4); natural soil (143.05, 1.0E-4); solution chemistry (141.59, 1.0E-4); deposition kinetics (132.96, 1.0E-4); aqueous fullerene nanoparticle (132.96, 1.0E-4); organic macromolecule (131.25, 1.0E-4); stable carbon nanoparticle (129.72, 1.0E-4); empirical model (127.93, 1.0E-4); downhole hydrocarbon detection (126.18, 1.0E-4); sandy soil (124.6, 1.0E-4); using laser (123.25, 1.0E-4); controlling factor (121.12, 1.0E-4); scanning cytometry (119.49, 1.0E-4); pore-scale investigation (119.49, 1.0E-4); synthesis method (114.4, 1.0E-4); water-saturated sand column (111.8, 1.0E-4); functionalized carbon nanotube (104.25, 1.0E-4); silica nanoparticle (100.54, 1.0E-4); low surfactant concentration (99.2, 1.0E-4); photoactivity properties (92.49, 1.0E-4); ag-graphene oxide nanocomposite (89.15, 1.0E-4); nanoparticle transport (88.43, 1.0E-4); stabilizing agent (87.41, 1.0E-4); enhanced mobility (84.15, 1.0E-4); columbia ecotype (79.18, 1.0E-4); cell suspension (79.18, 1.0E-4); silver nanoparticle (78.4, 1.0E-4); single walled carbon nanotube (77.9, 1.0E-4); clean porous media (74.22, 1.0E-4); multi-walled carbon nanotube deposition (74.22, 1.0E-4); graphene-based composite (69.3, 1.0E-4); new perspective (67.29, 1.0E-4); nanomaterial aquatic ecotoxicity (64.4, 1.0E-4); carbon nanotoube (64.4, 1.0E-4); particle size (62.15, 1.0E-4); bridging complexation (59.52, 1.0E-4); spectrometric detection (58.97, 1.0E-4); uv-vis	reaction (0.44); ni-ceo2 catalyst (0.44); promoter effect (0.44); graphene-supported ni (0.44); semi-analytical model (0.44); bulk counterpart (0.44); plasma mass spectrometry (0.44); fullerene df-1 (0.44); rgo core-shell nanocomposite (0.44); ion release kinetics (0.44); respiratory deposition (0.44); biological tissue (0.44); radiosensitive mammalian cell (0.44); nonmonotonic retention (0.44); phytoremediation system (0.44); triazine-based pollutant (0.44); combined effect (0.44); using single particle (0.44); polyvinylpyrrolidone-coated silver nanoparticle (0.44); one-step approach (0.44); nano-scale tio2 (0.44); aquatic chemistry (0.4); silver sulfide nanomaterial (0.4); bacterial tactic response (0.4); amino modification (0.4); magna straus (0.4); physiochemical properties (0.4); electrochemical filter (0.4); siberian sturgeon (0.4); test media (0.4); nitrogen-fixing bacteria (0.4); potential toxicity (0.4); oxide-based nanomaterial (0.4); common aqueous antibiotic tetracycline (0.4); juvenile carp (0.4); cnt size (0.4); organic carbon (0.4); electrolyte species (0.37); bone marrow cell (0.37); colloidal sio2 (0.37); double-layer compression

						spectroscopic (58.97, 1.0E-4); fullerene aggregate nc (58.97, 1.0E-4); high-performance liquid chromatography (58.97, 1.0E-4); solid phase coating (58.14, 1.0E-4); using near-infrared fluorescence spectroscopy (52.88, 1.0E-4); quantitative analysis (52.88, 1.0E-4); alkaline protease (47.64, 1.0E-4); efficient catalyst (47.64, 1.0E-4); waste-activated sludge (47.64, 1.0E-4); surfactant-modified fullerene nanoparticle (42.41, 1.0E-4); 5-polychlorinated biphenyl (42.41, 1.0E-4); one-step synthesis (37.21, 1.0E-4); aerosol spray pyrolysis (37.21, 1.0E-4); electrocatalytic activity (37.21, 1.0E-4); nanoparticles-laden graphene crumple (37.21, 1.0E-4); metal oxide nanoparticle (36.28, 1.0E-4); unsaturated porous media (36.22, 1.0E-4); surface-modified nanoparticle (36.21, 1.0E-4); cell death (31.94, 1.0E-4); carbon nanotube (30.32, 1.0E-4); aggregation kinetics (29.06, 1.0E-4); tio2 nanoparticle (27.88, 1.0E-4); facile synthesis (27.09, 1.0E-4); sulfide-containing aqueous solution (26.91, 1.0E-4); oxide-facilitated reduction (26.91, 1.0E-4); engineered nanomaterial (23.6, 1.0E-4); single-walled carbon nanotube (23, 1.0E-4); aquatic environment (22.72, 1.0E-4); wastewater treatment (22.7, 1.0E-4); cdte nanoparticle (21.82, 1.0E-4); titanium dioxide (19.38, 1.0E-4); aqueous solution (19.38, 1.0E-4); arabidopsis thaliana (18.46, 1.0E-4); engineered nanoparticle (18.45, 1.0E-4); water purification (17.73, 1.0E-4); wavelength dependency (16.8, 1.0E-4); product characterization (16.8, 1.0E-4); electrolyte species (16.25, 1.0E-4); zinc oxide nanoparticle (15.02, 0.001); graphene oxide nanomaterial (14.25, 0.001); zno	(0.37); illumination mode (0.37); sludge digestion (0.37); environmental effect (0.37); swiss-webster mice (0.37); size characterization (0.37); containing waste incineration residue (0.37); histopathological effect (0.37); bulk zno (0.37); titanium nitride nanotube (0.37); free-living nematode caenorhabditis elegan (0.37); polymer-stabilised nanoparticle (0.37); soil mixture (0.37); pilot wastewater treatment plant (0.37); charge neutralization (0.37); electrochemical capacitive energy storage (0.37); coaxial array (0.37); multi-angle light scattering (0.37); methyl violet (0.37); field-flow fractionation (0.37); asymmetrical flow (0.37); comparative phototoxicity (0.37); commercial tio2 nanoparticle (0.37); explicit fate modelling (0.37); different release scenario (0.35); characterization factor (0.35); seventeen subcontinental freshwater (0.35); sorption behavior (0.35); field study (0.35); natural soil system (0.35); green algae (0.35); microwave-induced carbon nanotube (0.35); agcl nanoparticle (0.35); ceriodaphnia dubia (0.35); urban soil (0.35); using different reducing agent (0.35); dunaliella tertiolecta (0.35); mediating c-60 phototransformation

					nanoparticle (13.55, 0.001); gold nanoparticle (13.55, 0.001); natural water (12.27, 0.001); c-60 fullerene (11.34, 0.001); enhanced transport (11.16, 0.001); aquatic chemistry (10.83, 0.001); ceo2 nanoparticle (10.51, 0.005); silver ion (10.23, 0.005); inorganic nanoparticle (10.16, 0.005); humic acid (10.06, 0.005); aqueous c-60 cluster (9.99, 0.005); organic matter (8.99, 0.005); coated silver nanoparticle (8.99, 0.005); earthworm eisenia fetida (8.99, 0.005); anaerobic digestion (8.71, 0.005); metal ion (8.43, 0.005); plant species (8.29, 0.005)	(0.35); chlorella vulgaris (0.35); surface complexation modeling (0.35); screening evaluation (0.35); methodological consideration (0.35); sewer system (0.35); inhibitory effect (0.35); vitro test (0.35); tetrahymena thermophila (0.35); nanoscale metal oxide (0.35); synergistic toxic effect (0.35); reduced graphene oxide material (0.35); polyaromatic hydrocarbon (0.35); direct feeding (0.35); bacterial community structure (0.35); wavelength dependency (0.34); product characterization (0.34); collector surface (0.34); aggregated nanoparticle (0.34); sativus l (0.34); diesel soot (0.34); part 2-toxicity (0.34); anatase nanoparticle (0.34)
8	20	0.957	2009	effect; evaluation; nano; test medium; cuo-exposed daphnia magna; txrf; total cu body burden; spectroscopy study; bacteria-nanoparticle interactions; environmental implications | effects; freshwater snail bellamya aeruginosa; cu bioaccumulation; sediment-associated cuo nanoparticles; oxidative stress responses; practical considerations; exposure system; anabaena variabilis;	toxicological risk (160.4, 1.0E-4); using gene expression (155.35, 1.0E-4); oxide-coated textile (151.61, 1.0E-4); copper toxicity (150.43, 1.0E-4); ecotoxicological investigation (150.14, 1.0E-4); soil nematode (150.14, 1.0E-4); green microalgae (147.07, 1.0E-4); nanoecotoxicity testing (147.07, 1.0E-4); oxidative stress responses (146.65, 1.0E-4); tio2 nanoparticle aggregate (146.18, 1.0E-4); by-design approach (145.53, 1.0E-4); airborne nanoparticle release (145.53, 1.0E-4); physicochemical metrics (141.69, 1.0E-4); freshwater snail bellamya aeruginosa (140.63, 1.0E-4); cu bioaccumulation (140.63, 1.0E-4); sulfidation kinetics (140.31, 1.0E-4); studying cellular uptake (136.74, 1.0E-4); agglomeration properties (135.73, 1.0E-4); bacterial	semi-analytical model (0.56); low-temperature wgs reaction (0.56); ni-ceo2 catalyst (0.56); bulk counterpart (0.56); plasma mass spectrometry (0.56); fullerene df-1 (0.56); rgo core-shell nanocomposite (0.56); ion release kinetics (0.56); promoter effect (0.56); respiratory deposition (0.56); biological tissue (0.56); graphene-supported ni (0.56); radiosensitive mammalian cell (0.56); nonmonotonic retention (0.56); phytoremediation system (0.56); triazine-based pollutant (0.56); combined effect (0.56); using single particle (0.56);

				microbial communities; multimethod approach	cytotoxicity (135.73, 1.0E-4); practical consideration (135.29, 1.0E-4); copper oxide nanoparticle (131.78, 1.0E-4); cultured fish hepatocyte (130.84, 1.0E-4); physical effect (130.27, 1.0E-4); conducting ecotoxicity test method (126.83, 1.0E-4); manufactured nanomaterial (126.83, 1.0E-4); toxicological effect (125.25, 1.0E-4); algae phaeodactylum tricornutum (121.89, 1.0E-4); test guideline (120.23, 1.0E-4); unicellular green algae (120.23, 1.0E-4); nanometer titanium dioxide (116.95, 1.0E-4); chlamydomonas reinhardtii (116.95, 1.0E-4); nanosized particle (115.21, 1.0E-4); exposure system (112.01, 1.0E-4); staphylococcus aureus atcc (110.2, 1.0E-4); enzyme toxicity (107.07, 1.0E-4); surface water sample (105.18, 1.0E-4); physicochemical transformation (105.18, 1.0E-4); aeruginosa atcc (102.14, 1.0E-4); oxidative damage (96.77, 1.0E-4); adverse effect (96.06, 1.0E-4); tio2 nanoparticle (95.8, 1.0E-4); particle-size effect (95.15, 1.0E-4); daphnia magna (92.98, 1.0E-4); engineered fe2o3 nanoparticle (92.3, 1.0E-4); co2-induced seawater acidification (92.3, 1.0E-4); test medium (90.13, 1.0E-4); total cu body burden (90.13, 1.0E-4); spectroscopy study (90.13, 1.0E-4); platinum nanoparticle (85.12, 1.0E-4); cuo-exposed daphnia magna (82.47, 1.0E-4); ceo2 nanoparticle (78.88, 1.0E-4); investigating algal toxicity (77.57, 1.0E-4); organic matter (77.14, 1.0E-4); marine environment (75.44, 1.0E-4); protozoan tetrahymena thermophila (75.09, 1.0E-4); toxic action (75.09, 1.0E-4); phenotypic event (75.09, 1.0E-4); hemocyte parameter (72.67, 1.0E-4); green-lipped mussel perna (72.67, 1.0E-4); cytometric analysis (72.67, 1.0E-4); graphene oxide (69.14,	polyvinylpyrrolidone-coated silver nanoparticle (0.56); one-step approach (0.56); nano-scale tio2 (0.56); siberian sturgeon (0.51); silver sulfide nanomaterial (0.51); bacterial tactic response (0.51); amino modification (0.51); magna straus (0.51); physiochemical properties (0.51); electrochemical filter (0.51); test media (0.51); nitrogen-fixing bacteria (0.51); potential toxicity (0.51); oxide-based nanomaterial (0.51); common aqueous antibiotic tetracycline (0.51); juvenile carp (0.51); cnt size (0.51); organic carbon (0.51); aquatic chemistry (0.51); histopathological effect (0.47); bone marrow cell (0.47); colloidal sio2 (0.47); double-layer compression (0.47); illumination mode (0.47); sludge digestion (0.47); environmental effect (0.47); swiss-webster mice (0.47); size characterization (0.47); containing waste incineration residue (0.47); bulk zno (0.47); titanium nitride nanotube (0.47); free-living nematode caenorhabditis elegan (0.47); polymer-stabilised nanoparticle (0.47); soil mixture (0.47); pilot wastewater treatment plant (0.47); charge neutralization (0.47); electrochemical capacitive energy storage (0.47); coaxial array (0.47); multi-

					1.0E-4); oreochromis niloticus (65.07, 1.0E-4); tissue accumulation (65.07, 1.0E-4); serum biochemistry (65.07, 1.0E-4); zinc nanoparticle (65.07, 1.0E-4); containing nanoscale polymeric complex (62.91, 1.0E-4); applying multi-marker approach (62.91, 1.0E-4); developmental toxicity (58.12, 1.0E-4); histopathological change (58.04, 1.0E-4); porous media (57.52, 1.0E-4); stable aqueous fullerene nanocrystal (55.05, 1.0E-4); freshwater alga euglena intermedia (53.18, 1.0E-4); bioaccumulation kinetics (53.18, 1.0E-4); carbon nanotube (52.04, 1.0E-4); hop frequency (48.34, 1.0E-4); different condition (48.34, 1.0E-4); humic acid (47.31, 1.0E-4); gene expression (44.87, 1.0E-4); agronomically-relevant rhizobium-legume symbiosis (43.52, 1.0E-4); possible mechanism (40.03, 1.0E-4); sub-toxic effect (40.03, 1.0E-4); environmental element (38.71, 1.0E-4); multi-walled carbon nanotube (37.99, 1.0E-4); methane production (36.49, 1.0E-4); algal toxicity (36.27, 1.0E-4); receiving manufactured nanomaterial (35.02, 1.0E-4); wastewater treatment plant (35.02, 1.0E-4); cu ion (33.92, 1.0E-4); hematite nanoparticle (31.45, 1.0E-4); solution chemistry (30.58, 1.0E-4); engineered nanomaterial (30.56, 1.0E-4); subcellular distribution (30.02, 1.0E-4); transfer efficiency (30.02, 1.0E-4); low nanomaterial concentration (29.17, 1.0E-4); wastewater treatment (28.17, 1.0E-4); single-walled carbon nanotube (25.73, 1.0E-4); reductive dechlorinating microbial communities (25.01, 1.0E-4); new zero-valent iron nanomaterial (25.01, 1.0E-4); titanium dioxide (24.04, 1.0E-4); aqueous solution (24.04, 1.0E-4)	angle light scattering (0.47); methyl violet (0.47); field-flow fractionation (0.47); asymmetrical flow (0.47); comparative phototoxicity (0.47); electrolyte species (0.47); commercial tio2 nanoparticle (0.47); explicit fate modelling (0.47); methodological consideration (0.45); characterization factor (0.45); seventeen subcontinental freshwater (0.45); sorption behavior (0.45); field study (0.45); natural soil system (0.45); green algae (0.45); microwave-induced carbon nanotube (0.45); agcl nanoparticle (0.45); ceriodaphnia dubia (0.45); urban soil (0.45); using different reducing agent (0.45); dunaliella tertiolecta (0.45); mediating c-60 phototransformation (0.45); chlorella vulgaris (0.45); surface complexation modeling (0.45); screening evaluation (0.45); sewer system (0.45); inhibitory effect (0.45); vitro test (0.45); tetrahymena thermophila (0.45); nanoscale metal oxide (0.45); synergistic toxic effect (0.45); reduced graphene oxide material (0.45); polyaromatic hydrocarbon (0.45); direct feeding (0.45); bacterial community structure (0.45); different release scenario (0.45); reductive dechlorinating microbial communities (0.43); new zero-valent iron nanomaterial

						(0.43); marine invertebrate (0.43); bivalve mollusc scrobicularia plana (0.43); metal-based nanoparticle (0.43); annelid polychaete hediste diversicolor (0.43); wavelength dependency (0.43); sativus l (0.43)
9	19	0.984	2008	influence; toxicity; multiwalled carbon nanotubes; natural organic matter; sediment dwelling; multiwall carbon nanotubes; carbon nanotube; gold nanoparticles; release; depuration | carbon nanotubes; sediment dwelling; sublethal toxicity; nano-titanium dioxide; marine polychaete; release; abrasion process; epoxy-based nanocomposite; direct feeding; trophic transfer	surface oxygen (143.37, 1.0E-4); occupational exposure (137.65, 1.0E-4); sublethal toxicity (135.13, 1.0E-4); environmental laboratory studies (134.63, 1.0E-4); engineered carbon-based nanomaterial (134.63, 1.0E-4); nano-titanium dioxide (131.94, 1.0E-4); marine polychaete (131.94, 1.0E-4); carbon nanotube type (126.25, 1.0E-4); long-term colloidal stability (126.25, 1.0E-4); stabilizing multi-walled carbon nanotube suspension (123.65, 1.0E-4); triton x-series surfactant (123.65, 1.0E-4); soil mineral (121.19, 1.0E-4); 14-labeled multi-walled carbon nanotube (117.92, 1.0E-4); benthic organism (109.67, 1.0E-4); marine food chain (109.67, 1.0E-4); lumbriculus variegatus (105.24, 1.0E-4); different stabilities (103.94, 1.0E-4); fresh surface water sample (103.94, 1.0E-4); quantitative investigation (103.6, 1.0E-4); carbon nanotube (102.74, 1.0E-4); multiwalled carbon nanotube (101.72, 1.0E-4); carbon nanotubes toxicity (99.37, 1.0E-4); natural organic matter (95.54, 1.0E-4); peat soil (91.72, 1.0E-4); suspending multi-walled carbon nanotube (91.72, 1.0E-4); earthworm bioaccumulation (90.66, 1.0E-4); containing model solid (78.63, 1.0E-4); 14-labeled multiwalled carbon nanotube (78.63, 1.0E-4); phase distribution (78.63, 1.0E-4); earthworm eisenia veneta (72.63, 1.0E-4); comparative	plasma mass spectrometry (0.29); biological tissue (0.29); using single particle (0.29); semi-analytical model (0.29); low-temperature wgs reaction (0.29); ni-ceo2 catalyst (0.29); bulk counterpart (0.29); fullerene df-1 (0.29); rgo core-shell nanocomposite (0.29); ion release kinetics (0.29); promoter effect (0.29); respiratory deposition (0.29); graphene-supported ni (0.29); radiosensitive mammalian cell (0.29); nonmonotonic retention (0.29); phytoremediation system (0.29); triazine-based pollutant (0.29); combined effect (0.29); polyvinylpyrrolidone-coated silver nanoparticle (0.29); one-step approach (0.29); nano-scale tio2 (0.29); electrochemical filter (0.26); common aqueous antibiotic tetracycline (0.26); silver sulfide nanomaterial (0.26); bacterial tactic response (0.26); amino modification (0.26); magna straus (0.26); physiochemical properties (0.26); siberian sturgeon (0.26); test media (0.26); nitrogen-fixing

					chronic toxicity (72.63, 1.0E-4); soil matrix (72.63, 1.0E-4); ionic zinc (72.63, 1.0E-4); surface modification (66.64, 1.0E-4); single-walled carbon nanotube retention (66.64, 1.0E-4); radio-labeled graphene (60.67, 1.0E-4); biological uptake (60.67, 1.0E-4); daphnia magna (59.42, 1.0E-4); multi-walled carbon nanotube (59.22, 1.0E-4); humic acid (55.34, 1.0E-4); natural organic matter type (54.71, 1.0E-4); octanol-water distribution measurement (48.77, 1.0E-4); potential ecological uptake (48.77, 1.0E-4); adverse effect (46.78, 1.0E-4); aqueous system (40.76, 1.0E-4); single-walled carbon nanotube (40.73, 1.0E-4); unsaturated porous media (39.04, 1.0E-4); graphene oxide (38.67, 1.0E-4); representative mature leachate (36.97, 1.0E-4); single-walled carbon nanotube behavior (36.97, 1.0E-4); epoxy-based nanocomposite (36.77, 1.0E-4); abrasion process (36.77, 1.0E-4); silver nanoparticle (36.77, 1.0E-4); porous media (32.18, 1.0E-4); diesel soot (30.64, 1.0E-4); hydrophobic organic contaminant (30.64, 1.0E-4); benthic invertebrate (30.64, 1.0E-4); tetrahymena thermophila (24.51, 1.0E-4); direct feeding (24.51, 1.0E-4); citrate-stabilized gold nanoparticle (23.02, 1.0E-4); size characterization (18.38, 1.0E-4); multi-angle light scattering (18.38, 1.0E-4); field-flow fractionation (18.38, 1.0E-4); asymmetrical flow (18.38, 1.0E-4); acute toxicity (17.45, 1.0E-4); wastewater treatment (15.75, 1.0E-4); titanium dioxide (13.45, 0.001); aqueous solution (13.45, 0.001); aquatic environment (13.34, 0.001); tio2 nanoparticle (12.86, 0.001); electrochemical filter (12.25, 0.001); common aqueous antibiotic tetracycline (12.25, 0.001); engineered nanomaterial	bacteria (0.26); potential toxicity (0.26); oxide-based nanomaterial (0.26); juvenile carp (0.26); cnt size (0.26); organic carbon (0.26); aquatic chemistry (0.26); size characterization (0.24); multi-angle light scattering (0.24); field-flow fractionation (0.24); asymmetrical flow (0.24); bone marrow cell (0.24); colloidal sio2 (0.24); double-layer compression (0.24); illumination mode (0.24); sludge digestion (0.24); environmental effect (0.24); swiss-webster mice (0.24); containing waste incineration residue (0.24); histopathological effect (0.24); bulk zno (0.24); titanium nitride nanotube (0.24); free-living nematode caenorhabditis elegan (0.24); polymer-stabilised nanoparticle (0.24); soil mixture (0.24); pilot wastewater treatment plant (0.24); charge neutralization (0.24); electrochemical capacitive energy storage (0.24); coaxial array (0.24); methyl violet (0.24); comparative phototoxicity (0.24); electrolyte species (0.24); commercial tio2 nanoparticle (0.24); explicit fate modelling (0.24); tetrahymena thermophila (0.23); direct feeding (0.23); methodological consideration (0.23); characterization factor (0.23); seventeen subcontinental

					(11.58, 0.001); titanium dioxide nanoparticle (11.02, 0.001); solution chemistry (11.02, 0.001); fullerene nanoparticle (9.88, 0.005); graphene oxide nanomaterial (9.88, 0.005); zno nanoparticle (9.4, 0.005); aggregation kinetics (8.92, 0.005); engineered nanoparticle (7.73, 0.01); water purification (7.48, 0.01); ceo2 nanoparticle (7.29, 0.01); natural water (7.26, 0.01); organic matter (6.23, 0.05); coated silver nanoparticle (6.23, 0.05); earthworm eisenia fetida (6.23, 0.05); plasma mass spectrometry (6.12, 0.05); biological tissue (6.12, 0.05); using single particle (6.12, 0.05); anaerobic digestion (6.04, 0.05); metal oxide nanoparticle (5.9, 0.05); zinc oxide nanoparticle (5.77, 0.05); theoretical studies (5.66, 0.05); carbon nanomaterial (5.56, 0.05); environmental risk assessment (5.37, 0.05); facile synthesis (5.37, 0.05); suwannee river (5.27, 0.05); graphene oxide nanoparticle (5.18, 0.05); comparative study (5.18, 0.05); algal toxicity (5.18, 0.05)	freshwater (0.23); sorption behavior (0.23); field study (0.23); natural soil system (0.23); green algae (0.23); microwave-induced carbon nanotube (0.23); agcl nanoparticle (0.23); ceriodaphnia dubia (0.23); urban soil (0.23); using different reducing agent (0.23); dunaliella tertiolecta (0.23); mediating c-60 phototransformation (0.23); chlorella vulgaris (0.23); surface complexation modeling (0.23); screening evaluation (0.23); sewer system (0.23); inhibitory effect (0.23); vitro test (0.23); nanoscale metal oxide (0.23); synergistic toxic effect (0.23); reduced graphene oxide material (0.23); polyaromatic hydrocarbon (0.23); bacterial community structure (0.23); different release scenario (0.23); diesel soot (0.22); hydrophobic organic contaminant (0.22); benthic invertebrate (0.22); anatase nanoparticle (0.22); pure anatase (0.22); wheat seedling (0.22); wavelength dependency (0.22); sativus l (0.22)
10	19	0.985	2010	graphene oxide; removal; role; oxide-based nanomaterials; aqueous media; pharmaceuticals; phenols; oxide-magnetic nanoparticles; phenols sensor; high-efficiency | graphene; exafs; modeling	modeling technique (293.96, 1.0E-4); graphene nanosheet (216.35, 1.0E-4); graphene-templated formation (206.02, 1.0E-4); preparing high-rate supercapacitor electrode (200.85, 1.0E-4); graphene oxide sheet (200.85, 1.0E-4); high-performance asymmetric supercapacitor (195.69, 1.0E-4); graphene-based electrode (195.69, 1.0E-4); metal oxide composite (190.52, 1.0E-4);	rgo core-shell nanocomposite (0.52); one-step approach (0.52); semi-analytical model (0.52); low-temperature wgs reaction (0.52); ni-ceo2 catalyst (0.52); bulk counterpart (0.52); plasma mass spectrometry (0.52); fullerene df-1 (0.52); ion release kinetics

				techniques; oxide-based composites; mechanistic insights; decontamination; phenols; graphene oxide; oxide-magnetic nanoparticles; phenols sensor	reduced graphene (190.52, 1.0E-4); potential electrode material (185.36, 1.0E-4); efficient electrocatalyst (180.2, 1.0E-4); zn-air batteries (180.2, 1.0E-4); ionic liquid (180.2, 1.0E-4); anchoring manganese oxide nanoparticle (180.2, 1.0E-4); catalytic degradation (179.84, 1.0E-4); controlled electronic structure (175.04, 1.0E-4); non-covalent doping (175.04, 1.0E-4); graphitic carbon nitride polymer (175.04, 1.0E-4); enhanced optoelectronic conversion (175.04, 1.0E-4); mechanistic insight (169.88, 1.0E-4); oxide-based composite (169.88, 1.0E-4); mesoporous carbon (166.46, 1.0E-4); facile preparation route (164.71, 1.0E-4); boron-doped graphene (164.71, 1.0E-4); cdte solar cell application (164.71, 1.0E-4); linear free energy relationship modeling (159.56, 1.0E-4); unveiling adsorption mechanism (159.56, 1.0E-4); functional theory computation (159.56, 1.0E-4); high oxygen-reduction activity (154.4, 1.0E-4); nitrogen-doped graphene (154.4, 1.0E-4); enhanced supercapacitor performance (154.4, 1.0E-4); flexible energy storage device (149.24, 1.0E-4); graphene paper (149.24, 1.0E-4); aqueous solution (145.89, 1.0E-4); colloidal graphene oxide nanoparticle (144.08, 1.0E-4); organic pollutant (140.23, 1.0E-4); metal oxide surface (138.92, 1.0E-4); ciprofloxacin adsorption (133.77, 1.0E-4); methylene blue (128.61, 1.0E-4); magnetite composite (128.61, 1.0E-4); silver nanoparticle (123.93, 1.0E-4); mechanical investigation (123.46, 1.0E-4); single-crystal zno (118.31, 1.0E-4); nanoporous metal oxide shell composite (118.31, 1.0E-4); controllable electrochemical synthesis (118.31, 1.0E-4); facile synthesis (116.05, 1.0E-4); emerging contaminant	(0.52); promoter effect (0.52); respiratory deposition (0.52); biological tissue (0.52); graphene-supported ni (0.52); radiosensitive mammalian cell (0.52); nonmonotonic retention (0.52); phytoremediation system (0.52); triazine-based pollutant (0.52); combined effect (0.52); using single particle (0.52); polyvinylpyrrolidone-coated silver nanoparticle (0.52); nano-scale tio2 (0.52); amino modification (0.47); oxide-based nanomaterial (0.47); silver sulfide nanomaterial (0.47); bacterial tactic response (0.47); magna straus (0.47); physiochemical properties (0.47); electrochemical filter (0.47); siberian sturgeon (0.47); test media (0.47); nitrogen-fixing bacteria (0.47); potential toxicity (0.47); common aqueous antibiotic tetracycline (0.47); juvenile carp (0.47); cnt size (0.47); organic carbon (0.47); aquatic chemistry (0.47); titanium nitride nanotube (0.44); electrochemical capacitive energy storage (0.44); coaxial array (0.44); colloidal sio2 (0.44); bone marrow cell (0.44); double-layer compression (0.44); illumination mode (0.44); sludge digestion (0.44); environmental effect (0.44); swiss-webster mice (0.44); size characterization (0.44); containing waste

					metformin (113.15, 1.0E-4); using graphene oxide (113.15, 1.0E-4); carboxylic group (108, 1.0E-4); electrochemical capacitor (108, 1.0E-4); carbon nanofiber hybrid (102.85, 1.0E-4); effective enrichment (102.85, 1.0E-4); plasma-facilitated synthesis (102.85, 1.0E-4); enhanced transport (101.86, 1.0E-4); graphene nanomaterial (97.7, 1.0E-4); tricresyl phosphate (97.7, 1.0E-4); ionic condensation (92.55, 1.0E-4); polarizable energy-storage membrane (92.55, 1.0E-4); high performance (92.55, 1.0E-4); soil trench (87.4, 1.0E-4); water purification (85.77, 1.0E-4); carbon nanomaterial (79.74, 1.0E-4); oxide-wrapped carbon (77.11, 1.0E-4); phenols sensor (77.11, 1.0E-4); graphene nanoribbon (71.96, 1.0E-4); graphene oxide nanosheet (66.82, 1.0E-4); engineered nanomaterial (62.05, 1.0E-4); theoretical investigation (61.67, 1.0E-4); magnetic graphene oxide (61.67, 1.0E-4); reduced graphene oxide (61.67, 1.0E-4); clofibric acid (56.53, 1.0E-4); tio2 nanoparticle (53.16, 1.0E-4); stereoselective adsorption behavior (46.24, 1.0E-4); isomeric pesticide (46.24, 1.0E-4); oxide-magnetic nanoparticle (46.24, 1.0E-4); efficient adsorbent (46.24, 1.0E-4); c composite (41.1, 1.0E-4); new synthesis (41.1, 1.0E-4); graphene oxide nanomaterial (40.1, 1.0E-4); humic acid (36.3, 1.0E-4); comprising carbon sphere (35.96, 1.0E-4); self-assembled hierarchical nanostructure (35.96, 1.0E-4); multi-walled carbon nanotube (35.43, 1.0E-4); aquatic environment (33.08, 1.0E-4); polycyclic aromatic hydrocarbon (32.84, 1.0E-4); graphene sheet (30.82, 1.0E-4); using cobalt (30.82, 1.0E-4); porous media (30.51, 1.0E-4); engineered nanoparticle (29.16, 1.0E-4); titanium dioxide	incineration residue (0.44); histopathological effect (0.44); bulk zno (0.44); free-living nematode caenorhabditis elegan (0.44); polymer-stabilised nanoparticle (0.44); soil mixture (0.44); pilot wastewater treatment plant (0.44); charge neutralization (0.44); multi-angle light scattering (0.44); methyl violet (0.44); field-flow fractionation (0.44); asymmetrical flow (0.44); comparative phototoxicity (0.44); electrolyte species (0.44); commercial tio2 nanoparticle (0.44); explicit fate modelling (0.44); surface complexation modeling (0.42); using different reducing agent (0.42); reduced graphene oxide material (0.42); characterization factor (0.42); seventeen subcontinental freshwater (0.42); sorption behavior (0.42); field study (0.42); natural soil system (0.42); green algae (0.42); microwave-induced carbon nanotube (0.42); agcl nanoparticle (0.42); ceriodaphnia dubia (0.42); urban soil (0.42); dunaliella tertiolecta (0.42); mediating c-60 phototransformation (0.42); chlorella vulgaris (0.42); screening evaluation (0.42); methodological consideration (0.42); sewer system (0.42); inhibitory effect (0.42); vitro test (0.42); tetrahymena

					nanoparticle (28.52, 1.0E-4); multiwalled carbon nanotube (27.55, 1.0E-4); wastewater treatment (26.27, 1.0E-4); double hydroxide (25.68, 1.0E-4); coagulation behavior (25.68, 1.0E-4); theoretical calculation study (25.68, 1.0E-4); metal oxide nanoparticle (25.14, 1.0E-4); zinc oxide nanoparticle (24.82, 1.0E-4); inorganic nanoparticle (23.7, 1.0E-4); graphene oxide (22.46, 1.0E-4); natural water (21.29, 1.0E-4)	thermophila (0.42); nanoscale metal oxide (0.42); synergistic toxic effect (0.42); polyaromatic hydrocarbon (0.42); direct feeding (0.42); bacterial community structure (0.42); different release scenario (0.42); double hydroxide (0.4); coagulation behavior (0.4); theoretical calculation study (0.4); wavelength dependency (0.4); sativus l (0.4); diesel soot (0.4); part 2-toxicity (0.4); product characterization (0.4)
11	19	0.982	2009	silver nanoparticles; inhibitory effects; dunaliella tertiolecta; chlorella vulgaris; nanomaterial environmental fate; modeling; gold nanoparticles; short-term effect; dependent ecotoxicity; bacterial toxicity | toxicity; overproduction; extracellular polymeric substances; enhanced resistance; nanomaterial environmental fate; modeling; gold nanoparticles; short-term effect; dependent ecotoxicity; bacterial toxicity	silver nanoparticle (586.08, 1.0E-4); coated silver nanoparticle (254.41, 1.0E-4); natural water (201.32, 1.0E-4); escherichia coli (180.97, 1.0E-4); bacterial activity (162.87, 1.0E-4); moderate silver concentration (158.15, 1.0E-4); wastewater treatment (154.88, 1.0E-4); cerium dioxide (153.44, 1.0E-4); detecting nanoparticulate silver (148.74, 1.0E-4); plasma-mass spectrometry (148.74, 1.0E-4); nanomaterial environmental fate (141.28, 1.0E-4); unique environmental risk (141.12, 1.0E-4); divalent electrolyte solution (140.94, 1.0E-4); modeling nanomaterial fate (139.37, 1.0E-4); research strategy (136.09, 1.0E-4); novel nanohybrid (136.09, 1.0E-4); aquatic system (133.48, 1.0E-4); particle properties (131.24, 1.0E-4); microbial communities (130.53, 1.0E-4); dissolution rate (126.39, 1.0E-4); ecotoxicology media (121.55, 1.0E-4); physical morphology (116.72, 1.0E-4); bacterial toxicity (116.72, 1.0E-4); typical nanomaterial (116.61, 1.0E-4); agrobacterium tumefacien (111.9, 1.0E-4); using sophora flavescens nanoparticle (111.73, 1.0E-4);	ion release kinetics (0.56); semi-analytical model (0.56); low-temperature wgs reaction (0.56); ni-ceo2 catalyst (0.56); bulk counterpart (0.56); plasma mass spectrometry (0.56); fullerene df-1 (0.56); rgo core-shell nanocomposite (0.56); promoter effect (0.56); respiratory deposition (0.56); biological tissue (0.56); graphene-supported ni (0.56); radiosensitive mammalian cell (0.56); nonmonotonic retention (0.56); phytoremediation system (0.56); triazine-based pollutant (0.56); combined effect (0.56); using single particle (0.56); polyvinylpyrrolidone-coated silver nanoparticle (0.56); one-step approach (0.56); nano-scale tio2 (0.56); bacterial tactic response (0.5); silver sulfide nanomaterial (0.5); amino modification (0.5);

					anaerobic digestion (111.43, 1.0E-4); humid airflow (107.09, 1.0E-4); short-term effect (107.09, 1.0E-4); nh4 reduction (102.28, 1.0E-4); nano-magnesium oxide particle (97.49, 1.0E-4); manufactured ag nanoparticle (92.71, 1.0E-4); long-term transformation (92.71, 1.0E-4); modeling nanosilver transformation (87.94, 1.0E-4); aquatic microcosm (84.21, 1.0E-4); ag nanoparticle (84.21, 1.0E-4); abiotic interaction (84.21, 1.0E-4); effective effluent treatment (83.18, 1.0E-4); enhanced resistance (78.43, 1.0E-4); extracellular polymeric substance (78.43, 1.0E-4); gold nanoparticle (75.86, 1.0E-4); suwannee river (75.2, 1.0E-4); natural organic matter alter (73.7, 1.0E-4); ionic strength (70.59, 1.0E-4); prokaryotic system (68.99, 1.0E-4); mass concentration (68.99, 1.0E-4); total surface area (68.99, 1.0E-4); graphene oxide (68.57, 1.0E-4); bacillus subtilis (65.49, 1.0E-4); toxicity effect (60.61, 1.0E-4); carbon nanotube (59.97, 1.0E-4); anti-bacterial performance (59.62, 1.0E-4); natural water condition (59.62, 1.0E-4); different polymer (59.62, 1.0E-4); porous media (57.05, 1.0E-4); bacterial fouling (54.97, 1.0E-4); microfiltration membrane (54.97, 1.0E-4); silver nanoparticle modification (54.97, 1.0E-4); aggregation kinetics (52.98, 1.0E-4); environmental transformation (50.35, 1.0E-4); situ study (50.35, 1.0E-4); zerovalent iron (49.11, 1.0E-4); adsorbed polymer (45.75, 1.0E-4); nano scale (45.75, 1.0E-4); nonsize measurement (44.4, 1.0E-4); titanium dioxide (43.19, 1.0E-4); nanoparticulate ceo2 (39.73, 1.0E-4); physico-chemical behaviour (39.73, 1.0E-4); multi-walled carbon nanotube (37.68, 1.0E-4); tio2	magna straus (0.5); physiochemical properties (0.5); electrochemical filter (0.5); siberian sturgeon (0.5); test media (0.5); nitrogen-fixing bacteria (0.5); potential toxicity (0.5); oxide-based nanomaterial (0.5); common aqueous antibiotic tetracycline (0.5); juvenile carp (0.5); cnt size (0.5); organic carbon (0.5); aquatic chemistry (0.5); sludge digestion (0.47); polymer-stabilised nanoparticle (0.47); soil mixture (0.47); pilot wastewater treatment plant (0.47); bone marrow cell (0.47); colloidal sio2 (0.47); double-layer compression (0.47); illumination mode (0.47); environmental effect (0.47); swiss-webster mice (0.47); size characterization (0.47); containing waste incineration residue (0.47); histopathological effect (0.47); bulk zno (0.47); titanium nitride nanotube (0.47); free-living nematode caenorhabditis elegan (0.47); charge neutralization (0.47); electrochemical capacitive energy storage (0.47); coaxial array (0.47); multi-angle light scattering (0.47); methyl violet (0.47); field-flow fractionation (0.47); asymmetrical flow (0.47); comparative phototoxicity (0.47); electrolyte species (0.47); commercial tio2 nanoparticle (0.47); explicit fate modelling

				5	nanoparticle (37.26, 1.0E-4); microbial community structure (35.09, 1.0E-4); comparative study (32.85, 1.0E-4); single-walled carbon nanotube (30.68, 1.0E-4); bacterial inactivation (30.49, 1.0E-4); aquatic media (30.49, 1.0E-4); specific ion (30.49, 1.0E-4); silver nanoparticle stability (30.49, 1.0E-4); aquatic environment (30.08, 1.0E-4); dependent ecotoxicity (25.94, 1.0E-4); organo-coated silver nanoparticle (25.94, 1.0E-4); observed toxicity (25.94, 1.0E-4); part 2-toxicity (25.09, 1.0E-4); natural organic matter (23.89, 1.0E-4); aqueous solution (23.84, 1.0E-4); danio rerio (23.79, 1.0E-4); solution chemistry (23.27, 1.0E-4); metallic nanoparticle (21.46, 1.0E-4); cuo sulfidation yield (21.46, 1.0E-4); green algae (20.07, 1.0E-4); dunaliella tertiolecta (20.07, 1.0E-4); chlorella vulgaris (20.07, 1.0E-4); inhibitory effect (20.07, 1.0E-4); ag speciation (19.85, 1.0E-4); engineered nanoparticle (19.1, 1.0E-4); inorganic nanomaterial (18.53, 1.0E-4); titanium dioxide nanoparticle (18.5, 1.0E-4); daphnia magna (18.04, 1.0E-4); multiwalled carbon nanotube (17.61, 1.0E-4); fullerene nanoparticle (17.53, 1.0E-4); graphene oxide nanomaterial (17.53, 1.0E-4)	(0.47); green algae (0.45); dunaliella tertiolecta (0.45); chlorella vulgaris (0.45); inhibitory effect (0.45); characterization factor (0.45); seventeen subcontinental freshwater (0.45); sorption behavior (0.45); field study (0.45); natural soil system (0.45); microwave-induced carbon nanotube (0.45); agcl nanoparticle (0.45); ceriodaphnia dubia (0.45); urban soil (0.45); using different reducing agent (0.45); mediating c-60 phototransformation (0.45); surface complexation modeling (0.45); screening evaluation (0.45); methodological consideration (0.45); sewer system (0.45); vitro test (0.45); tetrahymena thermophila (0.45); nanoscale metal oxide (0.45); synergistic toxic effect (0.45); reduced graphene oxide material (0.45); polyaromatic hydrocarbon (0.45); direct feeding (0.45); bacterial community structure (0.45); different release scenario (0.45); part 2-toxicity (0.43); wavelength dependency (0.43); sativus l (0.43); diesel soot (0.43); product characterization (0.43); anatase nanoparticle (0.43); tio2 nanomaterial (0.43); geochemical reactivity (0.43)
12	17	0.968	2009	engineered nanomaterials; environmental exposure;	eisenia fetida (162.47, 1.0E-4); engineered nanomaterial (142.46, 1.0E-4); probabilistic material flow analysis (98.97,	semi-analytical model (0.4); low-temperature wgs reaction (0.4); ni-ceo2 catalyst (0.4); bulk

				modeling; limitations; probabilistic material flow analysis; possibilities; paint; characterization; water; materials | fate; effects; eisenia fetida; nano aluminum oxide; primary wastewater treatment; pvp-capped silver nanoparticles; exploring controls; particle size; exposure modeling; biodistribution	1.0E-4); source water quality (98.35, 1.0E-4); multiwall carbon nanotube coagulation (98.35, 1.0E-4); nanowaste detection (97.45, 1.0E-4); heterotrophic wastewater biomass (96.59, 1.0E-4); environmental exposure (93.46, 1.0E-4); irish surface (93.16, 1.0E-4); regulatory system (93.16, 1.0E-4); nano-scale pollutant (93.16, 1.0E-4); drinking water (93.16, 1.0E-4); nanocopper removal (92.85, 1.0E-4); municipal wastewater (92.85, 1.0E-4); chemical speciation (89.76, 1.0E-4); physicochemical interaction (88.53, 1.0E-4); ecological perspective (85.35, 1.0E-4); freshwater food chain (82.39, 1.0E-4); copper nanoparticle (80.5, 1.0E-4); anabaena variabilis (78.01, 1.0E-4); intracellular nitrogen storage (78.01, 1.0E-4); nitrogen fixation rate (78.01, 1.0E-4); titanium dioxide nanomaterial (78.01, 1.0E-4); trophic transfer (77, 1.0E-4); rainbow trout cell line (70.7, 1.0E-4); comparative cytotoxicity study (70.7, 1.0E-4); nano zinc-oxide (66.41, 1.0E-4); nano silver (66.41, 1.0E-4); temporal resolution (66.41, 1.0E-4); exploring control (64.77, 1.0E-4); pvp-capped silver nanoparticle (64.77, 1.0E-4); high-throughput screening (63.12, 1.0E-4); environmental factor (62.11, 1.0E-4); cerium dioxide nanoparticle translocation (61.81, 1.0E-4); maize plant (61.81, 1.0E-4); low biosorption (57.5, 1.0E-4); coated engineered magnetic nanoparticle (57.5, 1.0E-4); granular sludge (57.5, 1.0E-4); magnetic susceptibility (57.5, 1.0E-4); biological response (53.25, 1.0E-4); nano-scale titanium dioxide (53.25, 1.0E-4); particle dose (53.25, 1.0E-4); graphene oxide (50.73, 1.0E-4); analytical measurement (49.05, 1.0E-4); prognostic risk	counterpart (0.4); plasma mass spectrometry (0.4); fullerene df-1 (0.4); rgo core-shell nanocomposite (0.4); ion release kinetics (0.4); promoter effect (0.4); respiratory deposition (0.4); biological tissue (0.4); graphene-supported ni (0.4); radiosensitive mammalian cell (0.4); nonmonotonic retention (0.4); phytoremediation system (0.4); triazine-based pollutant (0.4); combined effect (0.4); using single particle (0.4); polyvinylpyrrolidone-coated silver nanoparticle (0.4); one-step approach (0.4); nano-scale tio2 (0.4); silver sulfide nanomaterial (0.36); bacterial tactic response (0.36); amino modification (0.36); magna straus (0.36); physiochemical properties (0.36); electrochemical filter (0.36); siberian sturgeon (0.36); test media (0.36); nitrogen-fixing bacteria (0.36); potential toxicity (0.36); oxide-based nanomaterial (0.36); common aqueous antibiotic tetracycline (0.36); juvenile carp (0.36); cnt size (0.36); organic carbon (0.36); aquatic chemistry (0.36); bone marrow cell (0.33); colloidal sio2 (0.33); double-layer compression (0.33); illumination mode (0.33); sludge digestion (0.33); environmental effect (0.33); swiss-webster

					assessment (48.61, 1.0E-4); nonmetallic nanoparticle (48.47, 1.0E-4); particle size (47.99, 1.0E-4); terrestrial earthworm (47.46, 1.0E-4); aquatic ecosystem (47.32, 1.0E-4); charge behavior (45.65, 1.0E-4); micron-sized aluminum oxide (45.54, 1.0E-4); nano aluminum oxide (44.73, 1.0E-4); porous media (42.21, 1.0E-4); environmental concentration (37.52, 1.0E-4); atmospheric transformation (37.2, 1.0E-4); humic acid (34.72, 1.0E-4); au nanoparticle (33.86, 1.0E-4); surface water (33.08, 1.0E-4); modeling flow (32.3, 1.0E-4); carbon nanotube (30.95, 1.0E-4); danish environment (28.11, 1.0E-4); weighted distribution (27.98, 1.0E-4); carbonaceous nanoparticle (27.87, 1.0E-4); annual metal load (27.36, 1.0E-4); species sensitivity (24.85, 1.0E-4); natural organic matter (23.37, 1.0E-4); consumer product (23.24, 1.0E-4); ecological risk assessment (22.52, 1.0E-4); n-tio2 case study (22.52, 1.0E-4); solution chemistry (22.43, 1.0E-4); single-walled carbon nanotube (21.24, 1.0E-4); wastewater treatment (20.66, 1.0E-4); dietary exposure (17.79, 1.0E-4); adult male zebrafish (17.79, 1.0E-4); titanium dioxide (17.64, 1.0E-4); aqueous solution (17.64, 1.0E-4); multi-walled carbon nanotube (16.31, 1.0E-4); artificial sweat (15.01, 0.001); physical stress (15.01, 0.001); aquatic environment (14.36, 0.001); daphnia magna (13.35, 0.001); fullerene nanoparticle (12.97, 0.001); graphene oxide nanomaterial (12.97, 0.001); urban soil (12.42, 0.001); vitro test (12.42, 0.001); engineered nanoparticle (12.15, 0.001); exposure modeling (11.81, 0.001); geochemical reactivity (10.32, 0.005); single extraction method (10.32, 0.005); human bioaccessibility (10.32, 0.005);	mice (0.33); size characterization (0.33); containing waste incineration residue (0.33); histopathological effect (0.33); bulk zno (0.33); titanium nitride nanotube (0.33); free-living nematode caenorhabditis elegan (0.33); polymer-stabilised nanoparticle (0.33); soil mixture (0.33); pilot wastewater treatment plant (0.33); charge neutralization (0.33); electrochemical capacitive energy storage (0.33); coaxial array (0.33); multi-angle light scattering (0.33); methyl violet (0.33); field-flow fractionation (0.33); asymmetrical flow (0.33); comparative phototoxicity (0.33); electrolyte species (0.33); commercial tio2 nanoparticle (0.33); explicit fate modelling (0.33); urban soil (0.32); vitro test (0.32); bacterial community structure (0.32); screening evaluation (0.32); methodological consideration (0.32); nanoscale metal oxide (0.32); characterization factor (0.32); seventeen subcontinental freshwater (0.32); sorption behavior (0.32); field study (0.32); natural soil system (0.32); green algae (0.32); microwave-induced carbon nanotube (0.32); agcl nanoparticle (0.32); ceriodaphnia dubia (0.32); using different reducing agent (0.32); dunaliella tertiolecta (0.32);

					water purification (9.82, 0.005); ceo2 nanoparticle (9.56, 0.005); organic matter (8.18, 0.005); coated silver nanoparticle (8.18, 0.005); multiwalled carbon nanotube (8.09, 0.005); anaerobic digestion (7.92, 0.005); adverse effect (7.8, 0.01); theoretical studies (7.42, 0.01); carbon nanomaterial (7.3, 0.01); natural water (7.21, 0.01)	mediating c-60 phototransformation (0.32); chlorella vulgaris (0.32); surface complexation modeling (0.32); sewer system (0.32); inhibitory effect (0.32); tetrahymena thermophila (0.32); synergistic toxic effect (0.32); reduced graphene oxide material (0.32); polyaromatic hydrocarbon (0.32); direct feeding (0.32); different release scenario (0.32); geochemical reactivity (0.3); single extraction method (0.3); human bioaccessibility (0.3); sativus l (0.3); anatase nanoparticle (0.3); pure anatase (0.3); wheat seedling (0.3); wavelength dependency (0.3)
13	16	0.982	2013	graphene oxide; heteroaggregation; influence; nanohybrid aggregation; microparticle sedimentation; micrometer-sized hematite colloids; oxide-coated sand columns; colloidal properties; single-layered graphene oxide nanosheets; quartz | transport; porous media; effects; temperature; graphene oxide deposition; environmental concentrations; molecular mechanisms; developmental toxicity; nanohybrid aggregation; al layered double hydroxides	graphene oxide (1435.59, 1.0E-4); theoretical studies (300.52, 1.0E-4); graphene oxide nanomaterial (287.96, 1.0E-4); graphene oxide nanoparticle (262.15, 1.0E-4); carbon dot (222.28, 1.0E-4); natural mineral (177.91, 1.0E-4); natural surface water (177.64, 1.0E-4); key factor (177.64, 1.0E-4); kaolinite colloid (172.07, 1.0E-4); cation-inhibited transport (167, 1.0E-4); hofmeister effect (167, 1.0E-4); porous media (161.76, 1.0E-4); graphene oxide interaction (161.38, 1.0E-4); counterion effect (161.38, 1.0E-4); natural organic matter properties (155.76, 1.0E-4); assessing stability (149.83, 1.0E-4); enhanced dehydrochlorination (144.54, 1.0E-4); graphene-based nanomaterial (144.54, 1.0E-4); oxide-coated sand (138.93, 1.0E-4); nanohybrid aggregation (133.33, 1.0E-4); micrometer-sized hematite colloid (133.33, 1.0E-4); new insight (127.73, 1.0E-4);	semi-analytical model (0.39); low-temperature wgs reaction (0.39); ni-ceo2 catalyst (0.39); bulk counterpart (0.39); plasma mass spectrometry (0.39); fullerene df-1 (0.39); rgo core-shell nanocomposite (0.39); ion release kinetics (0.39); promoter effect (0.39); respiratory deposition (0.39); biological tissue (0.39); graphene-supported ni (0.39); radiosensitive mammalian cell (0.39); nonmonotonic retention (0.39); phytoremediation system (0.39); triazine-based pollutant (0.39); combined effect (0.39); using single particle (0.39); polyvinylpyrrolidone-coated silver nanoparticle (0.39); one-step approach

					verwey-overbeek theory (127.73, 1.0E-4); clay mineral (122.13, 1.0E-4); concurrent agglomeration (116.54, 1.0E-4); 14-labeled few-layer graphene (111.12, 1.0E-4); correlating morphology (110.96, 1.0E-4); aggregation behavior (105.62, 1.0E-4); co-existing kaolinite (105.38, 1.0E-4); surface chemistry (101.25, 1.0E-4); granular quartz sand (99.8, 1.0E-4); silver nanoparticle (94.68, 1.0E-4); heavy metal ion (94.23, 1.0E-4); synergistic coagulation (94.23, 1.0E-4); secondary adsorption (94.23, 1.0E-4); al layered double hydroxide (89.19, 1.0E-4); graphene oxide deposition (88.67, 1.0E-4); freshwater ecotoxicity (83.12, 1.0E-4); deriving characterization factor (83.12, 1.0E-4); deposition behavior (77.57, 1.0E-4); containing different metal cation (72.04, 1.0E-4); morphological transformation (72.04, 1.0E-4); facilitated bioaccumulation (66.51, 1.0E-4); remission mechanism (66.51, 1.0E-4); common carp (66.51, 1.0E-4); oxide-coated sand column (61, 1.0E-4); single-layered graphene oxide nanosheet (61, 1.0E-4); cyprinus carpio (58.52, 1.0E-4); molecular mechanism (55.5, 1.0E-4); engineered nanomaterial (47.4, 1.0E-4); bacillus subtilis (44.96, 1.0E-4); graphene concentration (44.55, 1.0E-4); colloidal properties (44.55, 1.0E-4); few-layer graphene (44.55, 1.0E-4); tio2 nanoparticle (40.61, 1.0E-4); titanium dioxide (36.78, 1.0E-4); antibacterial property (33.7, 1.0E-4); oxidation debris (30.08, 1.0E-4); natural organic matter (29.65, 1.0E-4); efficient adsorption (28.32, 1.0E-4); oxide-manganese dioxide (28.32, 1.0E-4); single-walled carbon nanotube (27.43, 1.0E-4); multi-walled carbon nanotube (27.07, 1.0E-4); fulvic acid (26.42, 1.0E-4); genuine	(0.39); nano-scale tio2 (0.39); oxide-based nanomaterial (0.35); silver sulfide nanomaterial (0.35); bacterial tactic response (0.35); amino modification (0.35); magna straus (0.35); physiochemical properties (0.35); electrochemical filter (0.35); siberian sturgeon (0.35); test media (0.35); nitrogen-fixing bacteria (0.35); potential toxicity (0.35); common aqueous antibiotic tetracycline (0.35); juvenile carp (0.35); cnt size (0.35); organic carbon (0.35); aquatic chemistry (0.35); colloidal sio2 (0.32); bone marrow cell (0.32); double-layer compression (0.32); illumination mode (0.32); sludge digestion (0.32); environmental effect (0.32); swiss-webster mice (0.32); size characterization (0.32); containing waste incineration residue (0.32); histopathological effect (0.32); bulk zno (0.32); titanium nitride nanotube (0.32); free-living nematode caenorhabditis elegan (0.32); polymer-stabilised nanoparticle (0.32); soil mixture (0.32); pilot wastewater treatment plant (0.32); charge neutralization (0.32); electrochemical capacitive energy storage (0.32); coaxial array (0.32); multi-angle light scattering (0.32); methyl violet (0.32); field-flow fractionation (0.32); asymmetrical flow

					autophagy (23, 1.0E-4); developmental toxicity (22.92, 1.0E-4); engineered nanoparticle (22.27, 1.0E-4); titanium dioxide nanoparticle (21.78, 1.0E-4); multiwalled carbon nanotube (21.05, 1.0E-4); wastewater treatment (20.07, 1.0E-4); metal oxide nanoparticle (19.21, 1.0E-4); zinc oxide nanoparticle (18.96, 1.0E-4); inorganic nanoparticle (18.1, 1.0E-4); modeling analyses (17.74, 1.0E-4); solution chemistry (17.09, 1.0E-4); natural water (16.27, 1.0E-4); environmental concentration (15.48, 1.0E-4); daphnia magna (12.96, 0.001); using different reducing agent (12.59, 0.001); reduced graphene oxide material (12.59, 0.001); fullerene nanoparticle (12.59, 0.001); arabidopsis thaliana (12.22, 0.001); zno nanoparticle (11.98, 0.001); gold nanoparticle (11.98, 0.001); particle size (11.74, 0.001); aggregation kinetics (11.36, 0.001); surface water (10.27, 0.005); eisenia fetida (10.14, 0.005); c-60 fullerene (10.02, 0.005); aqueous suspension (9.64, 0.005); water purification (9.53, 0.005); ceo2 nanoparticle (9.28, 0.005); environmental exposure (9.28, 0.005); silver ion (9.04, 0.005); probabilistic material flow analysis (8.67, 0.005); source water quality (8.19, 0.005); multiwall carbon nanotube coagulation (8.19, 0.005); organic matter (7.94, 0.005); coated silver nanoparticle (7.94, 0.005); earthworm eisenia fetida (7.94, 0.005)	(0.32); comparative phototoxicity (0.32); electrolyte species (0.32); commercial tio2 nanoparticle (0.32); explicit fate modelling (0.32); using different reducing agent (0.31); reduced graphene oxide material (0.31); characterization factor (0.31); seventeen subcontinental freshwater (0.31); sorption behavior (0.31); field study (0.31); natural soil system (0.31); green algae (0.31); microwave-induced carbon nanotube (0.31); agcl nanoparticle (0.31); ceriodaphnia dubia (0.31); urban soil (0.31); dunaliella tertiolecta (0.31); mediating c-60 phototransformation (0.31); chlorella vulgaris (0.31); surface complexation modeling (0.31); screening evaluation (0.31); methodological consideration (0.31); sewer system (0.31); inhibitory effect (0.31); vitro test (0.31); tetrahymena thermophila (0.31); nanoscale metal oxide (0.31); synergistic toxic effect (0.31); polyaromatic hydrocarbon (0.31); direct feeding (0.31); bacterial community structure (0.31); different release scenario (0.31); modeling analyses (0.29); wavelength dependency (0.29); sativus l (0.29); diesel soot (0.29); part 2-toxicity (0.29); product characterization (0.29);

						anatase nanoparticle (0.29); tio2 nanomaterial (0.29)
14	16	0.969	2010	effect; accumulation; c-60 fullerenes; plant; earthworm species; multispecies conditions; underlying causes; weathered pesticides; gold nanoparticles; arabidopsis thaliana gene expression | silver nanoparticles; changes; response; silver ions; arabidopsis thaliana gene expression; surface coating; plants; extracellular nutrient-cycling enzyme activity; bacterial community; soil slurries	plant species (165.06, 1.0E-4); c-60 fullerene (164.34, 1.0E-4); cerium uptake (144.26, 1.0E-4); surface properties (144.26, 1.0E-4); raphanus sativus (144.26, 1.0E-4); citric acid (144.26, 1.0E-4); commercial ceo2 nanoparticle (144.26, 1.0E-4); zn ion exposure (143.97, 1.0E-4); developmental responses (139.64, 1.0E-4); crop plant (139.64, 1.0E-4); nutrient status (137.85, 1.0E-4); arabidopsis thaliana seedling (135.03, 1.0E-4); arabidopsis thaliana gene expression (130.43, 1.0E-4); dde bioaccumulation (128.58, 1.0E-4); glycine max (128.58, 1.0E-4); ag nanoparticle exposure (125.85, 1.0E-4); critical review (125.12, 1.0E-4); biotic factor (123.97, 1.0E-4); aquatic macrophyte (123.97, 1.0E-4); phytotoxic hazard (120.44, 1.0E-4); related gene expression (115.78, 1.0E-4); root tissue development (115.78, 1.0E-4); photosynthetic characteristics (111.13, 1.0E-4); ulmus elongata seedling (111.13, 1.0E-4); rice plant (106.49, 1.0E-4); soil-rice system (106.49, 1.0E-4); life cycle (106.49, 1.0E-4); cucurbita pepo (106.15, 1.0E-4); weathered p (104.77, 1.0E-4); multispecies condition (101.87, 1.0E-4); earthworm species (101.87, 1.0E-4); weathered pesticide (100.11, 1.0E-4); agricultural plant (94.63, 1.0E-4); nanoscale zero-valent iron (92.69, 1.0E-4); dna-chitosan complex (88.13, 1.0E-4); nanomaterials removal (88.13, 1.0E-4); plant growth (83.59, 1.0E-4); influencing arbuscular mycorrhizal fungi effect (83.59, 1.0E-4); metal nanoparticle (83.59, 1.0E-4); zno bulk (82.8, 1.0E-4); zno np (82.8, 1.0E-4); different soil (81.65, 1.0E-4); uncoated zinc oxide nanomaterial (81.65, 1.0E-4);	semi-analytical model (0.53); low-temperature wgs reaction (0.53); ni-ceo2 catalyst (0.53); bulk counterpart (0.53); plasma mass spectrometry (0.53); fullerene df-1 (0.53); rgo core-shell nanocomposite (0.53); ion release kinetics (0.53); promoter effect (0.53); respiratory deposition (0.53); biological tissue (0.53); graphene-supported ni (0.53); radiosensitive mammalian cell (0.53); nonmonotonic retention (0.53); phytoremediation system (0.53); triazine-based pollutant (0.53); combined effect (0.53); using single particle (0.53); polyvinylpyrrolidone-coated silver nanoparticle (0.53); one-step approach (0.53); nano-scale tio2 (0.53); silver sulfide nanomaterial (0.48); bacterial tactic response (0.48); amino modification (0.48); magna straus (0.48); physiochemical properties (0.48); electrochemical filter (0.48); siberian sturgeon (0.48); test media (0.48); nitrogen-fixing bacteria (0.48); potential toxicity (0.48); oxide-based nanomaterial (0.48); common aqueous antibiotic tetracycline (0.48); juvenile carp (0.48); cnt size (0.48); organic carbon (0.48); aquatic chemistry (0.48); bone marrow

					nutritional quality (81.65, 1.0E-4); silver ion (79.89, 1.0E-4); bean seed (79.07, 1.0E-4); greenhouse condition (77.09, 1.0E-4); comparative effect (77.09, 1.0E-4); cell death (76.54, 1.0E-4); gold nanoparticle (74.99, 1.0E-4); antioxidant defence (74.58, 1.0E-4); agricultural soil (74.58, 1.0E-4); kidney bean (72.55, 1.0E-4); uncoated zno nanomaterial (72.55, 1.0E-4); phaseolus vulgaris (72.55, 1.0E-4); underlying causes (68.05, 1.0E-4); gas exchange (68.05, 1.0E-4); acute effect (65.68, 1.0E-4); graphene oxide (65.35, 1.0E-4); metal oxide nanoparticle (65.2, 1.0E-4); cuo nanoparticle (64.48, 1.0E-4); glycosyl residue (63.56, 1.0E-4); living plant cell wall (63.56, 1.0E-4); fullerene-induced increase (63.56, 1.0E-4); engineered nanomaterial (62.9, 1.0E-4); zinc oxide nanoparticle exposure (59.87, 1.0E-4); zea mays l (59.55, 1.0E-4); sewage sludge (59.12, 1.0E-4); containing multiwalled carbon nanotube (57.02, 1.0E-4); porous media (54.38, 1.0E-4); maize shoot (53.17, 1.0E-4); daucus carota (52.56, 1.0E-4); carbon nanotube (48.82, 1.0E-4); magnetic nanoparticle (48.57, 1.0E-4); different gene expression pattern (48.13, 1.0E-4); humic acid (44.73, 1.0E-4); hordeum vulgare (44.01, 1.0E-4); yield enhancement (42.18, 1.0E-4); by-2 cell suspension culture (41.71, 1.0E-4); aquatic environment (41.3, 1.0E-4); gold nanoparticle exposure (39.49, 1.0E-4); agronomical parameter (37.48, 1.0E-4); zinc oxide nanoparticle (36.9, 1.0E-4); multi-walled carbon nanotube (35.91, 1.0E-4); foeniculum vulgare mill (35.02, 1.0E-4); natural organic matter (31.75, 1.0E-4); enhanced dissolution (30.61, 1.0E-4); inositol hexakisphosphate (30.61, 1.0E-4); bulk titanium	cell (0.45); colloidal sio2 (0.45); double-layer compression (0.45); illumination mode (0.45); sludge digestion (0.45); environmental effect (0.45); swiss-webster mice (0.45); size characterization (0.45); containing waste incineration residue (0.45); histopathological effect (0.45); bulk zno (0.45); titanium nitride nanotube (0.45); free-living nematode caenorhabditis elegan (0.45); polymer-stabilised nanoparticle (0.45); soil mixture (0.45); pilot wastewater treatment plant (0.45); charge neutralization (0.45); electrochemical capacitive energy storage (0.45); coaxial array (0.45); multi-angle light scattering (0.45); methyl violet (0.45); field-flow fractionation (0.45); asymmetrical flow (0.45); comparative phototoxicity (0.45); electrolyte species (0.45); commercial tio2 nanoparticle (0.45); explicit fate modelling (0.45); methodological consideration (0.42); characterization factor (0.42); seventeen subcontinental freshwater (0.42); sorption behavior (0.42); field study (0.42); natural soil system (0.42); green algae (0.42); microwave-induced carbon nanotube (0.42); agcl nanoparticle (0.42); ceriodaphnia dubia (0.42); urban soil (0.42); using different

					dioxide (29.22, 1.0E-4); solution chemistry (28.9, 1.0E-4); wastewater treatment (26.62, 1.0E-4); soil type (26.26, 1.0E-4); ni nanoparticles phytotoxicity (26.26, 1.0E-4); wheat root (24.05, 1.0E-4); titanium dioxide (22.72, 1.0E-4); aqueous solution (22.72, 1.0E-4); natural water (21.58, 1.0E-4); complex interplay (18.8, 1.0E-4); multiwalled carbon nanotube (18.36, 1.0E-4); surface coating (17.85, 1.0E-4)	reducing agent (0.42); dunaliella tertiolecta (0.42); mediating c-60 phototransformation (0.42); chlorella vulgaris (0.42); surface complexation modeling (0.42); screening evaluation (0.42); sewer system (0.42); inhibitory effect (0.42); vitro test (0.42); tetrahymena thermophila (0.42); nanoscale metal oxide (0.42); synergistic toxic effect (0.42); reduced graphene oxide material (0.42); polyaromatic hydrocarbon (0.42); direct feeding (0.42); bacterial community structure (0.42); different release scenario (0.42); anatase nanoparticle (0.41); pure anatase (0.41); wheat seedling (0.41); wavelength dependency (0.41); sativus l (0.41); diesel soot (0.41); part 2-toxicity (0.41); product characterization (0.41)
15	12	0.945	2008	zno nanoparticles; role; morphology; aggregation kinetics; fish; nanoscale metal oxides; bioavailability; root cells; autoxidation; genotoxic effects | assessment; physico-chemical behavior; using multi-dimensional parameter testing; titanium dioxide nanoparticles; aquatic environments; root cells; autoxidation; genotoxic effects; synchrotron	zinc oxide (144.13, 1.0E-4); nano-zno particle (111.77, 1.0E-4); comparative eco-toxicities (111.77, 1.0E-4); aerosol exposure mode (110.5, 1.0E-4); relevant nanomaterial (107.22, 1.0E-4); human health risk (107.22, 1.0E-4); aerosolized transition metal oxide nanoparticle (105.34, 1.0E-4); anti-microbial activities (105.34, 1.0E-4); cu-doped tio2 nanoparticle (102.2, 1.0E-4); bacterial responses (100.8, 1.0E-4); metal oxide (96.26, 1.0E-4); developmental phytotoxicity (96.26, 1.0E-4); reactive oxygen species (90.36, 1.0E-4); using allium cepa (89.91, 1.0E-4); hazardous phytotoxic nature (87.91, 1.0E-4); bacteria-nanoparticle interaction (87.11, 1.0E-4); wastewater sludge	semi-analytical model (0.16); low-temperature wgs reaction (0.16); ni-ceo2 catalyst (0.16); bulk counterpart (0.16); plasma mass spectrometry (0.16); fullerene df-1 (0.16); rgo core-shell nanocomposite (0.16); ion release kinetics (0.16); promoter effect (0.16); respiratory deposition (0.16); biological tissue (0.16); graphene-supported ni (0.16); radiosensitive mammalian cell (0.16); nonmonotonic retention (0.16); phytoremediation system (0.16); triazine-based pollutant (0.16);

				speciation; north american water quality guidelines	(85.82, 1.0E-4); vitro cytotoxicity (83.53, 1.0E-4); using catfish (83.53, 1.0E-4); human hepg2 cell (81.61, 1.0E-4); physico-chemical behavior (77.2, 1.0E-4); facade coating (72.81, 1.0E-4); recombinant escherichia coli bacteria (70.6, 1.0E-4); toxicological assessment (70.6, 1.0E-4); root cell (64.14, 1.0E-4); allium cepa (62.1, 1.0E-4); genotoxic effect (62.1, 1.0E-4); synchrotron speciation (57.74, 1.0E-4); kaolin suspension (55.81, 1.0E-4); health effect (55.24, 1.0E-4); arabidopsis thaliana (52.22, 1.0E-4); in-vitro cell exposure studies (51.41, 1.0E-4); aerosol science (51.41, 1.0E-4); lung-a dialog (51.41, 1.0E-4); nanoparticle toxicity (47.95, 1.0E-4); numerical investigation (42.72, 1.0E-4); silver nanoparticle (42.7, 1.0E-4); inorganic nanoparticle (40.81, 1.0E-4); anisotropic porous media (40.73, 1.0E-4); nanoparticles transport (40.73, 1.0E-4); coated nickel hydroxide nanoparticle (36.35, 1.0E-4); mesquite plant (36.35, 1.0E-4); north american water quality guideline (32.03, 1.0E-4); thyroid hormone action (32.03, 1.0E-4); zno nanoparticle (32.03, 1.0E-4); particle structure (25.67, 1.0E-4); rationalizing nanomaterial size (25.67, 1.0E-4); sample preparation (25.67, 1.0E-4); atomic force microscopy (23.95, 1.0E-4); flow field-flow fractionation (23.95, 1.0E-4); dynamic light scattering (23.95, 1.0E-4); graphene oxide (22.22, 1.0E-4); phosphorus removal (21.34, 1.0E-4); metal oxide nanoparticle (19.48, 1.0E-4); biological nitrogen (19.47, 1.0E-4); porous media (18.48, 1.0E-4); humic acid (15.2, 1.0E-4); sativus l (15.03, 0.001); engineered nanomaterial (14.76, 0.001); titanium dioxide nanoparticle (14.03, 0.001); natural organic matter (13.37,	combined effect (0.16); using single particle (0.16); polyvinylpyrrolidone-coated silver nanoparticle (0.16); one-step approach (0.16); nano-scale tio2 (0.16); silver sulfide nanomaterial (0.14); bacterial tactic response (0.14); amino modification (0.14); magna straus (0.14); physiochemical properties (0.14); electrochemical filter (0.14); siberian sturgeon (0.14); test media (0.14); nitrogen-fixing bacteria (0.14); potential toxicity (0.14); oxide-based nanomaterial (0.14); common aqueous antibiotic tetracycline (0.14); juvenile carp (0.14); cnt size (0.14); organic carbon (0.14); aquatic chemistry (0.14); bone marrow cell (0.13); colloidal sio2 (0.13); double-layer compression (0.13); illumination mode (0.13); sludge digestion (0.13); environmental effect (0.13); swiss-webster mice (0.13); size characterization (0.13); containing waste incineration residue (0.13); histopathological effect (0.13); bulk zno (0.13); titanium nitride nanotube (0.13); free-living nematode caenorhabditis elegan (0.13); polymer-stabilised nanoparticle (0.13); soil mixture (0.13); pilot wastewater treatment plant (0.13); charge neutralization (0.13); electrochemical

					0.001); carbon nanotube (13, 0.001); single-walled carbon nanotube (12.37, 0.001); engineered nanoparticle (10.05, 0.005); aggregation kinetics (10.02, 0.005); solution chemistry (9.82, 0.005); zinc oxide nanoparticle (9.74, 0.005); wastewater treatment (9.05, 0.005); screening evaluation (9, 0.005); titanium dioxide (7.72, 0.01); aqueous solution (7.72, 0.01); natural water (7.33, 0.01); aquatic environment (7.26, 0.01); daphnia magna (5.84, 0.05); fullerene nanoparticle (5.68, 0.05); graphene oxide nanomaterial (5.68, 0.05); gold nanoparticle (5.4, 0.05); particle size (5.29, 0.05); surface water (4.63, 0.05); eisenia fetida (4.57, 0.05); c-60 fullerene (4.52, 0.05); multiwalled carbon nanotube (4.49, 0.05); water purification (4.3, 0.05); ceo2 nanoparticle (4.18, 0.05); environmental exposure (4.18, 0.05); silver ion (4.08, 0.05); probabilistic material flow analysis (3.91, 0.05); multi-walled carbon nanotube (3.81, 0.1); source water quality (3.69, 0.1); multiwall carbon nanotube coagulation (3.69, 0.1); zea mays l (3.63, 0.1); organic matter (3.58, 0.1); coated silver nanoparticle (3.58, 0.1); earthworm eisenia fetida (3.58, 0.1); heterotrophic wastewater biomass (3.52, 0.1); anaerobic digestion (3.47, 0.1); adverse effect (3.42, 0.1); metal ion (3.36, 0.1); plant species (3.3, 0.1); cell death (3.3, 0.1)	capacitive energy storage (0.13); coaxial array (0.13); multi-angle light scattering (0.13); methyl violet (0.13); field-flow fractionation (0.13); asymmetrical flow (0.13); comparative phototoxicity (0.13); electrolyte species (0.13); commercial tio2 nanoparticle (0.13); explicit fate modelling (0.13); screening evaluation (0.12); nanoscale metal oxide (0.12); characterization factor (0.12); seventeen subcontinental freshwater (0.12); sorption behavior (0.12); field study (0.12); natural soil system (0.12); green algae (0.12); microwave-induced carbon nanotube (0.12); agcl nanoparticle (0.12); ceriodaphnia dubia (0.12); urban soil (0.12); using different reducing agent (0.12); dunaliella tertiolecta (0.12); mediating c-60 phototransformation (0.12); chlorella vulgaris (0.12); surface complexation modeling (0.12); methodological consideration (0.12); sewer system (0.12); inhibitory effect (0.12); vitro test (0.12); tetrahymena thermophila (0.12); synergistic toxic effect (0.12); reduced graphene oxide material (0.12); polyaromatic hydrocarbon (0.12); direct feeding (0.12); bacterial community structure (0.12); different release scenario (0.12); sativus l (0.11); wavelength

						dependency (0.11); diesel soot (0.11); part 2-toxicity (0.11); product characterization (0.11); anatase nanoparticle (0.11); tio2 nanomaterial (0.11); geochemical reactivity (0.11)
17	10	0.95	2010	nanoparticles; retention; modeling; subsurface; different release scenarios; collector surface; aggregation state; affinity; experimental study; aggregated nanoparticles | effects; dissolution; ultraviolet light; silver nanoparticle mobility; functionalized gold nanoparticles; collector surface; modeling; counter ion concentration; soil microorganisms; model definition	soil column (205.24, 1.0E-4); tio2 nanoparticle sorption (146.35, 1.0E-4); natural organic matter (136.52, 1.0E-4); dissolution dynamics (134.21, 1.0E-4); select organic ligand (134.21, 1.0E-4); continental scale (131.03, 1.0E-4); functionalized gold nanoparticle (131.03, 1.0E-4); assessing environmental behavior (131.03, 1.0E-4); in-vitro nanoparticle stability testing (131.03, 1.0E-4); hydrochemical data (131.03, 1.0E-4); model definition (128.6, 1.0E-4); coagulation processes (127.96, 1.0E-4); colloidal stability (124.22, 1.0E-4); hydrochemistry influence aggregation kinetics (122.35, 1.0E-4); natural organic matter concentration (122.35, 1.0E-4); functionalized engineered nanoparticle (122.35, 1.0E-4); physicochemical properties (116.11, 1.0E-4); uncoated tio2 nanomaterial (116.11, 1.0E-4); oecd tg (116.11, 1.0E-4); tests method (116.11, 1.0E-4); counter ion concentration (107.34, 1.0E-4); titania nanoparticle stability (107.34, 1.0E-4); sand-packed column (101.07, 1.0E-4); interpreting deposition behavior (101.07, 1.0E-4); silver nanoparticle mobility (94.81, 1.0E-4); ultraviolet light (94.81, 1.0E-4); different type (92.43, 1.0E-4); quartz sand (91.73, 1.0E-4); distinguishable transport behavior (88.57, 1.0E-4); silica sand (88.57, 1.0E-4); machine learning (82.36, 1.0E-4); next generation (82.36, 1.0E-4); nanoparticle transport behavior (82.36, 1.0E-4); transport model (82.36, 1.0E-4); water treatment	nonmonotonic retention (0.23); polyvinylpyrrolidone-coated silver nanoparticle (0.23); semi-analytical model (0.23); low-temperature wgs reaction (0.23); ni-ceo2 catalyst (0.23); bulk counterpart (0.23); plasma mass spectrometry (0.23); fullerene df-1 (0.23); rgo core-shell nanocomposite (0.23); ion release kinetics (0.23); promoter effect (0.23); respiratory deposition (0.23); biological tissue (0.23); graphene-supported ni (0.23); radiosensitive mammalian cell (0.23); phytoremediation system (0.23); triazine-based pollutant (0.23); combined effect (0.23); using single particle (0.23); one-step approach (0.23); nano-scale tio2 (0.23); magna straus (0.2); physiochemical properties (0.2); test media (0.2); silver sulfide nanomaterial (0.2); bacterial tactic response (0.2); amino modification (0.2); electrochemical filter (0.2); siberian sturgeon (0.2); nitrogen-fixing bacteria (0.2); potential toxicity (0.2); oxide-based nanomaterial (0.2); common aqueous antibiotic tetracycline (0.2); juvenile carp

					(73.58, 1.0E-4); source water (70, 1.0E-4); titanium dioxide nanoparticle removal (70, 1.0E-4); surface-modified nanoparticle (64.69, 1.0E-4); environmental system (63.87, 1.0E-4); theoretical analysis (63.87, 1.0E-4); rapid settling (57.77, 1.0E-4); inorganic nanoparticle (51.97, 1.0E-4); nanoparticle core properties (48.69, 1.0E-4); macromolecule-coated nanoparticle (48.69, 1.0E-4); carbon mineralization (42.55, 1.0E-4); bacterial abundance (42.55, 1.0E-4); soil properties (42.55, 1.0E-4); natural antidote (37.3, 1.0E-4); different source (33.63, 1.0E-4); monovalent electrolyte (33.63, 1.0E-4); gold nanoparticle aggregation (33.63, 1.0E-4); natural organic matter sample (33.63, 1.0E-4); graphene oxide (31.22, 1.0E-4); engineered nanomaterial (30.04, 1.0E-4); tio2 nanoparticle (28.51, 1.0E-4); soil microorganism (27.41, 1.0E-4); functionalized single-wall carbon nanotube (27.41, 1.0E-4); carbon nanotube (27.3, 1.0E-4); silver nanoparticle (27.07, 1.0E-4); porous media (25.97, 1.0E-4); engineered nanoparticle (24.35, 1.0E-4); humic acid (21.37, 1.0E-4); collector surface (21.26, 1.0E-4); aggregated nanoparticle (21.26, 1.0E-4); aquatic environment (19.72, 1.0E-4); hematite nanoparticle (19.48, 1.0E-4); single-walled carbon nanotube (17.39, 1.0E-4); multi-walled carbon nanotube (17.16, 1.0E-4); different release scenario (15.22, 1.0E-4); titanium dioxide nanoparticle (13.8, 0.001); magna straus (13.09, 0.001); physiochemical properties (13.09, 0.001); test media (13.09, 0.001); wastewater treatment (12.72, 0.001); zinc oxide nanoparticle (12.18, 0.001); metal oxide nanoparticle (12.17, 0.001); natural water (10.95, 0.001);	(0.2); cnt size (0.2); organic carbon (0.2); aquatic chemistry (0.2); bone marrow cell (0.19); colloidal sio2 (0.19); double-layer compression (0.19); illumination mode (0.19); sludge digestion (0.19); environmental effect (0.19); swiss-webster mice (0.19); size characterization (0.19); containing waste incineration residue (0.19); histopathological effect (0.19); bulk zno (0.19); titanium nitride nanotube (0.19); free-living nematode caenorhabditis elegan (0.19); polymer-stabilised nanoparticle (0.19); soil mixture (0.19); pilot wastewater treatment plant (0.19); charge neutralization (0.19); electrochemical capacitive energy storage (0.19); coaxial array (0.19); multi-angle light scattering (0.19); methyl violet (0.19); field-flow fractionation (0.19); asymmetrical flow (0.19); comparative phototoxicity (0.19); electrolyte species (0.19); commercial tio2 nanoparticle (0.19); explicit fate modelling (0.19); different release scenario (0.18); characterization factor (0.18); seventeen subcontinental freshwater (0.18); sorption behavior (0.18); field study (0.18); natural soil system (0.18); green algae (0.18); microwave-induced carbon nanotube (0.18); agcl nanoparticle

					titanium dioxide (10.85, 0.001); aqueous solution (10.85, 0.001); daphnia magna (8.21, 0.005); fullerene nanoparticle (7.98, 0.005); graphene oxide nanomaterial (7.98, 0.005); arabidopsis thaliana (7.74, 0.01); zno nanoparticle (7.59, 0.01); gold nanoparticle (7.59, 0.01); particle size (7.44, 0.01); aggregation kinetics (7.2, 0.01); nonmonotonic retention (6.54, 0.05); polyvinylpyrrolidone-coated silver nanoparticle (6.54, 0.05); surface water (6.51, 0.05); eisenia fetida (6.42, 0.05); c-60 fullerene (6.35, 0.05); water purification (6.04, 0.05); ceo2 nanoparticle (5.88, 0.05); environmental exposure (5.88, 0.05); silver ion (5.73, 0.05); probabilistic material flow analysis (5.49, 0.05); source water quality (5.19, 0.05); multiwall carbon nanotube coagulation (5.19, 0.05); organic matter (5.03, 0.05)	(0.18); ceriodaphnia dubia (0.18); urban soil (0.18); using different reducing agent (0.18); dunaliella tertiolecta (0.18); mediating c-60 phototransformation (0.18); chlorella vulgaris (0.18); surface complexation modeling (0.18); screening evaluation (0.18); methodological consideration (0.18); sewer system (0.18); inhibitory effect (0.18); vitro test (0.18); tetrahymena thermophila (0.18); nanoscale metal oxide (0.18); synergistic toxic effect (0.18); reduced graphene oxide material (0.18); polyaromatic hydrocarbon (0.18); direct feeding (0.18); bacterial community structure (0.18); collector surface (0.17); aggregated nanoparticle (0.17); wavelength dependency (0.17); sativus l (0.17); diesel soot (0.17); part 2-toxicity (0.17); product characterization (0.17); anatase nanoparticle (0.17)
18	8	0.989	2008	adsorption; carbon nanotubes; atrazine; natural organic matter; engineered nanomaterials; sorption; available carbon nanotube; water; metal impurities; black carbon | multi-walled carbon nanotubes; tetracycline; aqueous solution chemistry; black carbon; thermodynamics; polyaromatic	multi-walled carbon nanotube (121.44, 1.0E-4); aqueous solution chemistry (103.34, 1.0E-4); carbon nanotube (102.49, 1.0E-4); competitive adsorption (95.46, 1.0E-4); monoaromatic compound (88.81, 1.0E-4); pharmaceutical antibiotics (82.51, 1.0E-4); adsorbed phenanthrene (82.16, 1.0E-4); black carbon (82.16, 1.0E-4); surface-modified carbon nanotube (75.53, 1.0E-4); available carbon nanotube (68.92, 1.0E-4); synthetic organic chemical (64.09, 1.0E-4); background solution chemistry (64.09, 1.0E-4); alpha-ethinyl estradiol (57.56,	phytoremediation system (0.04); semi-analytical model (0.04); low-temperature wgs reaction (0.04); ni-ceo2 catalyst (0.04); bulk counterpart (0.04); plasma mass spectrometry (0.04); fullerene df-1 (0.04); rgo core-shell nanocomposite (0.04); ion release kinetics (0.04); promoter effect (0.04); respiratory deposition (0.04); biological tissue (0.04); graphene-supported ni (0.04); radiosensitive

				hydrocarbons; pharmaceutical antibiotics; multiwalled carbon nanotubes; surface-modified carbon nanotubes; agrobacterium	1.0E-4); adsorption kinetics (57.56, 1.0E-4); multiwalled carbon nanotube (50.74, 1.0E-4); indoor corona device (44.65, 1.0E-4); ionizable aromatic compound (38.36, 1.0E-4); oxidized multiwalled carbon nanotube (38.36, 1.0E-4); other natural adsorbent (32.35, 1.0E-4); carbon nanomaterial (28.37, 1.0E-4); sorption behavior (23.52, 1.0E-4); natural soil system (23.52, 1.0E-4); polyaromatic hydrocarbon (23.52, 1.0E-4); different type (15.12, 0.001); silver nanoparticle (14.74, 0.001); phytoremediation system (9.32, 0.005); graphene oxide (7.67, 0.01); engineered nanomaterial (7.6, 0.01); porous media (6.38, 0.05); tio2 nanoparticle (6.32, 0.05); natural organic matter (6.28, 0.05); aqueous solution (5.92, 0.05); humic acid (5.25, 0.05); aquatic environment (4.84, 0.05); single-walled carbon nanotube (4.27, 0.05); fulvic acid (3.49, 0.1); engineered nanoparticle (3.47, 0.1); titanium dioxide nanoparticle (3.39, 0.1); solution chemistry (3.39, 0.1); wastewater treatment (3.12, 0.1); metal oxide nanoparticle (2.99, 0.1); zinc oxide nanoparticle (2.96, 0.1); inorganic nanoparticle (2.82, 0.1); titanium dioxide (2.67, 0.5); natural water (2.53, 0.5); daphnia magna (2.02, 0.5); graphene oxide nanomaterial (1.96, 0.5); arabidopsis thaliana (1.9, 0.5); zno nanoparticle (1.86, 0.5); gold nanoparticle (1.86, 0.5); particle size (1.83, 0.5); aggregation kinetics (1.77, 0.5); surface water (1.6, 0.5); eisenia fetida (1.58, 0.5); c-60 fullerene (1.56, 0.5); water purification (1.48, 0.5); ceo2 nanoparticle (1.44, 0.5); environmental exposure (1.44, 0.5); silver ion (1.41, 0.5); probabilistic material flow analysis (1.35, 0.5); source water quality (1.27, 0.5);	mammalian cell (0.04); nonmonotonic retention (0.04); triazine-based pollutant (0.04); combined effect (0.04); using single particle (0.04); polyvinylpyrrolidone-coated silver nanoparticle (0.04); one-step approach (0.04); nano-scale tio2 (0.04); silver sulfide nanomaterial (0.04); bacterial tactic response (0.04); amino modification (0.04); magna straus (0.04); physiochemical properties (0.04); electrochemical filter (0.04); siberian sturgeon (0.04); test media (0.04); nitrogen-fixing bacteria (0.04); potential toxicity (0.04); oxide-based nanomaterial (0.04); common aqueous antibiotic tetracycline (0.04); juvenile carp (0.04); cnt size (0.04); organic carbon (0.04); aquatic chemistry (0.04); bone marrow cell (0.03); colloidal sio2 (0.03); double-layer compression (0.03); illumination mode (0.03); sludge digestion (0.03); environmental effect (0.03); swiss-webster mice (0.03); size characterization (0.03); containing waste incineration residue (0.03); histopathological effect (0.03); bulk zno (0.03); titanium nitride nanotube (0.03); free-living nematode caenorhabditis elegan (0.03); polymer-stabilised nanoparticle (0.03); soil mixture

					multiwall carbon nanotube coagulation (1.27, 0.5); organic matter (1.23, 0.5); coated silver nanoparticle (1.23, 0.5); earthworm eisenia fetida (1.23, 0.5); heterotrophic wastewater biomass (1.21, 0.5); anaerobic digestion (1.2, 0.5); adverse effect (1.18, 0.5); metal ion (1.16, 0.5); plant species (1.14, 0.5); cell death (1.14, 0.5); nanowaste detection (1.12, 0.5); theoretical studies (1.12, 0.5); irish surface (1.1, 0.5); cuo nanoparticle (1.1, 0.5); regulatory system (1.1, 0.5); nano-scale pollutant (1.1, 0.5); drinking water (1.1, 0.5); environmental risk assessment (1.06, 0.5); facile synthesis (1.06, 0.5); copper nanoparticle (1.04, 0.5); suwannee river (1.04, 0.5); nanocopper removal (1.02, 0.5); graphene oxide nanoparticle (1.02, 0.5); comparative study (1.02, 0.5); algal toxicity (1.02, 0.5); municipal wastewater (1.02, 0.5); modeling technique (1.02, 0.5); chemical transformation (1.01, 0.5); physicochemical interaction (1.01, 0.5); silica nanoparticle (1.01, 0.5); environmental factor (1.01, 0.5); high-throughput screening (0.99, 0.5); danio rerio (0.99, 0.5); toxicity effect (0.97, 0.5); cucurbita pepo (0.97, 0.5); microbial communities (0.95, 0.5); aerosol exposure mode (0.93, 0.5); trophic transfer (0.93, 0.5); chemical speciation (0.91, 0.5)	(0.03); pilot wastewater treatment plant (0.03); charge neutralization (0.03); electrochemical capacitive energy storage (0.03); coaxial array (0.03); multi-angle light scattering (0.03); methyl violet (0.03); field-flow fractionation (0.03); asymmetrical flow (0.03); comparative phototoxicity (0.03); electrolyte species (0.03); commercial tio2 nanoparticle (0.03); explicit fate modelling (0.03); sorption behavior (0.03); natural soil system (0.03); polyaromatic hydrocarbon (0.03); characterization factor (0.03); seventeen subcontinental freshwater (0.03); field study (0.03); green algae (0.03); microwave-induced carbon nanotube (0.03); agcl nanoparticle (0.03); ceriodaphnia dubia (0.03); urban soil (0.03); using different reducing agent (0.03); dunaliella tertiolecta (0.03); mediating c-60 phototransformation (0.03); chlorella vulgaris (0.03); surface complexation modeling (0.03); screening evaluation (0.03); methodological consideration (0.03); sewer system (0.03); inhibitory effect (0.03); vitro test (0.03); tetrahymena thermophila (0.03); nanoscale metal oxide (0.03); synergistic toxic effect (0.03); reduced graphene oxide material (0.03); direct feeding (0.03); bacterial

						community structure (0.03); different release scenario (0.03); other natural adsorbent (0.03); wavelength dependency (0.03); sativus l (0.03); diesel soot (0.03); part 2-toxicity (0.03); product characterization (0.03); anatase nanoparticle (0.03); tio2 nanomaterial (0.03)

Table.S2 Network summary of co-citation analysis by Citespace

Burst	Centrality	Sigma	PageRank	Keyword	Author	Year	Title	Source	Vol	Page	Half-life	ClusterID
	0.01	1.00	1.01		Gottschalk F	2009	...	ENVIRON SCI TECHNOL	43	9216	6	12
	0.00	1.00	0.60		Klaine SJ	2008	...	ENVIRON TOXICOL CHEM	27	1825	5	12
	0.47	1.00	1.30		Mueller NC	2008	...	ENVIRON SCI TECHNOL	42	4447	5	12
6.32	0.01	1.06	1.12		Nowack B	2007	...	ENVIRON POLLUT	150	5	5	0
6.37	0.00	1.00	0.58		Nel A	2006	...	SCIENCE	311	622	5	6
4.99	0.01	1.04	1.09		Keller AA	2010	...	ENVIRON SCI TECHNOL	44	1962	5	5
27.42	0.02	1.59	1.04		Hyung H	2007	...	ENVIRON SCI TECHNOL	41	179	4	0
4.66	0.07	1.36	1.82		Petosa AR	2010	...	ENVIRON SCI TECHNOL	44	6532	5	17
17.90	0.00	1.00	0.49		Wiesner MR	2006	...	ENVIRON SCI TECHNOL	40	4336	5	7
4.42	0.03	1.16	0.98		Benn TM	2008	...	ENVIRON SCI TECHNOL	42	4133	6	3
32.07	0.03	2.25	1.85		Oberdorster G	2005	...	ENVIRON HEALTH PERSP	113	823	6	1
31.46	0.00	1.00	0.57		Lowry GV	2012	...	ENVIRON SCI TECHNOL	46	6893	3	11
31.61	0.00	1.00	0.52		Gottschalk F	2013	...	ENVIRON POLLUT	181	287	3	3
4.77	0.06	1.32	2.26		Pan B	2008	...	ENVIRON SCI TECHNOL	42	9005	5	18
	0.06	1.00	1.90		Kiser MA	2009	...	ENVIRON SCI TECHNOL	43	6757	5	12
	0.08	1.00	2.29		Aruoja V	2009	...	SCI TOTAL ENVIRON	407	1461	5	8
3.74	0.03	1.10	2.08		Liu JY	2010	...	ENVIRON SCI TECHNOL	44	2169	4	3
	0.01	1.00	1.04		Mauter MS	2008	...	ENVIRON SCI TECHNOL	42	5843	5	18
	0.31	1.00	2.18		Heinlaan M	2008	...	CHEMOSPHERE	71	1308	5	8
6.19	0.00	1.00	0.63		Navarro E	2008	...	ECOTOXICOLOGY	17	372	5	0
2.81	0.17	1.56	2.21		Chen KL	2007	...	J COLLOID INTERF SCI	309	126	5	5
43.93	0.73	27472009591.68	1.27		Oberdorster E	2004	...	ENVIRON HEALTH PERSP	112	1058	5	4
3.85	0.56	5.51	2.15		Kaegi R	2008	...	ENVIRON POLLUT	156	233	5	12
7.22	0.22	4.16	3.55		Franklin NM	2007	...	ENVIRON SCI TECHNOL	41	8484	5	15
	0.20	1.00	3.13		Kaegi R	2011	...	ENVIRON SCI TECHNOL	45	3902	4	3
	0.01	1.00	1.07		Griffitt RJ	2008	...	ENVIRON TOXICOL CHEM	27	1972	5	12
8.18	0.02	1.15	1.55		Petersen EJ	2011	...	ENVIRON SCI TECHNOL	45	9837	4	9
11.45	0.01	1.10	1.09		Handy RD	2008	...	ECOTOXICOLOGY	17	287	4	15
15.70	0.19	15.17	2.33		Yang K	2006	...	ENVIRON SCI TECHNOL	40	1855	5	0
10.94	0.15	4.53	1.35		Levard C	2012	...	ENVIRON SCI TECHNOL	46	6900	3	11
	0.00	1.00	0.52		Handy RD	2008	...	ECOTOXICOLOGY	17	315	5	4
35.25	0.24	1972.02	2.48		Fortner JD	2005	...	ENVIRON SCI TECHNOL	39	4307	4	6
	0.02	1.00	1.51		Navarro E	2008	...	ENVIRON SCI TECHNOL	42	8959	5	8
	0.08	1.00	1.88		French RA	2009	...	ENVIRON SCI TECHNOL	43	1354	6	5

3.81	0.03	1.14	1.44		Hyung H	2008	...	ENVIRON SCI TECHNOL	42	4416	5	0
9.63	0.02	1.18	1.49		Gottschalk F	2011	...	J ENVIRON MONITOR	13	1145	4	2
3.64	0.10	1.42	1.86		Fabrega J	2011	...	ENVIRON INT	37	517	4	11
31.53	0.03	2.22	1.43		Sun TY	2014	...	ENVIRON POLLUT	185	69	2	2
40.59	0.01	1.41	1.01		Colvin VL	2003	...	NAT BIOTECHNOL	21	1166	6	6
12.50	0.04	1.68	2.17		Nowack B	2012	...	ENVIRON TOXICOL CHEM	31	50	3	2
5.90	0.01	1.05	1.20		Chen W	2007	...	ENVIRON SCI TECHNOL	41	8295	5	0
16.69	0.03	1.75	1.41		Moore MN	2006	...	ENVIRON INT	32	967	5	4
11.58	0.08	2.32	0.88		Chen KL	2006	...	LANGMUIR	22	10994	6	17
	0.48	1.00	2.52		Blaser SA	2008	...	SCI TOTAL ENVIRON	390	396	5	3
12.18	0.19	8.01	1.76		Dreyer DR	2010	...	CHEM SOC REV	39	228	5	7
27.38	0.87	26340061.88	2.94		Lovern SB	2006	...	ENVIRON TOXICOL CHEM	25	1132	4	4
9.83	0.01	1.09	0.99		Lam CW	2006	...	CRIT REV TOXICOL	36	189	5	1
10.55	0.08	2.33	0.92		Weir A	2012	...	ENVIRON SCI TECHNOL	46	2242	3	12
6.24	0.12	2.07	1.70		Lin DH	2007	...	ENVIRON POLLUT	150	243	6	14
20.80	0.00	1.00	0.53		Adams LK	2006	...	WATER RES	40	3527	5	15
	0.01	1.00	1.02		Zhang Y	2009	...	WATER RES	43	4249	6	5
23.61	0.52	19605.86	1.61		Lecoanet HF	2004	...	ENVIRON SCI TECHNOL	38	5164	6	7
	0.03	1.00	2.47		Kim B	2010	...	ENVIRON SCI TECHNOL	44	7509	5	11
	0.03	1.00	1.44		Yang K	2010	...	CHEM REV	110	5989	4	0
	0.00	1.00	0.55		Chen X	2007	...	CHEM REV	107	2891	6	5
29.35	0.78	22669235.02	1.90		Sayes CM	2004	...	NANO LETT	4	1881	5	6
	0.58	1.00	2.60		Federici G	2007	...	AQUAT TOXICOL	84	415	5	4
29.35	0.05	4.30	2.90		Lam CW	2004	...	TOXICOL SCI	77	126	5	1
9.61	0.00	1.00	0.56		Brar SK	2010	...	WASTE MANAGE	30	504	5	14
	0.05	1.00	2.94		Domingos RF	2009	...	ENVIRON SCI TECHNOL	43	1282	6	5
13.45	0.06	2.17	1.90		Zhao GX	2011	...	ENVIRON SCI TECHNOL	45	10454	5	10
21.59	0.00	1.00	0.60		Piccinno F	2012	...	J NANOPART RES	14	0	5	12
14.62	0.32	58.21	1.68		Chowdhury I	2013	...	ENVIRON SCI TECHNOL	47	6288	3	7
4.31	0.03	1.11	0.99		Kahru A	2010	...	TOXICOLOGY	269	105	4	8
6.29	0.03	1.23	2.43		Rico CM	2011	...	J AGR FOOD CHEM	59	3485	4	14
6.27	0.00	1.00	0.52		Baun A	2008	...	ECOTOXICOLOGY	17	387	4	4
5.13	0.04	1.24	0.89		Lin DH	2008	...	ENVIRON SCI TECHNOL	42	7254	4	0
15.24	0.08	3.03	1.43		Fabrega J	2009	...	ENVIRON SCI TECHNOL	43	7285	4	11
19.51	0.73	42390.87	1.31		Zhu SQ	2006	...	MAR ENVIRON RES	62	0	4	4
20.00	0.00	1.00	0.90		Akhavan O	2010	...	ACS NANO	4	5731	6	13
14.46	0.02	1.28	0.96		Saleh NB	2008	...	ENVIRON SCI TECHNOL	42	7963	4	9
14.35	0.32	51.58	0.84		Chen KL	2008	...	ENVIRON SCI TECHNOL	42	7607	5	7
19.04	0.00	1.00	0.52		Keller AA	2013	...	J NANOPART RES	15	0	3	2
3.26	0.93	8.52	2.22		Jia G	2005	...	ENVIRON SCI TECHNOL	39	1378	5	1
9.82	0.00	1.00	0.54		Poland CA	2008	...	NAT NANOTECHNOL	3	423	4	1
18.40	0.00	1.00	0.59		De V	2013	...	SCIENCE	339	535	3	9
5.84	0.00	1.00	0.57		Baun A	2008	...	AQUAT TOXICOL	86	379	6	4
16.34	0.00	1.00	0.51		Maynard AD	2006	...	NATURE	444	267	4	7

17.45	0.01	1.16	1.02		Marcano DC	2010	...	ACS NANO	4	4806	6	7
9.28	0.02	1.17	1.59		Menard A	2011	...	ENVIRON POLLUT	159	677	4	8
14.38	0.01	1.13	1.11		Choi O	2008	...	ENVIRON SCI TECHNOL	42	4583	4	11
11.94	0.08	2.38	1.41		Robichaud CO	2009	...	ENVIRON SCI TECHNOL	43	4227	6	12
9.83	0.02	1.18	0.99		Xia T	2008	...	ACS NANO	2	2121	4	15
15.98	0.00	1.00	0.55		Gao J	2009	...	ENVIRON SCI TECHNOL	43	3322	3	11
10.53	0.15	4.25	1.83		Geim AK	2007	...	NAT MATER	6	183	7	10
3.49	0.04	1.16	2.64		Petersen EJ	2009	...	ENVIRON SCI TECHNOL	43	2969	4	9
13.85	0.14	6.08	1.34		Lin DH	2008	...	ENVIRON SCI TECHNOL	42	5580	4	15
15.49	0.07	2.75	1.78		Roberts AP	2007	...	ENVIRON SCI TECHNOL	41	3025	3	9
19.53	0.03	1.64	1.43		Lyon DY	2006	...	ENVIRON SCI TECHNOL	40	4360	3	6
16.49	0.00	1.00	0.53		Kaegi R	2013	...	WATER RES	47	3866	3	3
16.17	0.01	1.15	0.99		Oberdorster E	2006	...	CARBON	44	1112	5	4
16.17	0.00	1.00	0.53		Smith CJ	2007	...	AQUAT TOXICOL	82	94	3	9
	0.03	1.00	0.90		Zhao J	2014	...	ENVIRON SCI TECHNOL	48	9995	3	13
16.97	0.01	1.16	0.99		Lovern SB	2007	...	ENVIRON SCI TECHNOL	41	4465	4	4
19.79	0.03	1.64	0.77		Peng XJ	2003	...	CHEM PHYS LETT	376	154	6	0
16.47	0.08	3.74	1.24		von der K	2012	...	ENVIRON TOXICOL CHEM	31	32	2	2
10.19	0.01	1.09	1.10		Huynh KA	2011	...	ENVIRON SCI TECHNOL	45	5564	4	17
11.38	0.02	1.21	1.56		Limbach LK	2008	...	ENVIRON SCI TECHNOL	42	5828	4	12
	0.00	1.00	0.15		Vance ME	2015	...	BEILSTEIN J NANOTECH	6	1769	2	34
11.84	0.00	1.00	0.51		Wiesner MR	2009	...	ENVIRON SCI TECHNOL	43	6458	3	3
7.18	0.00	1.00	0.52		Henry TB	2007	...	ENVIRON HEALTH PERSP	115	1059	4	6
13.94	0.00	1.00	0.53		Ma HB	2013	...	ENVIRON POLLUT	172	76	3	15
13.94	0.00	1.00	0.57		Lowry GV	2012	...	ENVIRON SCI TECHNOL	46	7027	4	11
13.94	0.00	1.00	0.57		Ma R	2014	...	ENVIRON SCI TECHNOL	48	104	2	11
8.20	0.03	1.23	0.93		Jaisi DP	2009	...	ENVIRON SCI TECHNOL	43	9161	4	7
14.40	0.00	1.00	0.56		Peralta-videa JR	2011	...	J HAZARD MATER	186	1	3	14
14.40	0.01	1.13	1.02		Stankovich S	2007	...	CARBON	45	1558	7	7
12.35	0.03	1.37	1.53		Morones JR	2005	...	NANOTECHNOLOGY	16	2346	7	11
13.14	0.00	1.00	0.54		Geim AK	2009	...	SCIENCE	324	1530	6	10
9.86	0.00	1.00	0.62		El BAM	2010	...	ENVIRON SCI TECHNOL	44	1260	4	17
12.67	0.03	1.53	1.40		Liu HH	2014	...	ENVIRON SCI TECHNOL	48	3281	2	2
23.62	0.07	4.73	3.02		Deguchi S	2001	...	LANGMUIR	17	6013	7	6
4.30	0.12	1.66	2.28		Li D	2008	...	NAT NANOTECHNOL	3	101	6	10
	0.04	1.00	1.41		Ma XM	2010	...	SCI TOTAL ENVIRON	408	3053	6	14
22.81	0.28	259.27	2.48		Long RQ	2001	...	J AM CHEM SOC	123	2058	7	0
10.67	0.38	31.38	1.69		Lecoanet HF	2004	...	ENVIRON SCI TECHNOL	38	4377	6	7

19.93	0.14	14.11	1.22		Brant J	2005	...	ENVIRON SCI TECHNOL	39	6343	4	6
	0.01	1.00	1.06		Keller AA	2014	...	ENVIRON SCI TECH LET	1	65	3	2
	0.00	1.00	0.53		Zhu XS	2010	...	CHEMOSPHERE	78	209	4	8
13.03	0.03	1.54	0.88		Kang S	2008	...	LANGMUIR	24	6409	6	1
5.66	0.00	1.00	0.15		Nel AE	2009	...	NAT MATER	8	543	5	31
13.32	0.00	1.00	0.57	5	Auffan M	2009	...	NAT NANOTECHNOL	4	634	4	5
8.87	0.00	1.00	0.60		Zhang Y	2008	...	WATER RES	42	2204	4	5
8.46	0.03	1.24	1.47		Kang S	2007	...	LANGMUIR	23	8670	6	1
11.85	0.01	1.11	1.07		Westerhoff P	2011	...	J ENVIRON MONITOR	13	1195	4	12
14.97	0.35	93.00	2.21		Hund-rinke K	2006	...	ENVIRON SCI POLLUT R	13	225	5	8
13.07	0.02	1.25	1.42		Cho HH	2008	...	ENVIRON SCI TECHNOL	42	2899	3	0
	0.00	1.00	0.53		Pan B	2008	...	ENVIRON SCI TECHNOL	42	5480	6	18
12.96	0.03	1.54	1.23		Li YS	2008	...	ENVIRON SCI TECHNOL	42	7174	5	7
11.00	0.01	1.10	1.04		Rao GP	2007	...	SEP PURIF TECHNOL	58	224	5	0
	0.02	1.00	1.30		Liu SB	2011	...	ACS NANO	5	6971	6	13
	0.00	1.00	0.62		Xiu ZM	2012	...	NANO LETT	12	4271	4	11
16.76	0.00	1.00	0.51		Aitken RJ	2006	...	OCCUP MED-OXFORD	56	300	5	4
	0.00	1.00	1.12		Gupta VK	2013	...	ADV COLLOID INTERFAC	193	24	3	16
7.72	0.03	1.21	1.28		Klaine SJ	2012	...	ENVIRON TOXICOL CHEM	31	3	2	2
	0.01	1.00	1.01		Keller AA	2013	...	J NANOPART RES	15	1	2	3
16.30	0.00	1.00	0.56		Saleh NB	2010	...	ENVIRON SCI TECHNOL	44	2412	2	0
13.81	0.21	14.19	2.61		Shvedova AA	2003	...	J TOXICOL ENV HEAL A	66	1909	7	1
	0.00	1.00	0.56		Gardea-torresdey JL	2014	...	ENVIRON SCI TECHNOL	48	2526	3	14
	0.00	1.00	0.61		Bian SW	2011	...	LANGMUIR	27	6059	6	5
13.35	0.00	1.00	0.53		Wang HH	2009	...	ENVIRON POLLUT	157	1171	2	15
	0.08	1.00	1.41		Gao Y	2012	...	J COLLOID INTERF SCI	368	540	4	10
	0.00	1.00	0.52		Ju-nam Y	2008	...	SCI TOTAL ENVIRON	400	396	5	12
11.11	0.01	1.10	1.07		Xie B	2008	...	ENVIRON SCI TECHNOL	42	2853	2	6
	0.00	1.00	1.12		Saleh TA	2012	...	J COLLOID INTERF SCI	371	101	4	16
	0.00	1.00	0.60		Lanphere JD	2013	...	ENVIRON SCI TECHNOL	47	4255	4	13
	0.00	1.00	0.58		Zhu XS	2008	...	J ENVIRON SCI HEAL A	43	278	7	4
7.35	0.00	1.00	0.57		Yang K	2009	...	LANGMUIR	25	3571	3	5
14.86	0.01	1.14	1.11		Jaisi DP	2008	...	ENVIRON SCI TECHNOL	42	8317	5	7
6.98	0.00	1.00	0.55		Jiang JK	2009	...	J NANOPART RES	11	77	2	5
13.62	0.19	10.54	2.09		Praetorius A	2012	...	ENVIRON SCI TECHNOL	46	6705	3	2
	0.00	1.00	0.52		Farre M	2009	...	ANAL BIOANAL CHEM	393	81	4	8
14.17	0.00	1.00	0.57		Hendren CO	2011	...	ENVIRON SCI TECHNOL	45	2562	3	2
	0.12	1.00	2.17		Wu L	2013	...	LANGMUIR	29	15174	4	13

	0.00	1.00	0.57		Thio BJR	2011	...	J HAZARD MATER	189	556	6	5
15.65	0.00	1.00	0.50		Sayes CM	2005	...	BIOMATERIALS	26	7587	4	6
	0.02	1.00	1.52		Wang XL	2009	...	ENVIRON SCI TECHNOL	43	6214	4	18
15.13	0.01	1.14	1.01		Brant JA	2006	...	LANGMUIR	22	3878	3	6
12.74	0.00	1.00	0.59		Levard C	2011	...	ENVIRON SCI TECHNOL	45	5260	5	3
	0.01	1.00	1.03		Levard C	2013	...	ENVIRON SCI TECHNOL	47	13440	3	3
	0.02	1.00	1.04		Ren XM	2011	...	CHEM ENG J	170	395	6	0
12.30	0.02	1.23	1.47		Quik JTK	2014	...	WATER RES	48	269	1	2
	0.05	1.00	1.39		Geranio L	2009	...	ENVIRON SCI TECHNOL	43	8113	7	3
13.78	0.23	16.82	0.82		Lyon DY	2005	...	ENVIRON TOXICOL CHEM	24	2757	3	6
13.64	0.00	1.00	0.50		Warheit DB	2004	...	TOXICOL SCI	77	117	5	1
	0.00	1.00	0.52		Chen D	2012	...	CHEM REV	112	6027	4	13
	0.00	1.00	1.54		Gupta VK	2012	...	RSC ADV	2	6380	4	16
12.04	0.02	1.23	1.02		Canesi L	2012	...	MAR ENVIRON RES	76	16	3	4
	0.23	1.00	2.16		Meesters JAJ	2014	...	ENVIRON SCI TECHNOL	48	5726	1	3
13.27	0.00	1.00	0.82		Li YH	2003	...	CARBON	41	1057	6	0
12.09	0.04	1.65	0.89		Wang XL	2008	...	ENVIRON SCI TECHNOL	42	3207	2	0
6.48	0.15	2.46	1.68		Kennedy AJ	2008	...	ENVIRON TOXICOL CHEM	27	1932	3	9
	0.00	1.00	0.52		Kaegi R	2010	...	ENVIRON POLLUT	158	2900	5	12
	0.00	1.00	0.53		Petersen EJ	2009	...	ENVIRON SCI TECHNOL	43	4181	8	18
	0.00	1.00	0.55		Marambio-jones C	2010	...	J NANOPART RES	12	1531	6	11
	0.02	1.00	1.03		Chandra V	2010	...	ACS NANO	4	3979	7	10
12.76	0.04	1.70	1.89		Li YH	2002	...	CHEM PHYS LETT	357	263	7	0
	0.00	1.00	0.58		Lin DH	2010	...	J ENVIRON QUAL	39	1896	5	5
	0.00	1.00	0.55		Ji LL	2009	...	ENVIRON SCI TECHNOL	43	2322	7	10
11.50	0.07	2.21	1.29		Lin SJ	2009	...	SMALL	5	1128	3	14
10.54	0.00	1.00	0.54		Zhu YW	2010	...	ADV MATER	22	3906	5	10
8.63	0.00	1.00	0.61		Ferry JL	2009	...	NAT NANOTECHNOL	4	441	3	2
10.54	0.00	1.00	0.59		Wang YF	2012	...	J HAZARD MATER	201	16	3	12
	0.00	1.00	0.51		Boxall ABA	2007	...	CURRENT FUTURE PREDI	0	0	8	3
11.12	0.00	1.00	0.54		Novoselov KS	2004	...	SCIENCE	306	666	8	10
9.30	0.04	1.47	1.88		Lin DH	2008	...	ENVIRON SCI TECHNOL	42	5917	4	9
8.74	0.02	1.16	1.01		Stoller MD	2008	...	NANO LETT	8	3498	5	10
	0.00	1.00	0.15		Batley GE	2013	...	ACCOUNTS CHEM RES	46	854	4	48
11.02	0.01	1.10	1.08		Smith B	2009	...	LANGMUIR	25	9767	3	9
	0.03	1.00	1.51		Lopez-moreno ML	2010	...	ENVIRON SCI TECHNOL	44	7315	7	14
	0.00	1.00	0.57		Colman BP	2013	...	PLOS ONE	8	0	4	11
	0.00	1.00	0.59		Bondarenko O	2013	...	ARCH TOXICOL	87	1181	4	8
	0.00	1.00	0.55		Tejamaya M	2012	...	ENVIRON SCI TECHNOL	46	7011	5	11
7.55	0.00	1.00	0.57		Li QL	2008	...	WATER RES	42	4591	4	1
	0.00	1.00	0.55		Scown TM	2010	...	CRIT REV TOXICOL	40	653	7	4
	0.00	1.00	0.58		Cheng XK	2004	...	J CHEM ENG DATA	49	675	4	6
	0.01	1.00	1.08		Kang S	2009	...	ENVIRON SCI TECHNOL	43	2648	1	1
9.91	0.00	1.00	0.59		Wijnhoven	2009	...	NANOTOXICOLOGY	3	109	5	3

					SWP							
10.15	0.05	1.65	1.45		Sondi I	2004	...	J COLLOID INTERF SCI	275	177	8	11
	0.10	1.00	1.41		Chen KL	2006	...	ENVIRON SCI TECHNOL	40	1516	8	5
	0.00	1.00	0.55		Yang K	2007	...	ENVIRON POLLUT	145	529	3	0
9.30	0.01	1.08	1.03		Brunner TJ	2006	...	ENVIRON SCI TECHNOL	40	4374	5	15
	0.00	1.00	0.58		Gottschalk F	2013	...	ENVIRON TOXICOL CHEM	32	1278	2	12
	0.01	1.00	1.08		Pal S	2007	...	APPL ENVIRON MICROB	73	1712	5	11
7.68	0.00	1.00	0.54		Muller J	2005	...	TOXICOL APPL PHARM	207	221	7	1
	0.02	1.00	1.56		Jin ZX	2015	...	ENVIRON SCI TECHNOL	49	9168	2	10
7.68	0.37	11.01	1.57		Espinasse B	2007	...	ENVIRON SCI TECHNOL	41	7396	5	7
7.27	0.01	1.06	1.06		Guzman KAD	2006	...	ENVIRON SCI TECHNOL	40	7688	6	5
	0.00	1.00	0.59		Sun YB	2015	...	ENVIRON SCI TECHNOL	49	4255	2	10
10.15	0.00	1.00	0.52		Jafvert CT	2008	...	ENVIRON SCI TECHNOL	42	5945	1	6
10.15	0.00	1.00	0.45		Lu CY	2006	...	CHEM ENG SCI	61	1138	3	0
8.77	0.00	1.00	0.15		Serp P	2003	...	APPL CATAL A-GEN	253	337	8	25
7.90	0.00	1.00	0.15		Tuzen M	2008	...	J HAZARD MATER	152	632	3	28
10.21	0.00	1.00	1.00		Derfus AM	2004	...	NANO LETT	4	11	4	22
	0.05	1.00	1.29		Stampoulis D	2009	...	ENVIRON SCI TECHNOL	43	9473	4	14
	0.00	1.00	0.51		Brant J	2005	...	J NANOPART RES	7	545	3	7
8.26	0.31	9.29	0.90		Velzeboer I	2008	...	ENVIRON TOXICOL CHEM	27	1942	3	8
	0.00	1.00	0.15		Maurer-jones MA	2013	...	ANAL CHEM	85	3036	4	36
9.02	0.02	1.16	0.96		Duncan LK	2008	...	ENVIRON SCI TECHNOL	42	173	2	6
8.26	0.01	1.07	0.93		Pulskamp K	2007	...	TOXICOL LETT	168	58	4	1
	0.00	1.00	0.15		Fu FL	2011	...	J ENVIRON MANAGE	92	407	6	49
	0.00	1.00	0.58		Dale AL	2015	...	ENVIRON SCI TECHNOL	49	2587	2	3
	0.00	1.00	0.58		Hu XG	2013	...	CHEM REV	113	3815	4	13
	0.00	1.00	0.60		Liu L	2013	...	CHEM ENG J	229	444	4	13
	0.00	1.00	1.06		Gupta VK	2011	...	J HAZARD MATER	185	17	5	16
	0.00	1.00	0.52		Zhang XZ	2007	...	CHEMOSPHERE	67	160	8	4
	0.00	1.00	0.55		Gondikas AP	2014	...	ENVIRON SCI TECHNOL	48	5415	1	12
	0.00	1.00	0.59		Wang J	2014	...	ENVIRON SCI TECHNOL	48	4817	3	10
	0.01	1.00	1.06		Holden PA	2016	...	ENVIRON SCI TECHNOL	50	6124	1	2
	0.01	1.00	1.10		Dimkpa CO	2012	...	J NANOPART RES	14	0	5	14
5.75	0.00	1.00	0.54		Xia T	2006	...	NANO LETT	6	1794	5	1
	0.00	1.00	0.62		Fang J	2009	...	ENVIRON POLLUT	157	1101	6	17
	0.01	1.00	1.12		Matranga V	2012	...	MAR ENVIRON RES	76	32	3	4
	0.00	1.00	0.56		Miralles P	2012	...	ENVIRON SCI TECHNOL	46	9224	4	14
	0.02	1.00	1.57		Feriancikova L	2012	...	J HAZARD MATER	235	194	5	13
	0.01	1.00	1.04		Hartmann NB	2010	...	TOXICOLOGY	269	190	6	8
	0.00	1.00	0.90		Tu YS	2013	...	NAT NANOTECHNOL	8	594	4	13

	0.00	1.00	1.00		Xu PA	2012	...	SCI TOTAL ENVIRON	424	1	5	23
	0.06	1.00	1.79		Zhao J	2015	...	ENVIRON SCI TECHNOL	49	2849	2	13
	0.00	1.00	0.77		Duan XG	2015	...	ACS CATAL	5	553	2	21
	0.01	1.00	1.04		Hotze EM	2010	...	J ENVIRON QUAL	39	1909	4	17
	0.00	1.00	0.52		Handy RD	2012	...	ENVIRON TOXICOL CHEM	31	15	2	2
	0.00	1.00	0.53		Lombi E	2013	...	ENVIRON POLLUT	176	193	2	3
	0.00	1.00	0.52		Dhawan A	2006	...	ENVIRON SCI TECHNOL	40	7394	2	6
	0.00	1.00	0.54		Apul OG	2013	...	WATER RES	47	1648	2	10
	0.00	1.00	0.62		Gupta VK	2013	...	ENVIRON SCI POLLUT R	20	2828	3	16
	0.01	1.00	1.10		Chowdhury I	2011	...	J COLLOID INTERF SCI	360	548	4	17
	0.00	1.00	0.62		Saleh TA	2011	...	APPL CATAL B-ENVIRON	106	46	5	16
	0.00	1.00	0.83		Wang YG	2008	...	ENVIRON SCI TECHNOL	42	3588	4	7
	0.02	1.00	1.01		Chen GX	2012	...	ENVIRON SCI TECHNOL	46	7142	3	17
	0.00	1.00	0.62		Saleh TA	2015	...	ENVIRON SCI POLLUT R	22	16721	1	16
	0.00	1.00	0.62		Baker TJ	2014	...	ENVIRON POLLUT	186	257	1	4
	0.00	1.00	0.57		Quik JTK	2012	...	ENVIRON TOXICOL CHEM	31	1019	3	2
	0.00	1.00	0.53		Lombi E	2012	...	ENVIRON SCI TECHNOL	46	9089	3	3
	0.00	1.00	0.15		Ge YG	2011	...	ENVIRON SCI TECHNOL	45	1659	4	53
	0.01	1.00	1.07		Mitrano DM	2015	...	ENVIRON INT	77	132	2	2
	0.00	1.00	0.53		Hassellov M	2008	...	ECOTOXICOLOGY	17	344	4	8
	0.00	1.00	0.15		Wagner S	2014	...	ANGEW CHEM INT EDIT	53	12398	3	30
	0.00	1.00	0.62		Simon P	2008	...	NAT MATER	7	845	3	10
	0.00	1.00	0.58		Thill A	2006	...	ENVIRON SCI TECHNOL	40	6151	4	11
	0.00	1.00	0.56		Sun TY	2016	...	ENVIRON SCI TECHNOL	50	4701	1	2
	0.01	1.00	1.04		Blinova I	2010	...	ENVIRON POLLUT	158	41	7	8
	0.00	1.00	0.55		Chappell MA	2009	...	ENVIRON POLLUT	157	1081	3	9
	0.01	1.00	1.12		Li J	2012	...	ACS APPL MATER INTER	4	4991	5	10
	0.00	1.00	0.53		Whitley AR	2013	...	ENVIRON POLLUT	182	141	4	3
	0.00	1.00	0.61		Chen KL	2010	...	ENVIRON CHEM	7	10	2	9
	0.00	1.00	0.55		Dimkpa CO	2013	...	ENVIRON SCI TECHNOL	47	1082	4	14
	0.00	1.00	1.00		Zhang H	2010	...	ACS NANO	4	380	3	24
	0.00	1.00	0.59		Levard C	2013	...	ENVIRON SCI TECHNOL	47	5738	3	3
	0.01	1.00	1.01		Praetorius A	2014	...	ENVIRON-SCI NANO	1	317	3	3
	0.00	1.00	1.65		Gupta VK	2011	...	WATER RES	45	2207	5	16
	0.00	1.00	1.00		Gong JL	2009	...	J HAZARD MATER	164	1517	8	23
	0.00	1.00	0.60		Froggett SJ	2014	...	PART FIBRE TOXICOL	11	0	3	2
	0.00	1.00	0.15		Asharani PV	2009	...	ACS NANO	3	279	8	41
	0.00	1.00	0.60		Garner KL	2014	...	J NANOPART RES	16	0	3	2
	0.00	1.00	0.77		Sun HQ	2012	...	ACS APPL MATER INTER	4	5466	5	21
	0.00	1.00	0.59		Petersen EJ	2011	...	ENVIRON SCI TECHNOL	45	1133	5	9

	0.00	1.00	0.61		Kim JS	2007	...	NANOMED-NANOTECHNOL	3	95	5	11
	0.00	1.00	0.52		Buffet PE	2011	...	CHEMOSPHERE	84	166	3	8
6.05	0.00	1.00	0.57		Limbach LK	2005	...	ENVIRON SCI TECHNOL	39	9370	5	5
	0.00	1.00	0.58		Priester JH	2012	...	P NATL ACAD SCI USA	109	0	5	14
	0.00	1.00	0.15		Perreault F	2015	...	CHEM SOC REV	44	5861	2	50
	0.00	1.00	0.62		Gupta VK	2012	...	MAT SCI ENG C-MATER	32	12	4	16
	0.00	1.00	0.62		Du WC	2011	...	J ENVIRON MONITOR	13	822	6	14
	0.00	1.00	0.15		Qu XL	2013	...	WATER RES	47	3931	4	51
	0.01	1.00	1.01		Seabra AB	2014	...	CHEM RES TOXICOL	27	159	3	13
6.12	0.03	1.16	0.77		Lu CS	2005	...	WATER RES	39	1183	5	0
	0.00	1.00	0.63		Yao YJ	2012	...	CHEM ENG J	184	326	5	10
	0.00	1.00	0.15		Li D	2008	...	ENVIRON TOXICOL CHEM	27	1888	4	26
	0.00	1.00	0.15		Woan K	2009	...	ADV MATER	21	2233	7	27
	0.00	1.00	0.59		Long ZF	2012	...	ENVIRON SCI TECHNOL	46	8458	4	18
	0.02	1.00	1.02		Liu XY	2009	...	ENVIRON SCI TECHNOL	43	8153	4	7
	0.00	1.00	0.15		Adeleye AS	2014	...	ENVIRON SCI TECHNOL	48	12561	2	29
	0.00	1.00	0.58		Hu M	2013	...	ENVIRON SCI TECHNOL	47	3715	3	7
	0.00	1.00	0.15		Foo KY	2010	...	CHEM ENG J	156	2	6	33
	0.00	1.00	0.91		Petersen EJ	2008	...	ENVIRON SCI TECHNOL	42	3090	3	9
	0.00	1.00	0.51		Zheng L	2005	...	BIOL TRACE ELEM RES	104	83	8	14
	0.00	1.00	1.46		Sun HQ	2014	...	APPL CATAL B-ENVIRON	154	134	3	21
	0.00	1.00	0.60		Handy RD	2012	...	ECOTOXICOLOGY	21	933	4	8
	0.00	1.00	0.53		Zhou DX	2012	...	ENVIRON SCI TECHNOL	46	7520	5	13
	0.00	1.00	0.15		Keller AA	2013	...	ENVIRON SCI TECH LET	1	65	4	37
	0.00	1.00	0.58		Stankovich S	2006	...	NATURE	442	282	7	7
	0.00	1.00	0.62		Wang P	2008	...	SMALL	4	2166	4	7
	0.00	1.00	0.59		Zou YD	2016	...	ENVIRON SCI TECHNOL	50	3658	1	13
	0.00	1.00	0.57		Kohler AR	2008	...	J CLEAN PROD	16	927	5	1
	0.00	1.00	0.57		Peijnenburg W	2015	...	CRIT REV ENV SCI TEC	45	2084	1	2
	0.00	1.00	0.58		Hischier R	2012	...	SCI TOTAL ENVIRON	425	271	5	3
	0.00	1.00	0.56		Zhang SJ	2010	...	WATER RES	44	2067	6	0
	0.00	1.00	0.15		Gong KP	2009	...	SCIENCE	323	760	5	42
	0.00	1.00	0.59		Kiser MA	2010	...	WATER RES	44	4105	2	12
	0.00	1.00	0.15		Park S	2009	...	NAT NANOTECHNOL	4	217	8	44
	0.00	1.00	0.15		Leary R	2011	...	CARBON	49	741	3	45
5.61	0.00	1.00	0.45		Quan X	2005	...	ENVIRON SCI TECHNOL	39	3770	5	19
	0.00	1.00	0.15		Kuhlbusch TAJ	2011	...	PART FIBRE TOXICOL	8	0	5	46
	0.00	1.00	0.62		Domingos RF	2009	...	ENVIRON SCI TECHNOL	43	7277	3	15
	0.00	1.00	0.51		Yi P	2011	...	LANGMUIR	27	3588	6	7
	0.00	1.00	0.52		Guo XK	2013	...	ENVIRON SCI	47	12524	4	9

								TECHNOL				
	0.00	1.00	0.54		Wang ZY	2011	...	ENVIRON SCI TECHNOL	45	6032	5	8
	0.00	1.00	0.55		Tong ZH	2007	...	ENVIRON SCI TECHNOL	41	2985	1	6
	0.00	1.00	0.54		Phenrat T	2007	...	ENVIRON SCI TECHNOL	41	284	3	5
	0.01	1.00	1.12		Yu JG	2014	...	SCI TOTAL ENVIRON	482	241	2	0
	0.01	1.00	1.03		Ren XM	2014	...	ENVIRON SCI TECHNOL	48	5493	3	13
	0.00	1.00	1.04		Saleh TA	2014	...	ADV COLLOID INTERFAC	211	93	2	16
	0.00	1.00	0.58		Yin LY	2011	...	ENVIRON SCI TECHNOL	45	2360	2	8
	0.00	1.00	0.57		Zhu XS	2009	...	J NANOPART RES	11	67	2	4
	0.00	1.00	0.55		Sun YB	2012	...	ENVIRON SCI TECHNOL	46	6020	4	10
	0.06	1.00	0.97		Choi O	2008	...	WATER RES	42	3066	4	11
	0.00	1.00	0.63		Upadhyayula VKK	2009	...	SCI TOTAL ENVIRON	408	1	7	0
	0.00	1.00	0.54		Benn T	2010	...	J ENVIRON QUAL	39	1875	6	3
	0.00	1.00	0.60		Schwab F	2016	...	NANOTOXICOLOGY	10	257	1	2
	0.03	1.00	0.96		Chowdhury I	2015	...	ENVIRON SCI TECHNOL	49	10886	2	13
	0.00	1.00	0.59		Lin DH	2012	...	WATER RES	46	4477	4	8
	0.00	1.00	0.61		Mashayekhi H	2012	...	J COLLOID INTERF SCI	374	111	2	6
	0.00	1.00	1.00		Hardman R	2006	...	ENVIRON HEALTH PERSP	114	165	2	22
	0.00	1.00	0.60		Sharma VK	2009	...	J ENVIRON SCI HEAL A	44	1485	5	8
	0.00	1.00	0.59		Arvidsson R	2011	...	HUM ECOL RISK ASSESS	17	245	3	17
	0.00	1.00	0.15		Yu WW	2003	...	CHEM MATER	15	2854	7	38
	0.02	1.00	1.01		Lin SH	2012	...	LANGMUIR	28	4178	2	17
	0.00	1.00	0.59		Kittler S	2010	...	CHEM MATER	22	4548	4	3
	0.00	1.00	0.57		Aschberger K	2011	...	ENVIRON INT	37	1143	3	2
	0.00	1.00	0.53		Lee CW	2010	...	ENVIRON TOXICOL CHEM	29	669	4	15
	0.02	1.00	0.97		Tourinho PS	2012	...	ENVIRON TOXICOL CHEM	31	1679	2	2
	0.01	1.00	1.08		Cornelis G	2011	...	ENVIRON SCI TECHNOL	45	2777	3	2
	0.00	1.00	0.50		Shvedova AA	2005	...	AM J PHYSIOL-LUNG C	289	0	6	1
	0.00	1.00	1.00		Xiang QJ	2012	...	CHEM SOC REV	41	782	1	24
	0.00	1.00	0.52		Tervonen K	2010	...	ENVIRON TOXICOL CHEM	29	1072	3	9
	0.00	1.00	0.15		Tiede K	2009	...	J CHROMATOGR A	1216	503	2	32
	0.00	1.00	0.47		Donaldson K	2006	...	TOXICOL SCI	92	5	4	1
	0.00	1.00	0.59		Limbach LK	2007	...	ENVIRON SCI TECHNOL	41	4158	4	15
	0.00	1.00	0.61		Scott-fordsmand JJ	2008	...	ECOTOX ENVIRON SAFE	71	616	3	4
	0.00	1.00	0.52		Ferguson PL	2008	...	ENVIRON SCI TECHNOL	42	3879	5	9
	0.00	1.00	0.53		Templeton RC	2006	...	ENVIRON SCI TECHNOL	40	7387	2	9
	0.00	1.00	0.54		Gurr JR	2005	...	TOXICOLOGY	213	66	6	1
	0.00	1.00	0.53		Miao AJ	2010	...	ENVIRON TOXICOL CHEM	29	2814	3	15

	0.00	1.00	0.50		Maynard AD	2004	...	J TOXICOL ENV HEAL A	67	87	4	1
	0.03	1.00	0.88		Barrena R	2009	...	CHEMOSPHERE	75	850	4	14
	0.00	1.00	0.55		Oberdorster G	2007	...	NANOTOXICOLOGY	1	2	4	1
	0.00	1.00	0.47		Fortner JD	2007	...	ENVIRON SCI TECHNOL	41	7497	2	6
	0.00	1.00	0.91		Petersen EJ	2008	...	ENVIRON HEALTH PERSP	116	496	3	9
	0.00	1.00	0.58		Yang K	2006	...	ENVIRON SCI TECHNOL	40	5804	7	18
	0.02	1.00	0.98		Handy RD	2008	...	ECOTOXICOLOGY	17	396	3	4
	0.00	1.00	0.49		Chen W	2008	...	ENVIRON SCI TECHNOL	42	6862	5	0
	0.01	1.00	1.01		Zhu XS	2009	...	NANOTECHNOLOGY	20	0	2	4
	0.01	1.00	1.10		Hall PJ	2010	...	ENERG ENVIRON SCI	3	1238	1	10
	0.00	1.00	0.15		Kasemets K	2009	...	TOXICOL IN VITRO	23	1116	2	54
	0.01	1.00	1.09		Usenko CY	2008	...	TOXICOL APPL PHARM	229	44	3	4
	0.00	1.00	0.51		Kashiwada S	2006	...	ENVIRON HEALTH PERSP	114	1697	2	4
	0.01	1.00	1.07		Lee J	2007	...	ENVIRON SCI TECHNOL	41	2529	2	6
	0.00	1.00	0.55		Wang XK	2005	...	ENVIRON SCI TECHNOL	39	2856	4	0
	0.01	1.00	1.02		Hou WC	2009	...	ENVIRON SCI TECHNOL	43	362	1	5
	0.00	1.00	0.15		Schwarzenbach RP	2003	...	ENV ORGANIC CHEM	0	0	6	39
	0.00	1.00	0.15		Gong D	2001	...	J MATER RES	16	3331	8	40
	0.00	1.00	0.58		Hussain SM	2005	...	TOXICOL IN VITRO	19	975	5	8
	0.00	1.00	0.58		Li QL	2009	...	ENVIRON SCI TECHNOL	43	3574	1	5
	0.00	1.00	0.15		Tasis D	2006	...	CHEM REV	106	1105	4	43
	0.12	1.00	1.85		Saleh N	2008	...	ENVIRON SCI TECHNOL	42	3349	2	5
	0.00	1.00	0.50		Shvedova AA	2008	...	AM J PHYSIOL-LUNG C	295	0	2	1
	0.00	1.00	0.15		Griffitt RJ	2007	...	ENVIRON SCI TECHNOL	41	8178	2	47
	0.00	1.00	0.53		Chen KL	2009	...	ENVIRON SCI TECHNOL	43	7270	1	5
	0.00	1.00	0.15		Lewinski N	2008	...	SMALL	4	26	2	52
	0.00	1.00	0.60		Hotze EM	2008	...	ENVIRON SCI TECHNOL	42	4175	1	6
	0.00	1.00	0.61		Kang S	2008	...	ENVIRON SCI TECHNOL	42	7528	2	1
	0.00	1.00	0.15		Asahi R	2001	...	SCIENCE	293	269	8	55
	0.00	1.00	0.15		Baughman RH	2002	...	SCIENCE	297	787	8	35
	0.00	1.00	0.55		Helland A	2007	...	ENVIRON HEALTH PERSP	115	1125	3	9
	0.02	1.00	1.39		Takagi A	2008	...	J TOXICOL SCI	33	105	2	1
	0.00	1.00	0.58		Chen JY	2008	...	ENVIRON SCI TECHNOL	42	7225	2	18
	0.00	1.00	0.54		Johnson RL	2009	...	ENVIRON SCI TECHNOL	43	5455	1	5
	0.00	1.00	0.59		Chen CL	2006	...	IND ENG CHEM RES	45	9144	4	0
	0.02	1.00	0.92		Li YH	2003	...	CARBON	41	2787	7	0
	0.00	1.00	1.42		Fujishima A	2000	...	J PHOTOCH PHOTOBIO C	1	1	7	19
	0.16	1.00	1.43		Cui DX	2005	...	TOXICOL LETT	155	73	1	1
	0.00	1.00	0.77		Kasuga T	1999	...	ADV MATER	11	1307	8	20

	0.00	1.00	1.04		Ju XS	2002	...	J MEMBRANE SCI	202	63	5	19
	0.00	1.00	1.46		Chen Q	2002	...	ADV MATER	14	1208	5	20
	0.00	1.00	0.82		Li YH	2003	...	MATER RES BULL	38	469	4	0
	0.00	1.00	0.54		Oberdorster G	2005	...	PART FIBRE TOXICOL	2	8	2	1
	0.00	1.00	0.77		Tsai CC	2004	...	CHEM MATER	16	4352	3	20
	0.00	1.00	1.04		Zhang HZ	2001	...	NANO LETT	1	81	6	19
	0.00	1.00	1.04		Cao YA	2004	...	NEW J CHEM	28	218	3	19
